# USP28 promotes tumorigenesis and cisplatin resistance by deubiquitinating MAST1 protein in cancer cells

**DOI:** 10.1007/s00018-024-05187-2

**Published:** 2024-03-18

**Authors:** Janardhan Keshav Karapurkar, Jencia Carminha Colaco, Bharathi Suresh, Apoorvi Tyagi, Sang Hyeon Woo, Won-Jun Jo, Nare Ko, Vijai Singh, Seok-Ho Hong, Seung Jun Oh, Kye-Seong Kim, Suresh Ramakrishna

**Affiliations:** 1 Graduate School of Biomedical Science and Engineering, Hanyang University, Seoul, 04763 South Korea; 2 Biomedical Research Center, Asan Institute for Life Sciences, Seoul, 05505 Korea; 3 Department of Nuclear Medicine, Asan Medical Center, University of Ulsan College of Medicine, Seoul, 05505 Korea; 4 Department of Biosciences, School of Science, Indrashil University, Rajpur, Mehsana, Gujarat India; 5 Department of Internal Medicine, School of Medicine, Kangwon National University, Chuncheon, South Korea; 6 College of Medicine, Hanyang University, Seoul, 04763 South Korea

**Keywords:** Anti-tumor activity, Clinical histology, In vivo drug delivery, Mouse xenograft, Tumor recurrence, Therapeutics

## Abstract

Cisplatin is a chemotherapy drug that causes a plethora of DNA lesions and inhibits DNA transcription and replication, resulting in the induction of apoptosis in cancer cells. However, over time, patients develop resistance to cisplatin due to repeated treatment and thus the treatment efficacy is limited. Therefore, identifying an alternative therapeutic strategy combining cisplatin treatment along with targeting factors that drive cisplatin resistance is needed. CRISPR/Cas9 system-based genome-wide screening for the deubiquitinating enzyme (DUB) subfamily identified USP28 as a potential DUB that governs cisplatin resistance. USP28 regulates the protein level of microtubule-associated serine/threonine kinase 1 (MAST1), a common kinase whose expression is elevated in several cisplatin-resistant cancer cells. The expression level and protein turnover of MAST1 is a major factor driving cisplatin resistance in many cancer types. Here we report that the USP28 interacts and extends the half-life of MAST1 protein by its deubiquitinating activity. The expression pattern of USP28 and MAST1 showed a positive correlation across a panel of tested cancer cell lines and human clinical tissues. Additionally, CRISPR/Cas9-mediated gene knockout of USP28 in A549 and NCI-H1299 cells blocked MAST1-driven cisplatin resistance, resulting in suppressed cell proliferation, colony formation ability, migration and invasion in vitro. Finally, loss of USP28 destabilized MAST1 protein and attenuated tumor growth by sensitizing cells to cisplatin treatment in mouse xenograft model. We envision that targeting the USP28-MAST1 axis along with cisplatin treatment might be an alternative therapeutic strategy to overcome cisplatin resistance in cancer patients.

**Supplementary Information:**

The online version contains supplementary material available at 10.1007/s00018-024-05187-2.

## Introduction

The current strategies for treating cancer involve surgery, chemotherapy and radiotherapy. Among them chemotherapy is widely used for cancer treatment and includes neoadjuvant, adjuvant and combination chemotherapies [1]. The platinum-based drug cisplatin (cis-diaminedichloroplatinum) is one of the most effective anti-cancer agents and is a standard treatment for a wide range of cancer types [2–4]. Cisplatin goes through a process called aquation during which cisplatin becomes highly reactive where one or two chlorides in cisplatin will be replaced with water molecule inside the cell. In this process, intrastrand DNA crosslinks between two purine bases on the same strand are the major DNA lesions, accounting for over 90% of the cisplatin-induced lesions. Nevertheless, interstrand crosslinks between two purine bases on the opposing strand are the minor DNA lesions account for less than 5% of the cisplatin-induced lesions [5, 6]. Cisplatin-induced DNA adducts results in long-term DNA damage response and eventually induce massive cell death and activates apoptotic pathway [7]. Despite its ability to kill tumor cells, repeated treatment with cisplatin can lead to acquisition of intrinsic and acquired resistance, which limits its clinical use [8–10]. To resist the cisplatin therapy, cells must eliminate or tolerate the cisplatin-induced DNA lesions in a variety of ways, including enhancing drug efflux, lowering drug uptake, or triggering drug detoxification through covalent binding to metalloproteins or glutathione [11–14]. Therefore, a new strategy to overcome this limitation is essential to effectively treat patients who acquire cisplatin resistance.

A combination of cisplatin and targeted factors that drive cisplatin resistance can be used to combat acquired resistance in many cancers. Several protein kinases such as RSK [15], SRPK1 [16], and MKP-1 [17] were shown to participate in the cisplatin-resistance mechanism that allows cancer cells to escape from cisplatin cytotoxicity. Microtubule-associated serine/threonine-protein kinase 1 (MAST1) was recently reported as a key driving factor in the development of cisplatin resistance in many cancer types [18]. MAST1 expression was high in cisplatin-resistant primary tumors from patients under cisplatin-based treatment. The combination of cisplatin treatment with lestaurtinib, a MAST1 inhibitor, potentially inhibited MAST1 kinase activity and sensitized several cancer types to cisplatin [18–20]. To develop a new strategy to overcome cisplatin resistance, we recently performed genome-wide screening for deubiquitinating enzymes (DUBs), and the results showed that DUBs promotes MAST1 protein stabilization by regulating its protein abundance in cisplatin-resistant cancer cells [21].

DUBs have emerged as key regulators of DNA damage and are critical in the development of drug resistance in many cancers [22]. In our recent study, we down-regulated genome-wide DUBs by CRISPR/Cas9 system and identified potential DUBs that enhance cisplatin-mediated cytotoxicity and induce cell death in cisplatin-resistant cancer cells [21]. USP28 was emerged as one of the potential candidates involved in cisplatin resistance [21]. USP28 exhibits diverse biological functions including cellular proliferation, apoptosis, DNA damage repair and oncogenesis [23]. During DNA damage, the catalytic activity of USP28 is increased due to the phosphorylation of USP28 by the kinase ATM [24]. A recent study reported that the inhibition of USP28 by specific inhibitors suppresses its enzymatic activity, thereby sensitizing cisplatin-resistant cancer cells to cisplatin treatment [25].

In this study, we investigated the molecular mechanism by which USP28 regulates MAST1 protein abundance during acquisition of cisplatin resistance. We demonstrated that USP28 interacts and regulate MAST1 protein stabilization in cisplatin-resistant cancer cells. USP28 deubiquitinates MAST1 protein and extends its half-life. We further demonstrated that knockout of USP28 in cisplatin-resistant cancer cells suppressed cell proliferation, migration, invasion and colony formation ability. Additionally, loss of USP28 attenuated tumor growth in a mouse xenograft model. Thus, we envision that inhibiting the regulation of MAST1 protein abundance by USP28 in cisplatin-resistant cancer cells may be effective alternative therapeutic strategy to overcome cisplatin resistance in cancer patients.

## Materials and methods

### Cell culture and transfection

HEK293 (KCLB: 21573), HeLa (KCLB:10002), A549 (KCLB: 10185) and H1299 (KCLB: 25803) cells were maintained in DMEM (Gibco BRL, Rockville, MD, USA) supplemented with 10% fetal bovine serum (FBS; Gibco) and 1% penicillin and streptomycin (Gibco) at 37 °C in a humidified atmosphere with 5% CO_2_. The cells were passaged every 3–4 days. Cells at 60–80% confluence were transfected with the indicated constructs using polyethyleimine (PEI) (Polysciences, Inc. Cat no. 24765), Lipofectamine 3000 (Cat no. L3000001, Thermo Fisher Scientific) or Lipofectamine 2000 (Cat no. 11668019 Life Technologies), following the manufacturers’ instructions. The cells were harvested 36–48 h post transfection for further analysis.

### Plasmids and sgRNAs

The Myc-MAST1 plasmid was generated as described previously [21]. Briefly, the human MAST1 gene was amplified from cDNA and cloned into the pCDNA3 6XMyc-vector using BamHI and XbaI restriction sites. Flag-tagged USP28 (Addgene #15665) and HA-tagged Ubiquitin (Addgene #18712) plasmids were purchased from Addgene. The catalytic mutant of USP28 was generated using site-directed mutagenesis by substituting cysteine with alanine at position 171; the resultant vector was named Flag-USP28C171A. The plasmid encoding Cas9-2a-mRFP-2a-PAC (puromycin N-acetyl-transferase puromycin resistance gene) and plasmid encoding sgRNAs were purchased from Toolgen (Seoul, South Korea). To generate sgRNAs targeting potential DUB candidates, sgRNA target sequences were designed using a public tool (www.broadinstitute.org) and cloned into vectors as described previously [26]. Briefly, oligonucleotides containing the USP28 target sequence were synthesized (Bioneer, Seoul, South Korea), and T4 polynucleotide kinase was used to add terminal phosphates to the annealed oligonucleotides (Bio-Rad, CA, USA). The vector was digested using BsaI restriction enzyme and ligated with the annealed oligonucleotides. The target sequences for the sgRNAs targeting USP28 are listed in Supplementary Table S1.

### Antibodies and reagents

Mouse monoclonal antibodies against Flag (Anti-DDDDK-tag, M185-3L, 1:1,000) were purchased from MBL Life Science, and phospho-Histone H2AX (Ser139) (Merck, 05-636) was purchased from Millipore. Mouse monoclonal antibodies against MAST1 (sc-373845, 1:50), H2AX (sc-517336; 1:1000), c-Myc (SC-40, 1:1,000), ubiquitin (sc-8017, 1:1,000), HA (sc-7392, 1:1,000), GAPDH (sc-32233, 1:1000), normal mouse IgG (sc-2025, 1:1000), MEK1 (sc-219, 1: 1000), ERK1/2 (sc-514302, 1:1000), p-ERK 1/2 (sc-81492, 1:1000), and BIM (H5) (sc-374358, 1:1000) were purchased from Santa Cruz Biotechnology. Rabbit polyclonal antibodies against MAST1 (Cusabio CSB-PA897529LA01HU, 1:1000), USP28 (Proteintech 17707-1-AP, 1:1500) and 488/594-conjugated secondary antibodies (Cat. no. A21207 and Cat. no. A21203, 1:200; Life Technologies) were used. p-MEK1 (Cat no. 9121, 1: 1000) and cleaved PARP (D64E10, 1: 1000) were purchased from Cell Signaling Technology.

IP lysis buffer (Cat. no. 87787; Thermo Fisher), cell lysis buffer (Cat. no. R2002, Biosesang), protein 5X sample buffer (Cat. no. EBA-1052, Elpis Biotech), Protein A/G Plus agarose beads (sc-2003, Santa Cruz Biotechnology), protease inhibitor cocktail (Cat. no. 11836153001, Roche), the protein translation inhibitor cycloheximide (CHX; Cat. no. 239765, Merck), the proteasomal inhibitor MG132 (Cat. no. S2619, Selleckchem), puromycin (Cat. no. 12122530, Gibco), cisplatin (Cat no. P4394, Sigma-Aldrich), the DUB inhibitor PR-619 (ab144641, Abcam), AZ1 USP25/28 inhibitor (Cat. no. 7845, Tocris), CCK-8 assay reagent (Dojindo Molecular Technologies, MD, USA) and DAPI (Cat. no. H-1200, Vector Laboratories) were purchased and used.

### Cell viability assay for dose response curve

The relative viability of the cells post cisplatin treatment was measured using a Cell Counting Kit-8 (CCK8; Dojindo, Kumamoto, Japan). Cells were seeded in 96-well plates at density of 1 × 10^4^ s per well and were grown for 24 h. The cells were treated with increasing concentration of cisplatin for 48 h. Later, 10 μL of CCK-8 assay solution was added and incubated for 4 h. Absorbance was recorded at 450 nm using spectrophotometer (Bio-Rad Laboratories, Inc, Korea). IC_50_ and IC_80_ value of cisplatin in HeLa (IC_50_ = 6.3 µg/mL and IC_80_–15 µg/mL), A549 (IC_50_ = 3.5 µg/mL and IC_80_–13 µg/mL) and H1299 (IC_50_ = 6.6 µg/mL and IC_80_–19.5 µg/mL) cells were calculated.

### Generation of cisplatin resistance HeLa cell line

HeLa Cisplatin-resistant cells (HeLa-cisR) were derived from their respective parental cell lines by gradual exposure to cisplatin which is dissolved in 0.9% Saline (Sigma-Aldrich, UK). Briefly, the HeLa cells were seeded at a density of 1 × 10^6^ and subjected to stepwise increases upto 15 μg of cisplatin/mL over a period of 6 months. The cisR cell lines were grown as monolayer cultures and maintained in the DMEM medium containing cisplatin (5 μg/mL) and supplemented with 10% FBS (Gibco) and 1% penicillin and streptomycin (Gibco) at 37 °C in a humidified atmosphere with 5% CO_2_.

### T7 endonuclease 1 assay

Genomic DNA was isolated using DNeasy Blood & Tissue kits (Promega, Madison, WI, USA) according to the manufacturer’s protocol. The region of DNA containing the nuclease target site was PCR-amplified and denatured by heating and annealed to form heteroduplex DNA, which was then treated with 5 units of T7E1 (New England Biolabs, MA, USA) for 15 to 20 min at 37 °C, followed by 2% agarose gel electrophoresis. Mutation frequencies were calculated based on band intensity using ImageJ software and the following equation: mutation frequency (%) = 100 × (1 − [1 − fraction cleaved]1/2), where the fraction cleaved was the total relative density of the cleavage bands divided by the sum of the relative density of the cleaved and uncut bands. The oligonucleotide sequences used for PCR amplification for the T7E1 assay are listed in Supplementary Table S2. The PCR-amplicon sizes of the USP28 gene and the expected cleavage sizes after the T7E1 assay are summarized in Supplementary Table S3.

### Real-time PCR

Total RNA was isolated using Trizol reagent (Favorgen, Kaohsiung, Taiwan). The reverse transcription reaction was performed using a SuperScript III First-Strand Synthesis System (Life Technologies, USA) with an oligo-dT primer according to the manufacturer’s protocol. Quantitative PCR was performed in triplicate using Fast SYBR Green I Master Mix (Life Technologies) and a Step One Plus Real-Time PCR System (Life Technologies). The oligonucleotide sequences used for qRT-PCR are mentioned in Supplementary Table S4.

### Generation of a USP28-knockout cell line using CRISPR/Cas9

The single cell–derived USP28 KO clones were generated using the CRISPR/Cas9 system as described previously [27, 28]. A549 and H1299 cells were co-transfected with a plasmid encoding Cas9 and sgRNA2 targeting USP28 or scrambled sgRNA (mock control) at a 1:2 ratio using Lipofectamine 3000 Reagent or Lipofectamine 2000 reagent, respectively, according to the manufacturer’s instructions. Later, the cells were selected using puromycin (1 µg/mL) for next 2 days. The puromycin-selected cells were seeded into 96-well plates at 25 cells/plate and incubated in a CO_2_ incubator at 37 °C. After 15 days, the wells were microscopically evaluated and the single cell–derived colonies were selected. The selected colonies were dissociated using trypsin–EDTA and reseeded into 24-well cell culture plates. A small portion of selected colonies was used to isolate genomic DNA and screened for USP28 disruption by T7E1 assay. The T7E1 positive single cell–derived clones were expanded and stored in a liquid nitrogen tank after Sanger sequencing. USP28 mRNA and protein levels in USP28-KO clones were determined by RT-PCR and western blotting, respectively. Cell lines showing complete reduction in USP28 mRNA and protein levels were used for in vitro and in vivo experiments.

### Immunoprecipitation

Cells were transfected with the indicated DNA constructs. At 36–48 h post transfection, the cells were lysed in IP lysis buffer ((25 mM Tris–HCl (pH 7.4), 150 mM sodium chloride, 1 mM EDTA, 1% NP-40, 5% glycerol, 1 mM PMSF, and protease inhibitor cocktail) for 20 min and the amount of protein was estimated using Bradford reagent. Cell lysate (2–3 mg) was immunoprecipitated using the indicated antibodies at 4 °C overnight and then incubated with 35 μL of protein agarose beads at 4 °C for 3 h. The agarose beads were washed with lysis buffer and eluted in 2X SDS sample loading buffer (5X SDS sample loading buffer containing 4% SDS, 20% glycerol, 10% 2-mercaptoethanol, 0.004% bromophenol blue, and 0.125 M Tris–HCl [pH 6]). The eluted samples were boiled at 95 °C–100 °C for 5 min and separated on SDS-PAGE gels by western blotting. Mouse IgG (ab-99697, 1: 10,000; Abcam) and rabbit IgG (CST-58802S, 1: 10,000; Cell Signaling Technology) light chain-specific secondary antibody was used to prevent interference from heavy and light immunoglobulin chains in the binding assay.

### Tandem ubiquitin-binding entities assay

The ubiquitination status of MAST1 protein was determined using a tandem ubiquitin binding entities (TUBEs) assay (Cat. no. UM402, LifeSensors, PA, USA) as previously described [29]. The mock control, USP28-KO A549 and USP28-KO H1299 cells were pretreated with the proteasome inhibitor MG132 (10 μM/mL) for 6 h to accumulate polyubiquitinated MAST1 protein. Cells were lysed in IP lysis buffer containing 150 mM sodium chloride, 1% Triton X-100, 25 mM Tris (pH 7.5), 1 mM EDTA, 10% glycerol, and protease inhibitor cocktail. The lysed protein extracts were incubated with 20 µL of ubiquitin affinity matrices-TUBE2 at 4 °C for 3 h with rotation. The beads were washed with IP lysis buffer and samples were eluted in 30 µL 2X SDS sample loading buffer (5X SDS sample loading buffer containing 4% SDS, 20% glycerol, 10% 2-mercaptoethanol, 0.004% bromophenol blue, 0.125 M Tris–HCl (pH 6.8)). The eluted samples were boiled at 95 °C–100 °C for 5 min, separated by SDS-PAGE and analyzed by western blotting.

### Deubiquitination assay

The DUB activity of USP28 against endogenous and exogenous MAST1 protein was determined in A549 and HEK293 cells, respectively. The cells were treated with MG132 (10 µM/mL for 6 h) 48 h post transfection and harvested. The cells were lysed for 20 min in denaturing lysis buffer containing 150 mM sodium chloride, 1% Triton X-100, 1% sodium deoxycholate, 1% SDS, 50 mM Tris–HCl (pH 7.4), 2 mM EDTA, 1 mM PMSF, and protease inhibitor cocktail. Cell lysates (2–3 mg) were immunoprecipitated with the respective antibodies at 4 °C overnight and incubated with 35 μL of protein agarose beads for 2–3 h at 4 °C. The agarose beads were washed with lysis buffer and samples were eluted in 2X SDS sample loading buffer (5X SDS sample loading buffer containing 4% SDS, 20% glycerol, 10% 2-mercaptoethanol, 0.004% bromophenol blue, and 0.125 M Tris–HCl [pH 6]). The eluted samples were boiled at 95 °C–100 °C for 5 min, separated on SDS-PAGE gels and analyzed by western blotting using anti-ubiquitin and anti-HA antibodies. To avoid non-specific binding of polyubiquitin molecules to MAST1 protein, the protein-bound agarose beads were washed with lysis buffer containing 300 mM NaCl as previously described [30].

### Immunofluorescence staining

A549 and H1299 cells were grown on glass coverslips and incubated at 37 °C in a humidified atmosphere with 5% CO_2_. The cells were washed with phosphate-buffered saline (PBS, Gibco), fixed for 15 min using 4% paraformaldehyde (PFA, Biosesang) and permeabilized in PBS containing 0.1% Triton X for 5 min at room temperature. The cells were washed, blocked in 3% bovine serum albumin and stained with indicated primary antibodies overnight at 4 °C. The next day, the cells were washed with PBS and incubated with Alexa Fluor 488–conjugated secondary antibodies for 1 h. The nuclei were stained with DAPI and cells were mounted using VectaShield (Vector Laboratories, CA, USA). The cells were then visualized and images were produced using a Leica fluorescence microscope (Leica, DM 5000B; Leica CTR 5000; Wetzlar, Germany).

### Duolink proximity ligation assay (PLA)

The interaction between USP28 and MAST1 was observed using a Duolink in situ proximity ligation assay (PLA) kit (Cat. no. DUO92101, Sigma Aldrich) according to the manufacturer’s instructions. A549 cells were fixed in 4% PFA for 10 min at room temperature and then blocked with blocking solution. The cells were treated with primary antibodies targeting MAST1 and USP28 for 1 h at 37 °C, followed by incubation with PLA probes for 1 h at 37 °C in a humidified chamber. After three washes, ligation ligase solution was added, and the cells were incubated for 30 min at 37 °C. The slides were incubated for 100 min in an amplified polymerase solution at 37 °C in the dark. Finally, the cells were stained with mounting medium containing DAPI. A Leica fluorescence microscope was used to capture the fluorescence images (Leica, DM 5000B; Leica CTR 5000; Wetzlar, Germany).

### Immunohistochemistry (IHC)

Clinical tissue microarray slides of lung, colon and breast tumors were purchased from ISU Abxis (Gyeonggi-do, South Korea). Formalin-fixed, paraffin-embedded (FFPE) tissue samples were processed and incubated with USP28 or MAST1 antibody according to the supplier’s protocol. The samples were counterstained with hematoxylin, dehydrated, and mounted. The staining intensity was determined using ImageJ IHC profiler, an open source plugin for quantification and scoring of IHC images [31]. The staining was scored as 0 (no stain), + 1 (weak stain), + 2 (moderate stain) and + 3 (strong stain) based on the intensity of staining. The results of multiplying the percentage of cells with staining intensity values were added to calculate the H-score. The relationship between the protein expression level of USP28 and MAST1 in different tissues was analyzed using non-parametric Spearman correlation test in order to test the significance of combined tissue IHC-expression.

Tumor tissue xenografts obtained from mice were fixed with 4% PFA and embedded in paraffin. FFPE tissues were then sectioned at a thickness of 5 µm and stained with USP28 and MAST1 following the manufacturers’ recommendations. The mounted IHC tissue samples were visualized and images were produced using a Leica DM5000 B microscope (Leica, Germany).

### Cell proliferation assay

A549 cells (mock control, USP28KO, USP28KO-reconstituted with USP28 or MAST1) and H1299 cells (mock control, USP28KO, USP28KO-reconstituted with USP28 or MAST1) were treated with either vehicle (saline) or cisplatin for 48 h. Next, 10 μL of CCK-8 assay reagent (Dojindo Molecular Technologies, MD, USA) was added to each well, and absorbance was measured at 450 nm using a spectrophotometer (Bio-Rad Laboratories, Inc., Korea). The concentrations of cisplatin used for A549 and H1299 cells were 2 μg/mL and 5 μg/mL, respectively.

### Apoptosis assay

The Annexin-V/PI (Propidium Iodide) population was detected using a BD FACSCanto II flow cytometer (BD Biosciences, CA, USA). Briefly, A549 cells (mock control, USP28KO, USP28KO-reconstituted with USP28 or MAST1) were treated with either vehicle (saline) or cisplatin for 48 h. The cells were then harvested and washed twice with PBS containing 10% FBS. The cells were counted, and 5 µL of Annexin-V and PI (BD Pharmingen™ FITC Annexin V apoptosis detection kit, Cat. no. 556547, BD biosciences, San Diego, CA, USA) were added to cells, followed by incubation for 15 min. The stained cells were resuspended in binding buffer, and flow cytometry was performed within 1 h.

### Cell cycle analysis

Cell cycle analysis was performed by PI staining (BD Biosciences). A549 cells (mock control, USP28KO, USP28KO-reconstituted with USP28 or MAST1) and H1299 cells (mock control, USP28KO, USP28KO-reconstituted with USP28 or MAST1) were treated with vehicle or cisplatin for 48 h and then harvested, washed twice with ice-cold PBS containing 10% FBS, and fixed with ice-cold 70% ethanol. The cells were resuspended in PI (50 µg/mL; Sigma) and RNase A (200 µg/mL, New England Biolabs, MA, USA) and subjected to FACS analysis (BD FACSCanto II, BD Biosciences) to measure DNA content. Data were analyzed using FACS Diva software (version 8, BD bioscience). Next, 10 μL of CCK-8 assay reagent (Dojindo Molecular Technologies, MD, USA) was added to each well, and absorbance was measured at 450 nm using a spectrophotometer (Bio-Rad Laboratories). The concentrations of cisplatin used for A549 and H1299 cells were 2 μg/mL and 5 μg/mL, respectively.

### Soft agar assay

A549 cells (mock control, USP28KO, USP28KO-reconstituted with USP28 or MAST1) and H1299 cells (mock control, USP28KO, USP28KO-reconstituted with USP28 or MAST1) were examined by colony formation assay. Firstly, 1% agarose gel and 1X complete DMEM were mixed at a ratio of 1:1 and plated onto 35 mm culture dishes. The plates were then incubated overnight. Cells resuspended in 0.75% agarose with DMEM (1:1 ratio) were seeded at a density of 1 × 10^4^ cells per well. The cells were treated with vehicle or cisplatin every other day for 14 days. Crystal violet dye (0.01%) diluted in 20% methanol was used to stain the anchorage-independent colonies, and colonies were counted using a light microscope (IX71, Olympus, Tokyo, Japan).

### Wound healing assay

A549 cells (mock control, USP28KO, USP28KO-reconstituted with USP28 or MAST1) and H1299 cells (mock control, USP28KO, USP28KO-reconstituted with USP28 or MAST1) were cultured to near 90% confluence. Scratches were made in the monolayers with a sterile pipette tip. The wounded cell layer was washed with PBS and plates were incubated in medium containing either vehicle or cisplatin at 37 °C with 5% CO_2_. Wound closure was compared at 0 h and 24 h post-scratch using a light microscope and quantified using ImageJ software.

### Transwell cell invasion assay

Transwell chambers (0.8 µm pore) were coated with Matrigel for 1 h at 37 °C (Corning, NY, USA) according to the manufacturer’s instructions. A549 cells (mock control, USP28KO, USP28KO-reconstituted with USP28 or MAST1) and H1299 cells (mock control, USP28KO, USP28KO-reconstituted with USP28 or MAST1) were seeded at a density of 3.0 × 10^4^ cells per well in 500 µL of serum-free DMEM in the top chamber. Next, 750 µL of complete media containing either vehicle or cisplatin was added to the bottom chambers. Plates were then incubated at 37 °C with 5% CO_2_ for 24 h. Cells on the top surface of the insert were scraped off, and the cells on the bottom surface were fixed with ice-cold methanol followed by crystal violet staining. Cells were visualized and images were produced using a Leica DM5000 B microscope. The number of cells was counted using ImageJ, and the data are presented graphically.

### Animal studies

The animal study was approved by the Institutional Animal Care and Use Committees of Hanyang University.

*Study design:* The four groups including Mock, USP28-KO, USP28-KO cells reconstituted with USP28 and USP28-KO cells reconstituted with MAST1.

*Treatment strategy:* Cisplatin (2 mg/kg) prepared in saline (Vehicle) was delivered by intraperitoneal injection (i.p.) twice a week for 14–16 days.

*Animal species or strain:* Mouse/ NOD scid gamma/ NOD.Cg-Prkdc^scid^ IL2rg^tm1Wjl^/SzJ (005557).

*Animal sex:* 2 males and 2 females per group.

*Age and weight:* 6-week old mice weighing between 20 and 25 g.

*Sample size:* Four mice per group (n = 4).

*Total number of animals used in this study:* Total four groups, four mice per group (n = 4). Total 16 mice were used in this study.

*Inclusion and exclusion:* No mice were excluded from the study.

*Randomization:* The animal house staff with no prior knowledge of experiment to be performed randomly allocated age matched mice of equivalent genetic background for the study.

*Blinding:* The animal house staff allocated mice for the study. For this study, three different researchers were involved as follows: First leading investigator performed subcutaneous injection of samples from each group. Second leading investigator performed cisplatin treatment. Third leading investigator conducted euthanization and surgical procedure.

*Drug:* Cisplatin; *Vehicle:* Saline; *Dose:* Cisplatin (2 mg/kg); *Cell lines:* A549 cells.

*Site and route of administration:* Cell were subcutaneously injected into the right flank of each mice. Cisplatin was delivered by intraperitoneal injection (i.p.) twice a week for 14–16 days.

*Detailed protocol:* Mice were housed in a temperature-controlled room under standard conditions (12 h light/dark cycle at a temperature of 27 °C and 55% relative humidity) with access to food and water ad libitum. A549 cells (1.0 × 10^7^) transfected with Mock control, USP28-KO, USP28-KO reconstituted with USP28 or MAST1 were prepared in DMEM: Matrigel (1:1) (BD Biosciences) and subcutaneously injected into the right flank of each mouse (four mice per group, n = 4). Cisplatin (2 mg/kg) was delivered by intraperitoneal injection (i.p.) twice a week from the day 7 till the end of the experiment. Mice were weighed two times a week and the experiment was terminated on the 27th day of the treatment. At the end of the study, all mice were euthanized. The tumors were harvested at the end of the experiment and images were taken.

*Method of euthanasia:* CO_2_ asphyxiation and tumors were collected by dissection.

*Outcome measures:* Tumor growth was recorded by measuring two perpendicular diameters (short axis and long axis) and tumor volume was calculated using the formula V = D × d2 × 0.5, where D and d are the long and short axes of the tumor, respectively. Tumor volume was measured every other day and is presented graphically. Mice were weighed two times a week and the experiment was terminated on the 27th day of the treatment.

*Statistical methods:* Statistical analysis and graphical presentation were performed using GraphPad Prism 9.0. Two-way ANOVA followed by Tukey's post-hoc test was used with the indicated *P* values. Statistical power is 80% and was calculated post experiment using G*Power software (https://www.psychologie.hhu.de/arbeitsgruppen/allgemeine-psychologie-und-arbeitspsychologie/gpower.html) [32].

### Statistical analysis

Statistical analysis and graphical presentation were performed using GraphPad Prism 9.0. All results are presented as the means and standard deviations of at least three independent experiments (unless otherwise stated in the figure legends). The error bar represents means and standard deviations. Comparisons between two groups were analyzed using Student’s t-test. Experiments involving three or more groups were analyzed by one-way or two-way analysis of variance (ANOVA) followed by Tukey’s post hoc test. The relationship between the protein expression level of USP28 and MAST1 in different human tissues was determined by H-score and analyzed using non-parametric Spearman correlation test. *P*-values < 0.05 were regarded as statistically significant. IC_50_ and IC_80_ values of cisplatin in HeLa, A549 and H1299 cells were calculated by non-linear regression analysis.

## Results

### USP28 is a deubiquitinase for MAST1

**Fig. 1:**
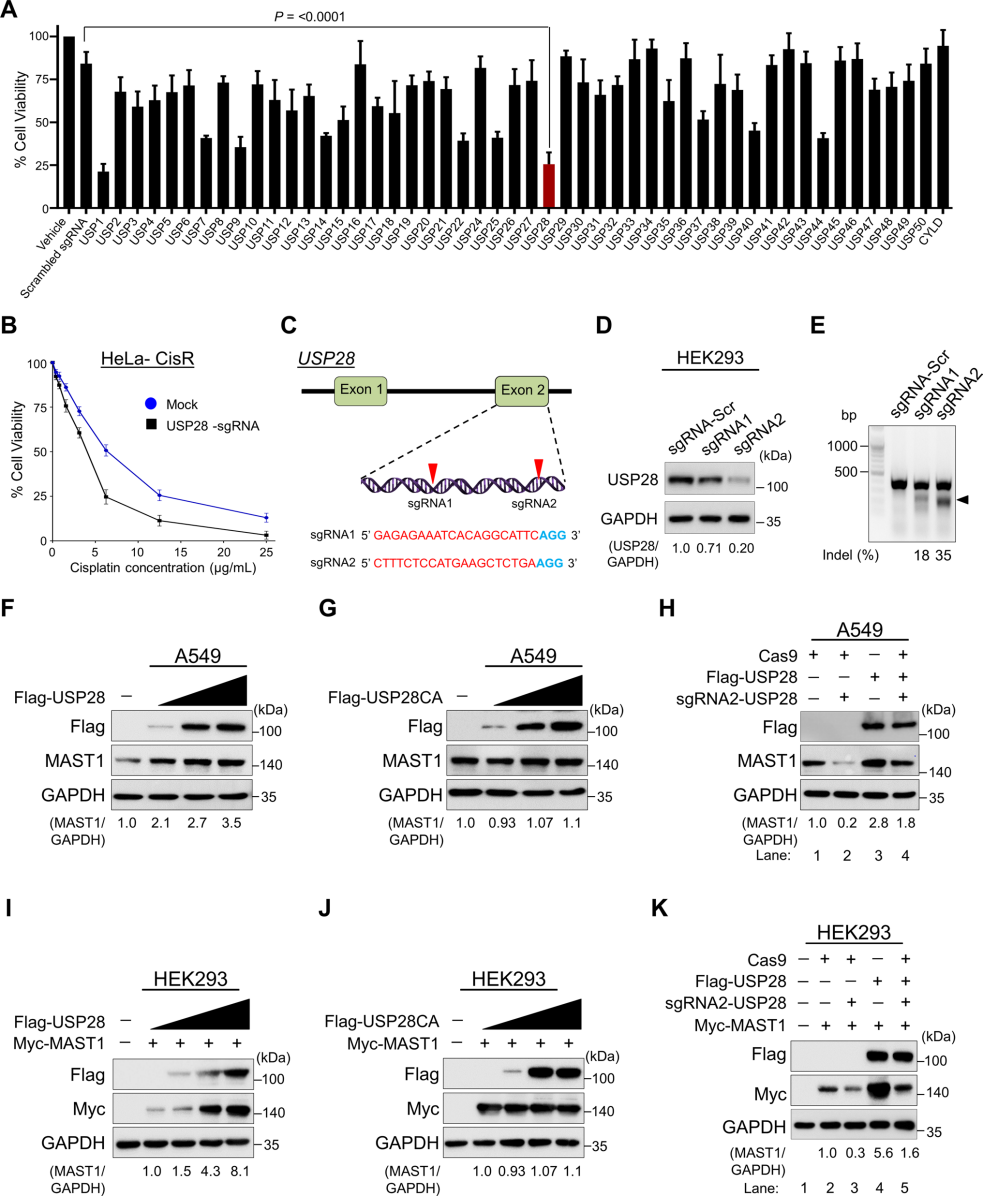
USP28 regulates MAST1 protein levels. **A** Transfection of an entire set of sgRNAs targeting individual USP subfamily genes along with Cas9 nuclease into cisplatin-resistant HeLa cells (HeLa-cisR). Transfected cells were treated with a sub-lethal dose (5 µg/mL) of cisplatin. HeLa-cisR cells treated with saline served as the negative control (vehicle) and cisplatin-treated HeLa-cisR cells co-transfected with scrambled sgRNA and Cas9 served as the mock control. Cisplatin-induced cell death was estimated using a cell viability assay and represented as a graph. Data are presented as the mean and standard deviation of three independent experiments (n = 3). **B** The USP28-depleted cells were treated with an increasing concentrations of cisplatin (5 µg/mL, 10 µg/mL, 15 µg/mL, 20 µg/mL and 25 µg/mL) for 48 h, and cell viability was measured. The IC_50_ values of cisplatin in HeLa-CisR mock and USP28-sgRNA transfected cells were 6.32 µg/mL and 3.97 µg/mL, respectively. **C** Schematic representation of the sgRNAs targeting exon 2 of *USP28* gene. The red arrowheads indicate the positions of sgRNAs target site on the sense DNA strand. PAM sequences are indicated in bold blue font; USP28 sgRNA sequences are indicated in red font. **D** The validation of efficiency of sgRNAs targeting USP28 by transient co-transfection with Cas9 in HEK293 cells and immunoblotting with USP28 antibody. The protein band intensities were estimated using ImageJ software with reference to the GAPDH control (USP28/GAPDH) and presented below the blot. The effect of depleting USP28 on endogenous MAST1 protein was estimated in HEK293 cells. **E** Validation of sgRNA efficiency targeting *USP28* gene by transient co-transfection with Cas9 and sgRNA1 or sgRNA2 into HEK293 cells followed by a T7E1 assay to determine the cleavage efficiency. The cleaved band intensity (indel %) obtained by T7E1 assay was measured using ImageJ software and indicated. Scrambled sgRNA transfected HEK293 cells were used as a control cells. The black arrowhead indicates the cleaved PCR amplicons. A549 cells were transfected with increasing concentrations of **F** Flag-USP28 and **G** Flag-USP28CA to validate its effect on endogenous MAST1 protein levels. **H** The effect of reconstitution of Flag-USP28 on endogenous MAST1 protein in USP28-depleted A549 cells was validated. The protein band intensities for **F**–**H** were estimated using ImageJ software with reference to the GAPDH control band (MAST1/GAPDH) and presented below the blot. HEK293 cells were transfected with constant amount of Myc-MAST1 and increasing concentrations of **I** Flag-USP28 and **J** Flag-USP28CA to validate its effect on exogenous Myc-MAST1 protein levels. **K** The effect of reconstitution of Flag-USP28 on Myc-MAST1 protein in USP28-depleted HEK293 cells was validated. The protein band intensities for **I**–**K** were estimated using ImageJ software with reference to the GAPDH control band (Myc-MAST1/GAPDH) and presented below the blot

To identify factors that regulate MAST1 protein stability, we recently performed an unbiased genome-wide CRISPR/Cas9-based screening for USP subfamily genes and identified USP28 as one of the top potential DUB candidates that may regulate MAST1 protein abundance (Supplementary Fig. 1) [21]. Next, the survival percentage of HeLa cells were measured after treatment with increasing concentration of cisplatin. The IC_50_ value of cisplatin was determined as 6.3 μg/mL for HeLa cells (Supplementary Fig. 2). In order to identify DUBs that sensitize cisplatin resistance, we transfected an entire set of sgRNAs targeting individual USP subfamily genes along with Cas9 nuclease into cisplatin-resistant HeLa cells (HeLa-cisR). Transfected cells were treated with a sub-lethal dose (5 µg/mL) of cisplatin. The cell viability assay results showed that the depletion of USP1 and USP28 increased the cytotoxicity of cisplatin and sensitized the cisplatin-resistance in HeLa cells (Fig. 1A). Furthermore, we cross confirmed the effect of USP28-depletion on sensitizing cisplatin-resistant HeLa cells by treating cells with an increasing concentration of cisplatin. The depletion of USP28 showed a significant decrease in cell viability when compared to the mock control (Fig. 1B).

Previously, we reported that USP1 regulated the MAST1-mediated MEK pathway in cisplatin-resistant cancer cells [21]. In this study, we wish to investigate the role of USP28 on regulating MAST1 protein level and the association with acquisition of cisplatin resistance. To this end, we designed two sgRNAs to target the *USP28* gene at exon 2 (Fig. 1C). The sgRNA2 showed high silencing efficiency in reducing USP28 protein level compared with sgRNA1 (Fig. 1D), which is in line with the high cleavage efficiency exhibited by sgRNA2 in T7E1 assay (Fig. 1E).

### USP28 positively regulates MAST1 protein levels

Next, we wished to investigate the influence of USP28 on MAST1 protein level. To this end, we transfected both A549 and HEK293 cells with increasing concentrations of Flag-USP28 and analyzed MAST1 protein level. We found that increasing concentrations of USP28 stabilized endogenous MAST1 protein levels in a dose-dependent manner (Fig. 1F). However, the overexpression of catalytic mutant of USP28 with a cysteine to alanine mutation at position 171 (USP28C171A) did not exhibit this effect on MAST1 protein (Fig. 1G). We observed a similar USP28-mediated stabilization on Myc-MAST1 in HEK293 cells (Fig. 1I), whereas USP28CA showed no such effect (Fig. 1J). These results indicate that USP28 might act as a protein stabilizer of MAST1 through its deubiquitinating activity. Furthermore, we observed that the sgRNA-mediated reduced expression of MAST1 protein was rescued upon addition of Flag-USP28 in USP28-depleted cells both at endogenous (Fig. 1H, lane 4 vs. lane 2) and exogenous levels (Fig. 1K, lane 5 vs. lane 3). Together, these results suggest that USP28 positively regulates MAST1 protein levels.

### USP28 interacts with and extends the half-life of MAST1

**Fig. 2:**
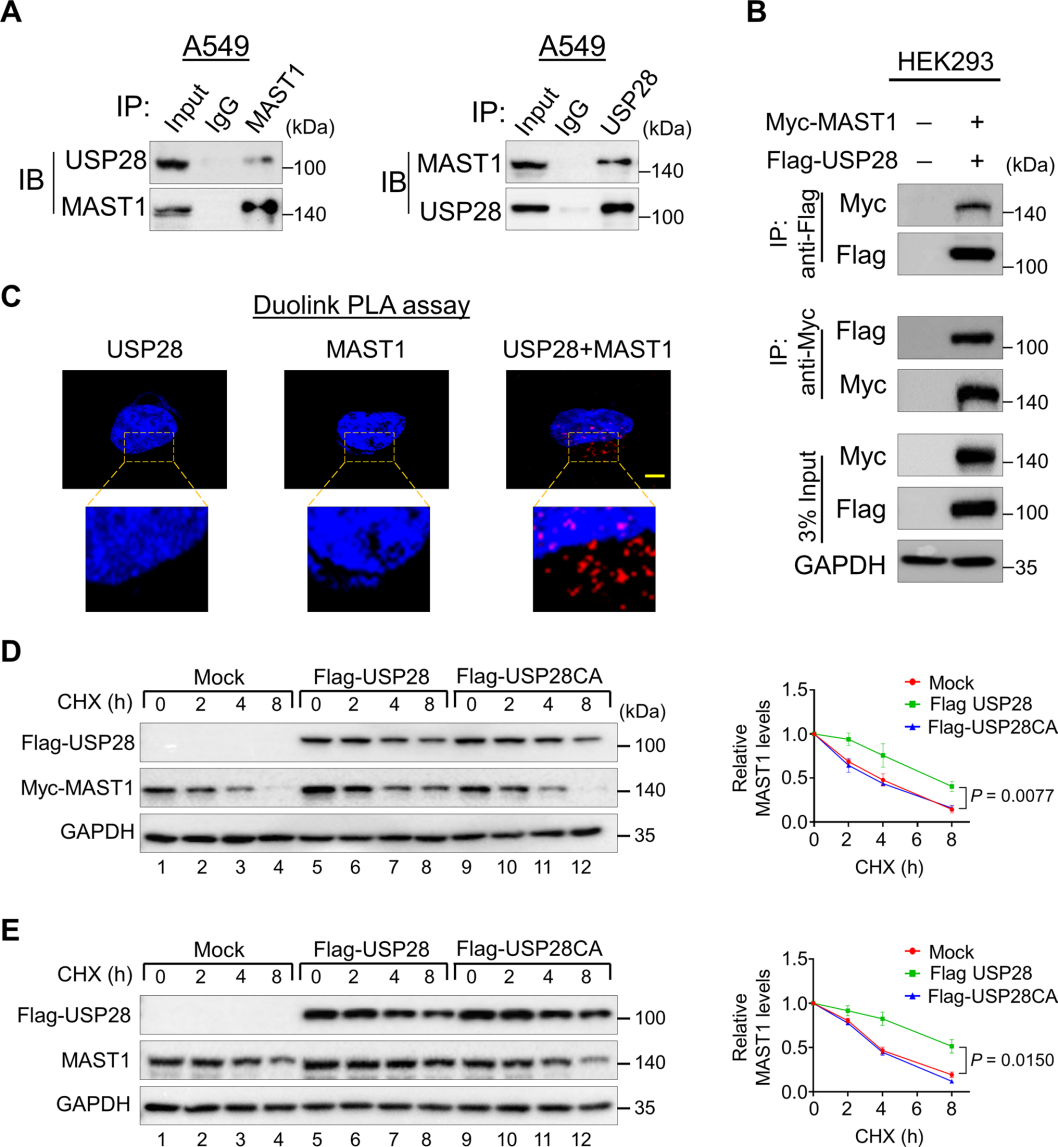
USP28 interacts with MAST1 and extends its half-life. **A** The interaction between endogenous USP28 and MAST1 protein was analyzed in A549 by immunoprecipitation and immunoblotting using the specific antibodies. **B** Interaction between ectopically expressed USP28 and MAST1 was analyzed in HEK293 cells. Cells lysates were immunoprecipitated using Myc or Flag antibodies and analyzed by western blot. GAPDH was used as a loading control. **C** A549 cells were subjected to the Duolink PLA assay to analyze the interaction between USP28 and MAST1 using specific antibodies. The in situ USP28-MAST1 interaction (red PLA dots) was observed when USP28 and MAST1 were immunostained together but not when they were stained with individual antibodies. Scale bar: 10 µm. **D–E** The effect of USP28 and USP28CA on the half-life of Myc-MAST1 in HEK293 (**D**) and endogenous MAST1 (**E**) protein in A549 cells. CHX (150 μg/mL) was administered for the indicated time, and the cells were then harvested for western blotting with the indicated antibodies. The protein band intensities were estimated using ImageJ software with reference to the GAPDH control. Data are presented as the mean and standard deviation of three independent experiments (n = 3). A two-way ANOVA followed by Tukey's post hoc test was used, and *P* values are indicated

To delineate the molecular mechanism by which USP28 regulates MAST1 protein, we first examined the interaction between USP28 and MAST1 under physiological conditions. Co-immunoprecipitation analysis using specific antibodies against endogenous USP28 and MAST1 demonstrated that USP28 interacts with MAST1 and vice versa in A549 cells (Fig. 2A). Co-immunoprecipitation analysis of exogenous Flag-USP28 and Myc-MAST1 in HEK293 cells showed that both proteins interact with each other (Fig. 2B). These findings confirm that USP28 interacts with MAST1 protein at both endogenous and exogenous levels. We further used Duolink PLA assays to validate the interaction between USP28 and MAST1 under physiological conditions. The in situ USP28-MAST1 interaction was observed as red signals (PLA dots) when USP28 and MAST1 were immunostained together, but not when they were stained with individual antibodies (Fig. 2C).

On the basis of the above observations, we hypothesized that the interaction between USP28 and MAST1 might influence on MAST1 protein turnover. To this end, we treated A549 cells with the protein synthesis inhibitor cycloheximide (CHX) in the presence or absence of USP28. We observed an extended half-life of MAST1 exogenous (Fig. 2D, lane 5–8) as well as endogenous protein (Fig. 2E, lane 5–8) in the presence of USP28 compared with the mock group. However, there was no effect of USP28 catalytic mutant on the half-life of MAST1 protein (Fig. 2D and 2E, lane 9–12), suggesting that the deubiquitinating activity of USP28 regulates MAST1 protein turnover.

### USP28 deubiquitinates MAST1

**Fig. 3:**
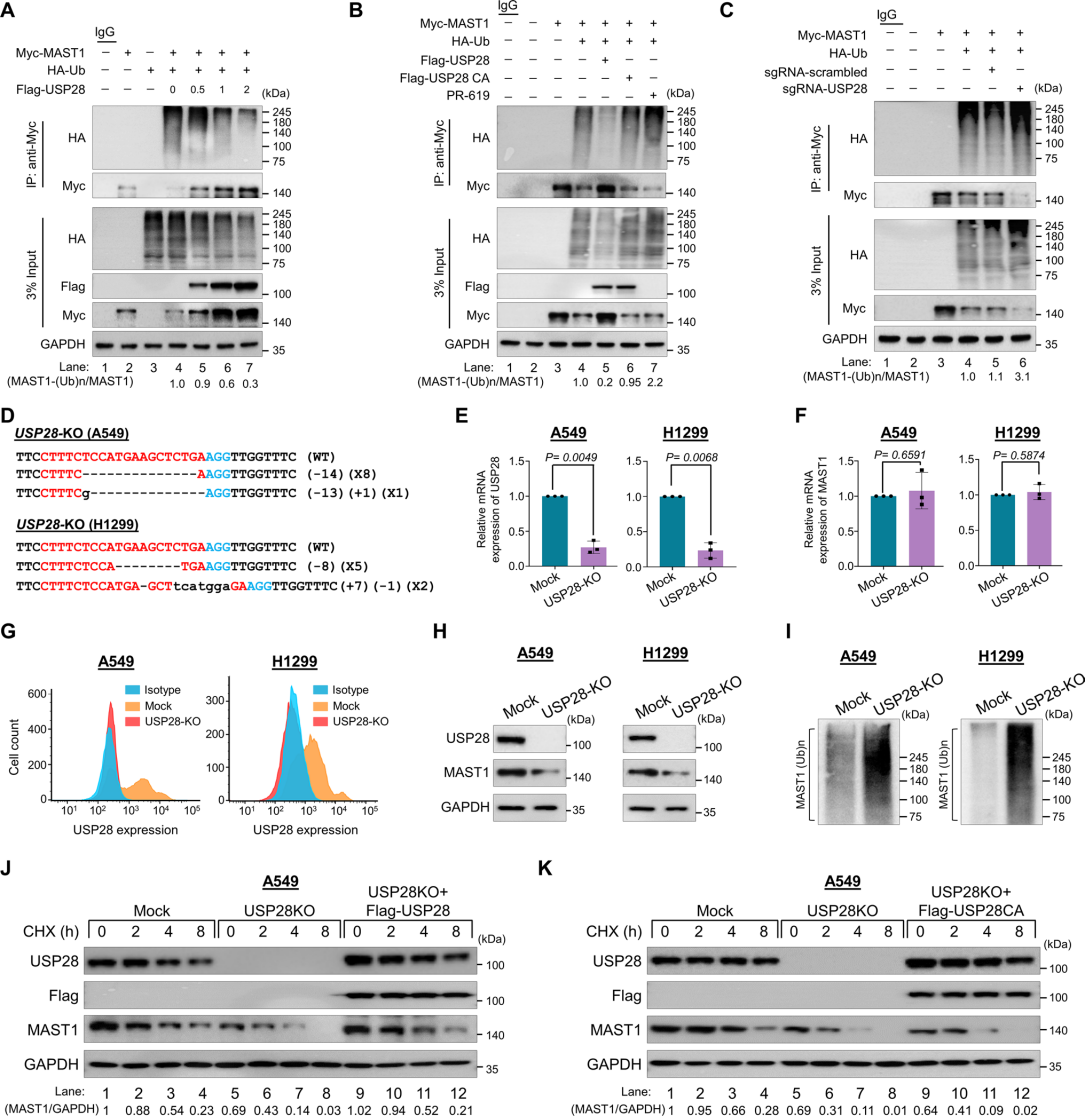
USP28 deubiquitinates MAST1 protein. The ubiquitination and deubiquitination of ectopically expressed Myc-MAST1 were analyzed in HEK293 cells. **A** The HEK293 cells were transfected with Myc-MAST1 and HA-Ub in a constant amount. Flag-USP28 was transfected in an increasing concentration, followed by immunoprecipitation with Myc antibody and immunoblotting with anti-HA antibody. **B** The ubiquitination and deubiquitination of ectopically expressed Myc-MAST1 were analyzed by transfecting HEK293 cells with Flag-USP28 and Flag-USP28CA or treatment with DUB-inhibitor PR-619 for 48 h prior to harvest in the HEK293 cells. The cells were harvested, followed by IP with a Myc antibody and immunoblotting with an anti-HA antibody. **C** The ubiquitination and deubiquitination of ectopically expressed Myc-MAST1 were analyzed by transfecting HEK293 cells with sgRNAs targeting USP28. The cells were harvested, followed by IP with a Myc antibody and immunoblotting with an anti-HA antibody. **A**–**C** The relative protein expression of MAST1-(Ub)n with respect to input MAST1 was quantified using ImageJ software and represented as (MAST1-(Ub)n/MAST1) below the blot. **D** Sanger sequencing data showing the disruption in *USP28* gene sequence in A549 (upper panel) and H1299 cells (lower panel). The effect of USP28-KO on the mRNA expression of **E** *USP28* and **F** *MAST1* was evaluated by qRT-PCR with specific primers. The relative mRNA expression levels are shown after normalization to GAPDH mRNA expression. Data are presented as the mean and standard deviation of three independent experiments (n = 3). A two tailed t-test was used, and *P* values are indicated. **G** Flow cytometry assay showing the expression of USP28 in mock control vs. USP28-KO in A549 cells (left panel) and H1299 cells (right panel). **H** Western blot analysis of the endogenous expression of USP28 and MAST1 protein in A549 and H1299 USP28-KO was evaluated. GAPDH was used as the internal loading control. **I** The TUBEs assay was performed to assess the ubiquitination status of the MAST1 protein in mock control and USP28-KO clones from A549 and H1299 cells. Cell lysates were immunoprecipitated with TUBEs beads, followed by immunoblotting with the indicated antibodies. **J–K** The effect of USP28-KO on the half-life of MAST1 in A549 cells. The mock control, USP28-KO and USP28-KO cells reconstituted with (**J**) Flag-USP28 and **K** Flag-USP28CA was treated with CHX (150 μg/mL) for the indicated time, and the cells were then harvested for western blotting with the indicated antibodies. The protein band intensities were estimated using ImageJ software with reference to the GAPDH control

To analyze the effect of deubiquitinating activity of USP28 on MAST1 polyubiquitination, we performed deubiquitination assays by transfecting increasing concentrations of USP28 plasmid. The high-molecular-weight polyubiquitin smear of MAST1 protein was reduced in the presence of USP28 in a dose-dependent manner (Fig. 3A). However, the catalytically mutant USP28 did not show any deubiquitinating activity on MAST1 protein (Fig. 3B, lane 6 vs. lane 5). In contrast, the DUB inhibitor (PR-619) increased the ubiquitin smear on MAST1 protein (Fig. 3B, lane 7). Likewise, knockdown of USP28 resulted in an increase in the polyubiquitination smear of MAST1 compared with mock (Fig. 3C, lane 6 vs. lane 5).

To further validate the effect of USP28-mediated post-translational regulation of MAST1, we generated single-cell derived *USP28-*knockout clones in both A549 and H1299 cell lines using the CRISPR/Cas9 system. We used sgRNA2 targeting exon 2 of the *USP28* gene co-transfected with Cas9 and subjected cells to puromycin selection for 2 days. The selected cells were then diluted into 96-well plates for single-cell clonal selection. As a mock control, A549 and H1299 cells transfected with scrambled sgRNA were also subjected to single cell dilution. Single cell–derived knockout clones were then screened for *USP28* gene disruption using T7E1 assays. The T7E1-positive single cell–derived USP28 knockout clones (Supplementary Fig. 3A and C) were subjected to western blotting to further confirm the *USP28* disruption. Western blot analysis revealed complete disruption of USP28 protein expression in clones #12 in A549 cells (Supplementary Fig. 3B) and clones #3 and #7 in H1299 cells (Supplementary Fig. 3D). Moreover, Sanger sequencing results revealed out-of-frame mutations in clone #12 from A549 cells (Fig. 3D, upper panel) and clone #3 from H1299 cells (Fig. 3D, lower panel) (hereafter referred to as USP28-KO).

Next, we analyzed the effect of *USP28* knockout on the mRNA and protein levels of MAST1 by qPCR, western blotting and FACS analysis. USP28-KO clones in both A549 and H1299 cells displayed complete disruption of USP28 expression at both mRNA (Fig. 3E) and protein (Fig. 3G, H) levels and subsequently reduced MAST1 protein level (Fig. 3H). However, the loss of USP28 did not exert significant changes on MAST1 mRNA level (Fig. 3F), indicating that USP28 does not have transcriptional control over MAST1. To further support our results, we analyzed the ubiquitination pattern of MAST1 protein in USP28-KO clones from A459 and H1299 cells. We performed TUBEs assay, which has a high-affinity probe for ubiquitinated proteins [29]. Our data showed a high ubiquitin smear on MAST1 protein in USP28-KO clone obtained from both A459 and H1299 cells compared with the mock control (Fig. 3I), indicating enhanced polyubiquitination of MAST1 in the absence of USP28. Furthermore, we checked the effect of USP28 depletion on half-life of MAST1 protein in both A549 cells and H1299 cells. The half-life of MAST1 was drastically reduced in USP28-KO cell lines (Fig. 3J, K, lane 5–8; Supplementary Fig. 4A, B, lane 5–8). However, the half-life of MAST1 protein was rescued when USP28-KO cells were reconstituted with Flag-USP28 (Fig. 3J, lane 9–12; Supplementary Fig. 4A, lane 9–12), while USP28CA failed to increase the half-life of MAST1 protein (Fig. 3K, lane 9–12; Supplementary Fig. 4B, lane 9–12). Thus, these results showed that USP28 regulates MAST1 expression at the post-translational level via its deubiquitinating activity.

### USP28 and MAST1 expression analysis in a wide range of cancer types

**Fig. 4:**
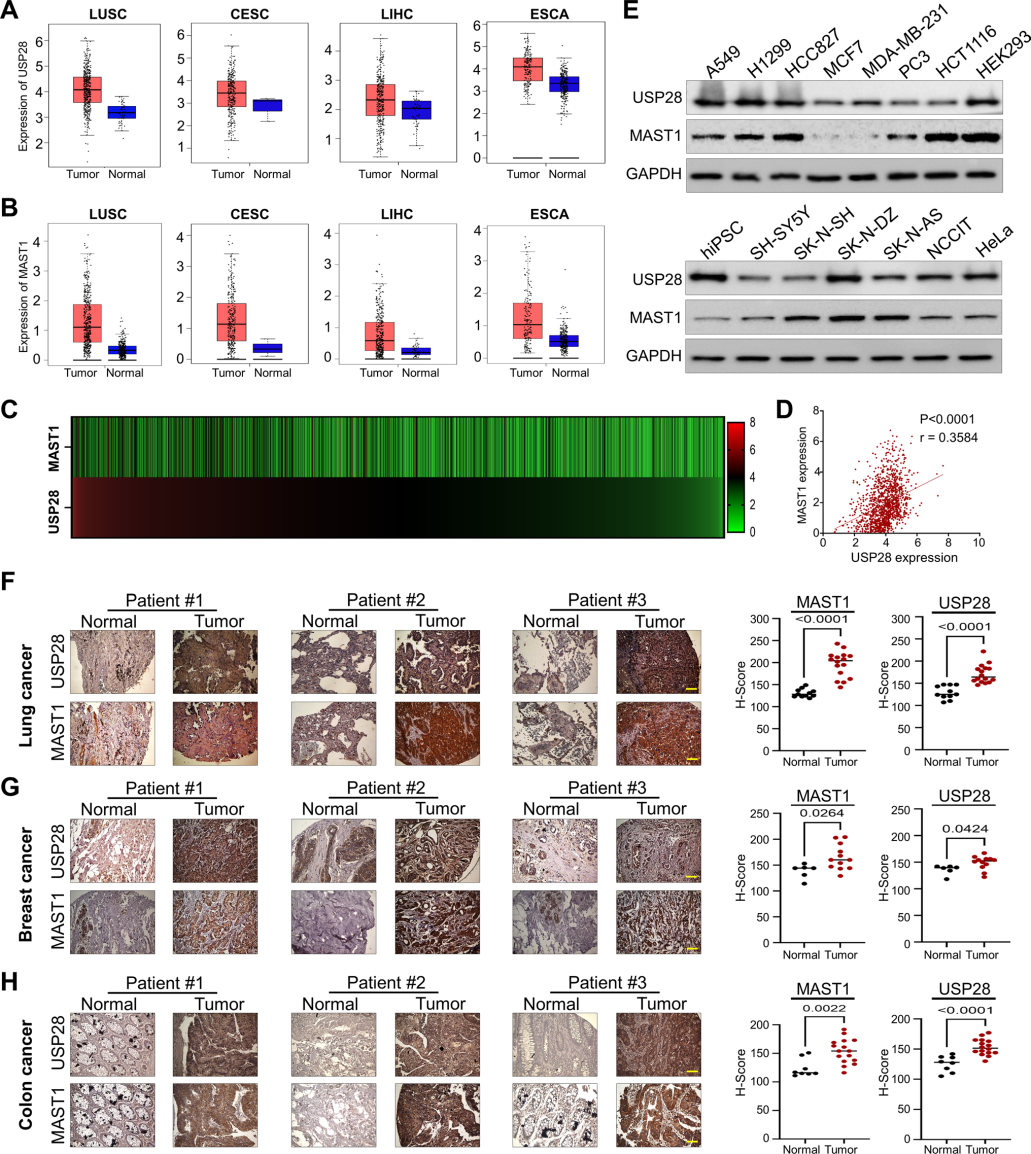
Correlation between USP28 and MAST1 expression in various cancers tissues. **A** Box plot showing difference between USP28 expression in tumor and normal tissues in LUSC, CESC, LIHC and ESCA cancer types. **B** Box plot showing difference between MAST1 expression in tumor and normal tissues in LUSC, CESC, LIHC and ESCA cancer types. The box plots (A-B) were generated using online bioinformatics tool GEPIA 2 (http://gepia2.cancer-pku.cn/#index). **C** A heat map showing mRNA expression levels of USP28 and MAST1 in different cancer cell lines derived from the CCLE database. Representative samples are arranged from high to low mRNA levels of USP28, and corresponding MAST1 values are sorted. **D** A scatterplot showing the expression correlation between USP28 and MAST1 mRNA levels in different cancer cell lines derived from the CCLE database. Pearson correlations (r) quantifying the relationship between USP28 and MAST1 are given. **E** Endogenous protein expression patterns of USP28 and MAST1 in different cancer and non-cancer cell lines were assessed by Western blotting. GAPDH was used as the loading control. **F–H** Representative immunohistochemical (IHC) staining images of endogenous USP28 and MAST1 in **F** human lung cancer (n = 27), **G** breast cancer (n = 18) and **H** colon cancer (n = 24) tissues. All IHC images were quantified with an H-score and difference in expression of MAST1 and USP28 in normal and tumor samples was represented graphically. Scale bar = 30 µm

We used the TCGA database to evaluate the normal-tumor matched mRNA expression level of USP28 and MAST1 in different cancer types. USP28 was upregulated in 62% of cancer types (15 of 24) (Fig. 4A; Supplementary Fig. 5). MAST1 was also upregulated in 54% of cancer types (13 of 24) (Fig. 4B; Supplementary Fig. 6). USP28 and MAST1 was significantly up-regulated in LUSC, LUAD, COAD, BRCA, CESC, DLBC, ESCA, HNSC, KICH, LIHC, OV, PAAD, and THYM compared with normal control (Fig. 4A, B; Supplementary Fig. 5 and 6), suggesting that high USP28 and MAST1 mRNA expression level might be associated with tumorigenesis. We further analyzed the expression of USP28 and MAST1 in a wide range of cancer cell lines using the Cancer Cell Line Encyclopedia (CCLE) database. The high mRNA expression score of USP28 was proportional with MAST1 expression (Fig. 4C; Supplementary Table S5) and showed a positive correlation between USP28-MAST1 exhibiting r value of 0.36 (Fig. 4D).

Furthermore, the expression level of USP28 corresponded with MAST1 protein in several tested cancer cell lines (Fig. 4E). Additionally, we demonstrated the clinical relevance of USP28 and MAST1 expression in human clinical tissue samples by immunohistochemistry staining. High expression of USP28 and MAST1 was observed in lung cancer (Fig. 4F), breast cancer (Fig. 4G) and colon cancer samples (Fig. 4H). Non-parametric spearmen correlation revealed a positive correlation between USP28 and MAST1 expression in lung cancer (Spearman’s rho = 0.81); breast cancer (Spearman’s rho = 0.61) and colon cancer (Spearman’s rho = 0.76) (Supplementary Fig. 7 A–C).

### Abrogation of USP28 induces DNA damage and cell death

**Fig. 5:**
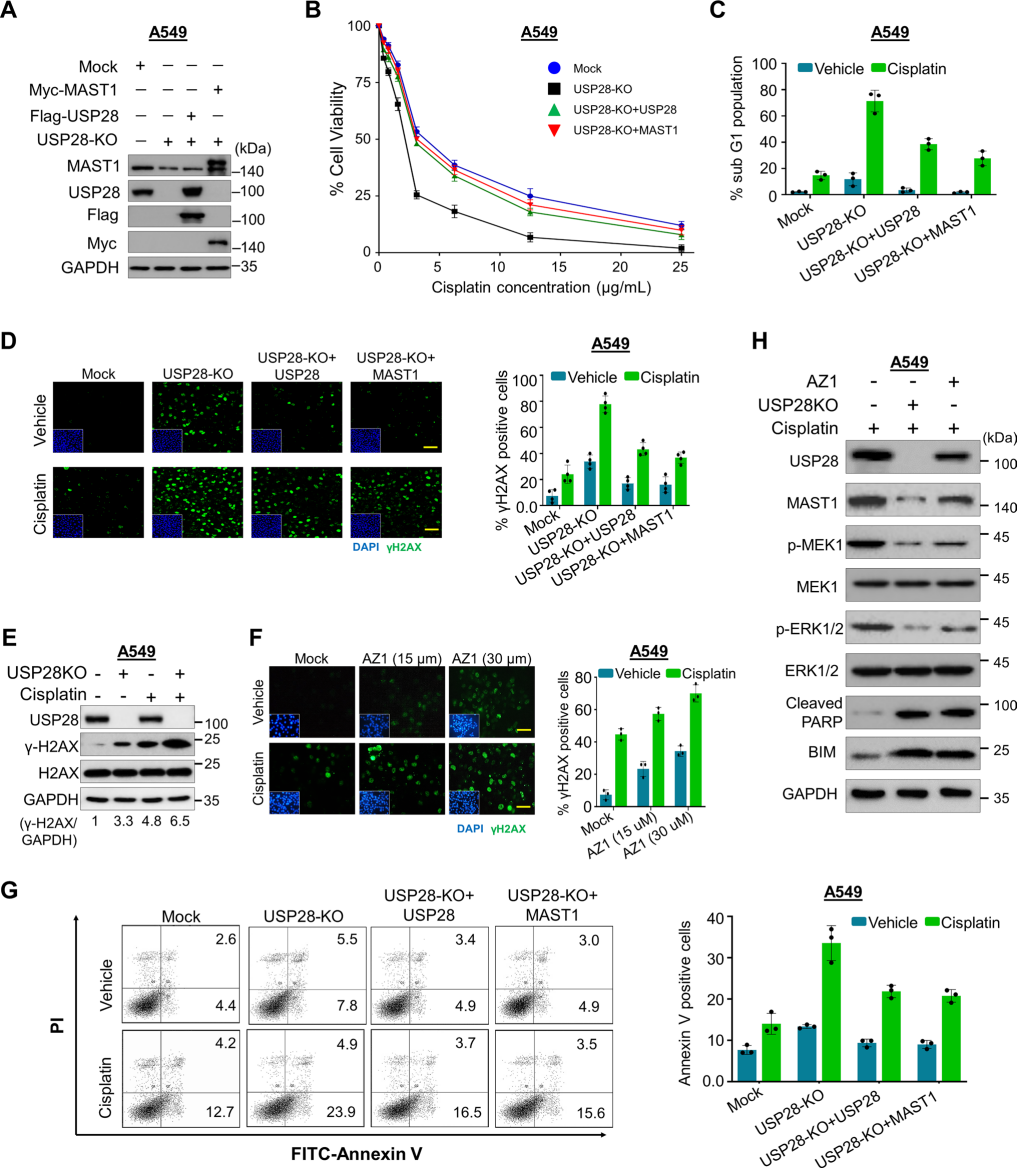
Loss of USP28 suppresses cell viability and promotes DNA damage and apoptosis. Mock control, USP28-KO, and USP28-KO cells reconstituted with either USP28 or MAST1 were used to perform the following experiments. **A** Western blot analysis to validate the expression of USP28 and MAST1 using USP28- and MAST1-specific antibodies in A549 cells. The cells from **A** were subjected to the following experiments. **B** A549 cells were treated with an increasing concentration of cisplatin (5 µg/mL, 10 µg/mL, 15 µg/mL, 20 µg/mL and 25 µg/mL) for 48 h, and cell viability was assayed using CCK-8 reagent. Data are presented as the mean and standard deviation of three independent experiments (n = 3). The IC_50_ values of cisplatin in A549 mock, USP28-KO, and USP28-KO reconstituted with USP28 and USP28-KO reconstituted with MAST1 were 3.56 µg/mL, 2.13 µg/mL, 3.36 µg/mL, and 3.44 µg/mL, respectively. **C** A549 cells were treated with cisplatin (2 µg/mL) for 48 h and subjected to flow cytometry to measure the DNA content using PI staining and Data are presented as the mean and standard deviation of three independent experiments (n = 3). **D** A549 cells were treated with either vehicle or Cisplatin (2 µg/mL) for 48 h and subjected to immunofluorescence analysis to estimate γH2AX foci formation. Green, γH2AX; blue, nucleus stained by DAPI. Scale bar = 100 µm. The right panel depicts the percentage of γH2AX-positive cells. Data are presented as the mean and standard deviation of three independent experiments (n = 3). **E** A549 cells treated with cisplatin (2 µg/mL) for 48 h were subjected to immunoblotting analysis with the indicated antibodies. The protein band intensities were estimated using ImageJ software with reference to the GAPDH control (γ-H2AX/GAPDH) and presented below the blot.** F** A549 cells were treated with the indicated concentrations of USP28 inhibitor (AZ1) with either vehicle or cisplatin (2 µg/mL) for 48 h and subjected to immunofluorescence analysis to estimate γH2AX foci formation. Green, γH2AX; blue, nucleus stained by DAPI. Scale bar = 100 µm. The right panel depicts the percentage of γH2AX-positive cells. Data are presented as the mean and standard deviation of three independent experiments (n = 3). **G** A549 cells were treated with cisplatin (2 µg/mL) for 48 h. Flow cytometry analysis was performed to analyze annexin-V and PI positive cells and graphically represented. Data are presented as the means and standard deviations of 3 independent experiments. **H** The effect of USP28 depletion or USP28 inhibitor (AZ1) on MEK pathway in A549 cells treated with cisplatin (2 µg/mL) by western blotting with indicated antibodies. GAPDH was used as the internal loading control

We next investigated the effect of loss of USP28 on MAST1-mediated cisplatin resistance in both A549 and H1299 cells. We used USP28-KO clones exhibiting reduced MAST1 protein level and the cells were reconstituted either with Flag-USP28 or Myc-MAST1 for further functional experiments (Fig. 5A; Supplementary Fig. 8). First, we estimated the relative cell survival of A549 and H1299 cells with exposure to an increasing concentration of cisplatin (Supplementary Fig. 9A, B). We next used USP28-KO cells to validate the USP28 dependence for cisplatin cytotoxicity by treating cells with increasing concentration of cisplatin. The loss of USP28 resulted in lower cell viability compared with the mock control, while reconstitution with Flag-USP28 or Myc-MAST1 increased cell viability (Fig. 5B; Supplementary Fig. 10). Furthermore, USP28-depleted cells showed an increase in sub-G1 populations whereas reconstitution with Flag-USP28 or Myc-MAST1 decreased sub-G1 populations (Fig. 5C; Supplementary Fig. 11). Next, we estimated the extent of DNA damage caused by cisplatin in the presence or absence of USP28 by assessing γH2AX expression. USP28-KO clones from both A549 and H1299 cells showed increased γH2AX foci formation compared with mock control, while reconstitution with Flag-USP28 or MAST1 reduced γH2AX foci formation (Fig. 5D; Supplementary Fig. 12A). Furthermore, we verified via western blotting that USP28-KO clone treated with cisplatin had higher expression of γH2AX than mock control cells treated with cisplatin (Fig. 5E; Supplementary Fig. 12B). Additionally, treatment with USP28 inhibitor (AZ1) along with cisplatin on A549 or H1299 cells showed an increase in γH2AX foci formation when compared with control (Fig. 5F; Supplementary Fig. 13). Likewise, Annexin-V-positive cells were increased in USP28-KO clones compared with USP28-depleted cells reconstituted with USP28 or MAST1 (Fig. 5G), indicating that loss of USP28 sensitizes cells to cisplatin-mediated DNA damage and induced cell death.

Finally, we wished to investigate the molecular mechanism of USP28 regulation on MAST1-driven cisplatin-resistance. To this end, we examined MAST1-mediated MEK1 activation and subsequent ERK1/2 phosphorylation in USP28-KO cells treated with cisplatin. Interestingly, USP28-KO cells treated with cisplatin displayed reduced expression of phosphorylated MEK1 and ERK, suggesting that USP28 regulates MAST1-mediated MEK pathway (Fig. 5H; Supplementary Fig. 14). Furthermore, cisplatin or AZ1 treated cells showed an increase in expression of apoptotic markers BIM and cleaved PARP (Fig. 5H; Supplementary Fig. 14).

### Loss of USP28 inhibits MAST1-mediated tumor progression

**Fig. 6:**
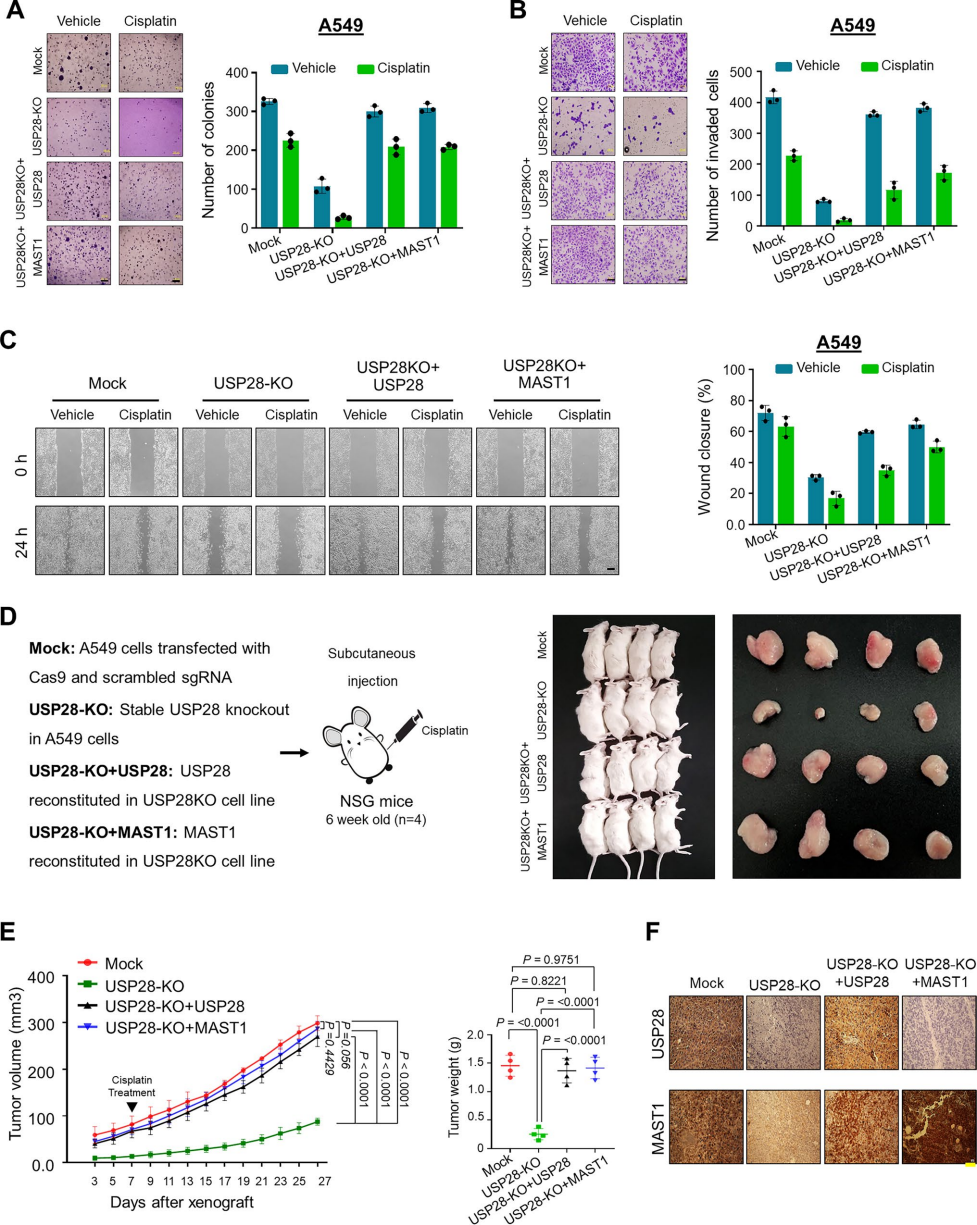
Loss of US28 inhibits tumorigenesis in vitro and in vivo. Mock control, USP28-KO, and USP28-KO cells reconstituted with either USP28 or MAST1 were used to perform the following experiments. The cells from (5A) were treated with either vehicle or cisplatin and were subjected to the following experiments**. A** Colony formation was measured after 14 days in A549 cells**.** The colony numbers were quantified and are presented graphically. Scale bar, 500 µm. **B** The transwell cell invasion assay was performed with the groups mentioned in A549 cells. The number of invaded cells were quantified using ImageJ software and represented graphically. Data are presented as the means and standard deviations of 3 independent experiments. Scale bar, 100 µm. **C** The migration potential of above mentioned groups was assessed by an in vitro scratch assay in A549 cells. The migration potential was quantified by ImageJ software and are presented graphically. Scale bar, 200 µm. Data are presented as the means and standard deviations of 3 independent experiments. **D** Xenografts were generated by subcutaneously injecting the mentioned cell groups into the right flanks of NSG mice (n = 4/group). Mice were i.p. injected with either saline (vehicle) or cisplatin (2 mg/kg) twice a week beginning 7 days after xenograft implantation, and tumor size was monitored. Tumor volumes were recorded, and tissues were stored for IHC experiments. The right panel shows the tumors excised from the mice after the experiment. **E** Tumor volume and tumor weight were measured and are presented graphically. Data are presented as the mean and standard deviation (n = 4 mice per group). Statistical power is 80% and was calculated post experiment using G*Power software. Two-way ANOVA followed by Tukey's post-hoc test was used, and the exact *P* values are indicated on the figures (*P* < 0.05,* P* < 0.01, *P* < 0.001,* P* < 0.0001 were considered as significant, and *P* > 0.05 considered as non-significant). **F** Xenograft tumors were embedded in paraffin and sectioned. IHC analyses were performed with the indicated antibodies. Scale bar = 30 µm

To determine whether USP28 expression contributes to MAST1-mediated tumor growth, USP28-KO clones from both A549 and H1299 cells were treated with cisplatin and subjected to several in vitro carcinogenesis experiments. An anchorage-independent colony formation assay demonstrated that USP28-KO clones showed reduced colony numbers compared with the mock control, while the colony-forming ability was increased in USP28-depleted cells reconstituted with USP28 or MAST1 (Fig. 6A and Supplementary Fig. 15A). Similarly, cellular invasion and migration rate were significantly hindered in USP28-KO clones, whereas USP28-depleted cells reconstituted with USP28 or MAST1 showed reversed results (Fig. 6B, C and Supplementary Fig. 15B, C).

Next, we measured the ability of USP28-depleted cells to impair tumor growth in NSG mice by subcutaneously injecting USP28-KO cells and USP28-depleted cells reconstituted with either USP28 or MAST1 obtained from Fig. 5A. The mice bearing USP28-KO cells treated with cisplatin showed reduced tumor size, volume and weight compared with the mock control group (Fig. 6D, E, Supplementary Table S6). However, mice bearing USP28-depleted cells reconstituted with USP28 or MAST1 displayed increased tumor size, volume and weight (Fig. 6D, E, Supplementary Table S6). Additionally, immunohistochemical staining of xenograft tumor tissues revealed reduced USP28 and MAST1 expression in the USP28-KO tumors compared with the mock control xenograft tumor tissues, while the expressions of USP28 and MAST1 were regained by reconstitution with USP28 or MAST1 (Fig. 6F). Together, these results indicate that loss of USP28 decreased MAST1 protein level and suppressed tumor growth upon cisplatin treatment.

## Discussion

Anti-cancer drug resistance is a major problem in cancer treatment, leading to treatment failure in patients receiving chemotherapy. Platinum-based chemotherapy, especially cisplatin, is widely used to treat variety of cancer types [2, 3, 33]. Cisplatin triggers apoptotic signaling in cancers cells by crosslinking with DNA, which subsequently interrupts DNA synthesis and repair mechanisms [5]. Cisplatin treatment leads to a high level of drug resistance in patients and recurrence of tumors with cisplatin resistance is frequently observed. Several factors contribute to acquired resistance such as increased drug efflux, activation or inactivation of pro-apoptotic cellular signaling and DNA-adduct repair [5, 34, 35]. Thus, identifying the mechanism of acquired resistance to cisplatin treatment is critical.

Several researchers have identified factors involved in the mechanism of acquired resistance after repeated cisplatin treatment. MAST1 emerged as a critical driver of cisplatin resistance in several tumors [18]. Later studies showed that MAST1 protein ubiquitination and stabilization is regulated by the E3 ligase CHIP and molecular chaperone hsp90, respectively [19]. Thus, the regulation of MAST1 protein level was considered as a critical factor for cisplatin resistance in cancer types. We recently performed CRISPR/Cas9-based genome-wide screening for DUBs that regulate MAST1 protein levels as well as DUBs that confer cisplatin resistance in cancers [21]. Our screening system identified USP28 as one of the potential DUBs regulating cisplatin resistance in cancers. Depletion of USP28 destabilized MAST1 protein levels and also sensitized cisplatin-resistant cancer cells for cisplatin-mediated cytotoxicity. In line with our results, inhibition of USP28 destabilized ΔNp63 protein and sensitize squamous cell carcinomas cells for cisplatin treatment [25].

In this study, we demonstrated that USP28 stabilizes MAST1 protein by preventing its protein degradation and subsequently extends MAST1 protein half-life. We also demonstrated that depletion of USP28 enhances cisplatin-induced DNA damage and cell death, which was evident by high γH2AX foci formation and Annexin-V positive cells. USP28 plays an important role in the DNA damage response. USP28 deubiquitinates and stabilizes factors in ATM and ATR signaling by preventing their protein degradation. These factors, including 53BP1, Claspin and MDC1, associate with USP28 and are critical in controlling the DNA damage response [24]. USP28 is also an ATM substrate in response to DNA damage and regulates CHK2-dependent apoptosis. USP28 stabilizes claspin protein, a key regulator of CHK1 activity, and maintains G2 arrest, suggesting the role of USP28 in regulating DNA damage response factors during DNA repair [36]. Likewise, we demonstrated that the depletion of USP28 can hinder cell proliferation, wound healing, invasion and colony-formation ability. Furthermore, we showed that the loss of USP28 downregulates MAST1 protein level and sensitizes cells for cisplatin toxicity during tumor growth, leading to reduced tumor size, volume and weight.

In conclusion, our study demonstrates that USP28 could enhance MAST1-driven cisplatin resistance by stabilizing MAST1 protein level in cancer. Thus, USP28 may be a potential therapeutic target along with MAST1 to synergistically boost the effect of cisplatin-based treatment to overcome cisplatin resistance in cancer patients.

### Supplementary Information

Below is the link to the electronic supplementary material.

Supplementary file1 (DOCX 5512 KB)

## Data Availability

The datasets analyzed during the current study are available from the corresponding author on reasonable request.
