# Supplementary material for: USP28 promotes tumorigenesis and cisplatin resistance by deubiquitinating MAST1 protein in cancer cells
Source: Cell Mol Life Sci. 2024 Mar 18;81(1):145. doi: 10.1007/s00018-024-05187-2 (PMC10948558; doi:10.1007/s00018-024-05187-2)
Supplement: Supplementary file 1 — Supplementary file1 (DOCX 5512 KB) [file 18_2024_5187_MOESM1_ESM.docx]

**USP28 promotes tumorigenesis and cisplatin resistance by deubiquitinating MAST1 protein in cancer cells**

Janardhan Keshav Karapurkar^1,^ **^†^**, Jencia Carminha Colaco^1^**^†^**, Bharathi Suresh^1^**^†^**, Apoorvi Tyagi^1^, Sang Hyeon Woo^1^, Won-Jun Jo^1^, Nare Ko^2,3^, Vijai Singh^4^, Seok-Ho Hong^5^, Seung Jun Oh^3^*****, Kye-Seong Kim^1,6^***** and Suresh Ramakrishna^1,6^*****

^1^Graduate School of Biomedical Science and Engineering, Hanyang University, Seoul, 04763, South Korea

^2^Biomedical Research Center, Asan Institute for Life Sciences, Seoul 05505, Korea.

^3^Department of Nuclear Medicine, Asan Medical Center, University of Ulsan College of Medicine, Seoul 05505, Korea.
^4^Department of Biosciences, School of Science, Indrashil University, Rajpur, Mehsana, Gujarat, India.

^5^Department of Internal Medicine, School of Medicine, Kangwon National University, Chuncheon, South Korea

^6^College of Medicine, Hanyang University, Seoul, 04763, South Korea

**Running title:** Loss of USP28 sensitizes cancer cells to cisplatin treatment.

**^†^**These authors contributed equally.

***Corresponding Authors Information**

Suresh Ramakrishna (E-mail: [suri28@hanyang.ac.kr](mailto:suri28@hanyang.ac.kr), [suresh.ramakris@gmail.com](mailto:suresh.ramakris@gmail.com))

Kye-Seong Kim (E-mail: ks66kim@hanyang.ac.kr)

Seung Jun Oh (E-mail: sjoh@amc.seoul.kr)

**Supplementary Figures:**

**Supplementary Figure 1.** DUB knockout library based screening for DUBs regulating MAST1 protein levels.

**Supplementary Figure 2.** In vitro dose response curve of cisplatin in HeLa-CisR cell line.

**Supplementary Figure 3.** Screening for USP28 knockout clones in A549 and H1299 cells.

**Supplementary Figure 4.** USP28 extends the half-life of MAST1 in H1299 cells.

**Supplementary Figure 5.** The mRNA expression profile of USP28 in various cancers and normal tissues.

**Supplementary Figure 6.** The mRNA expression profile of MAST1 in various cancers and normal tissues.

**Supplementary Figure 7.** Non-parametric spearmen correlation test for IHC staining of cancer tissues.

**Supplementary Figure 8.** Western blot analysis to validate the expression of endogenous USP28 and MAST1 in USP28-KO H1299 cells.

**Supplementary Figure 9.** In vitro dose response curve of cisplatin in A549 and H1299 cell lines.

**Supplementary Figure 10.** Cell viability assay in H1299 cells treated with increasing concentration of cisplatin

**Supplementary Figure 11.** Estimating sub-G1 population in H1299 cells by flow cytometry**.**

**Supplementary Figure 12.** Loss of USP28 promotes DNA damage in H1299 cells.

**Supplementary Figure 13.** γH2AX foci formation in H1299 cells treated with USP28 inhibitor (AZ1) by immunofluorescence analysis.

**Supplementary Figure 14.** The regulation of USP28 on MAST1-mediated MEK1 activation in H1299 cells.

**Supplementary Figure 15.** Loss of USP28 inhibits tumorigenesis in H1299 cells.

**Supplementary Tables**

**Supplementary Table S1**. Target sequences used for sgRNA plasmid construction.

**Supplementary Table S2.** Oligonucleotide sequences used to get PCR amplicon for T7E1 assay.

**Supplementary Table S3.** PCR amplicon and cleavage sizes after T7E1 assay.

**Supplementary Table S4.** Oligonucleotide sequence used for qRT-PCR.

**Supplementary Table S5.** The mRNA scores for *USP28* and *MAST1* expression derived from the Cancer Cell Line Encyclopedia database in various cancer cell lines.

**Supplementary Table S6.** Tumor weight and volume measured for animal study.


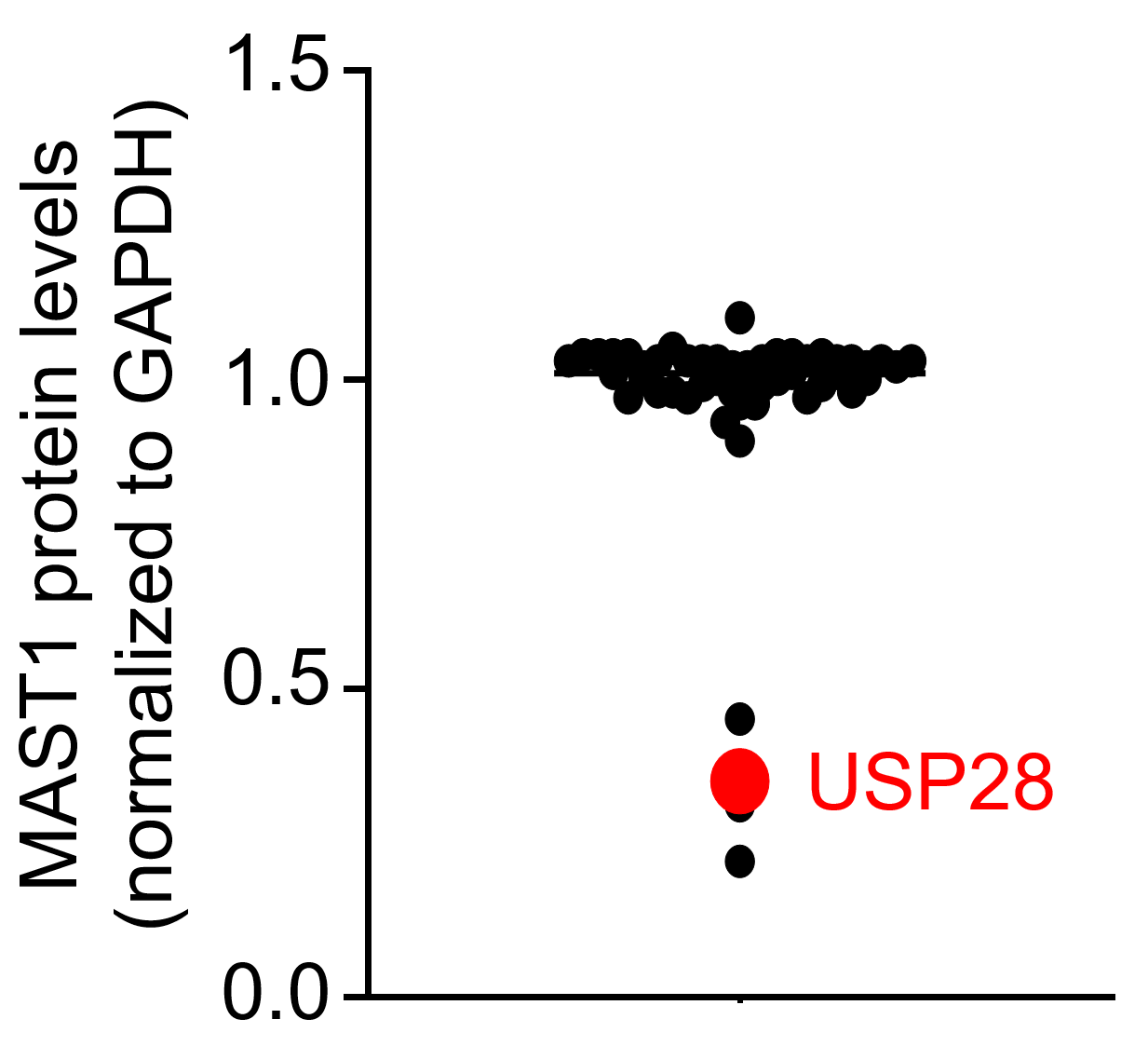


**Supplementary Figure 1.** DUB knockout library based screening for DUBs regulating MAST1 protein levels. Equal protein concentration of individual DUB knockout cell lysates (USP1 to USP50, represented by black dots) were subjected to western blot analysis to determine the effect of DUB knockout on MAST1 protein levels as previously reported (21). The depletion of USP1, USP9, USP28 and USP44 showed low MAST1 protein level. USP28 is represented by a red dot which is the protein of interest in this study. The protein band intensities were estimated using ImageJ software with reference to the GAPDH control for each individual sgRNA and represented in the graph above.

**
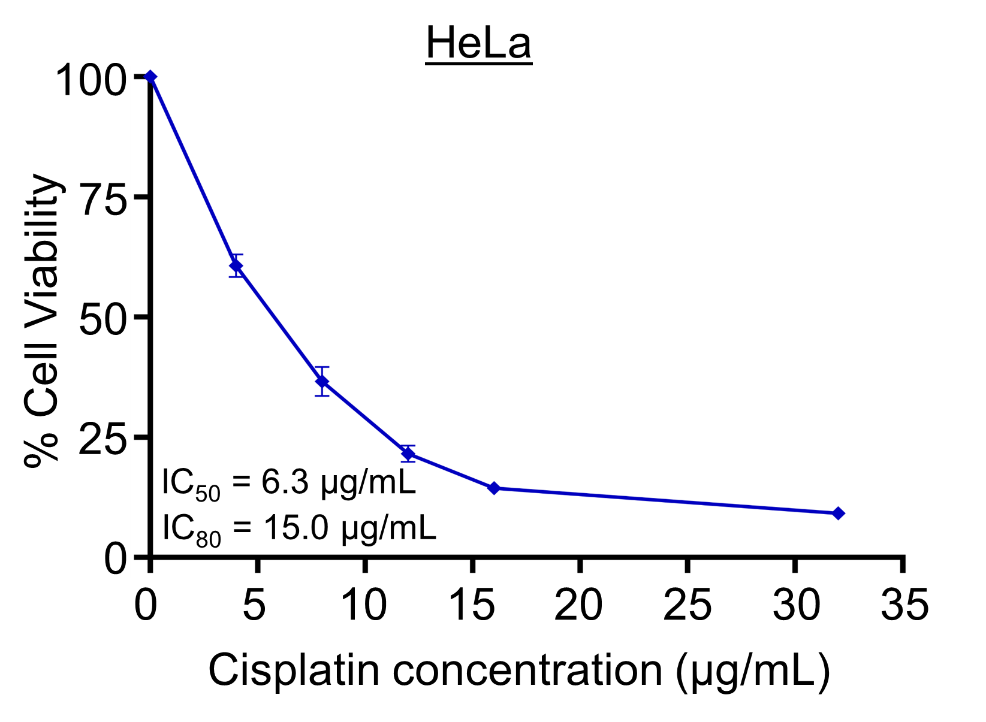
**

**Supplementary Figure 2.** In vitro dose response curve of cisplatin in HeLa-CisR cell line. HeLa cells were treated with increasing concentration of cisplatin and cell viability was assessed by CCK-8 kit. Data are presented as the means and standard deviations of 3 independent experiments.


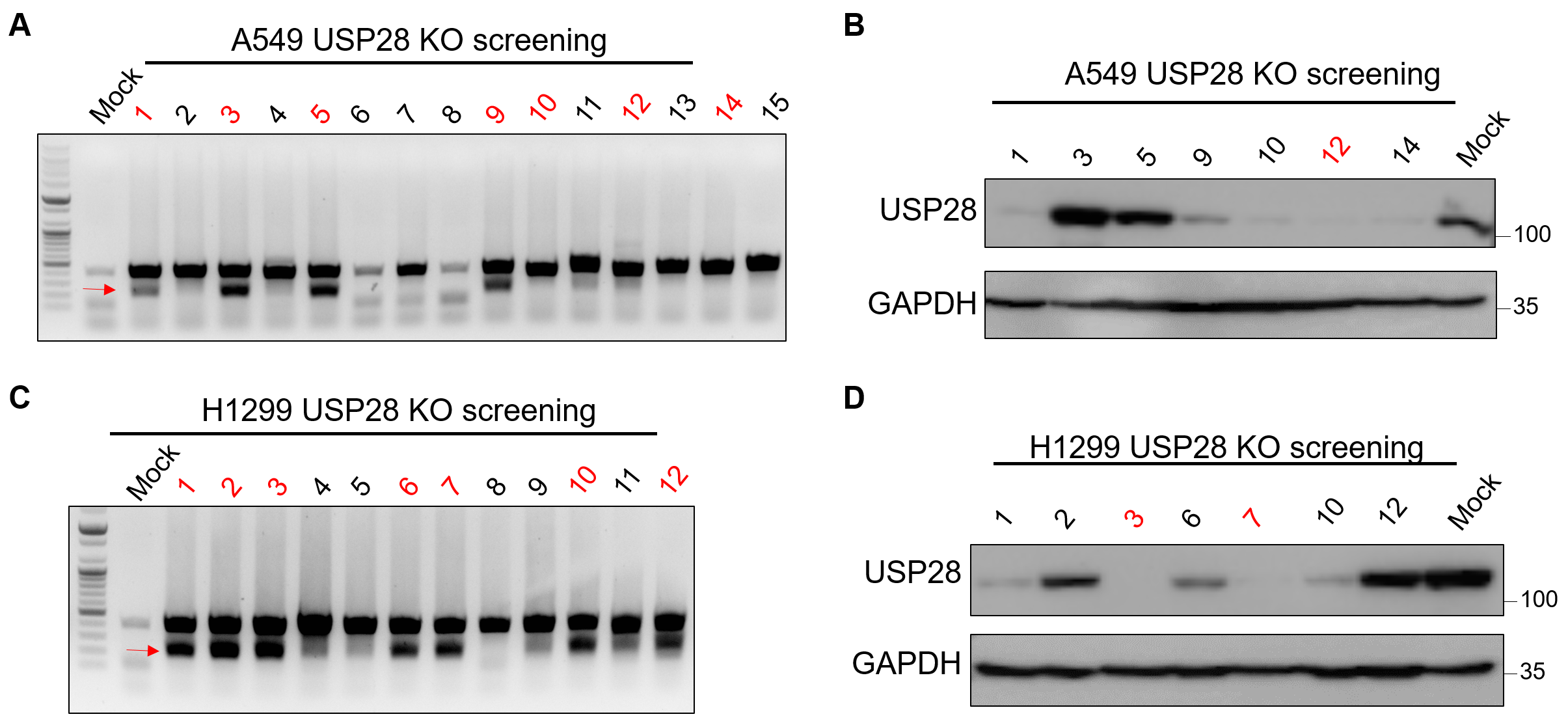


**Supplementary Figure 3.** Screening for USP28 knockout clones in A549 and H1299 cells. The A549 and H1299 cells were transfected with Cas9 and sgRNAs targeting USP28 to generate USP28-KO cell line. **(A)** The cells were seeded in 96 well plates and grown to form colony. The USP28 knockout clone in A549 cell line was screened by T7E1 assay. The T7E1 positive clones showing cleavage are represented in red color. The arrow indicates the cleaved bands of the PCR amplicons **(B)** Western blot analysis showing USP28 knockout efficiency in A549 cells. **(C)** The USP28 knockout clone in H1299 cell line was screened by T7E1 assay. The T7E1 positive clones showing cleavage are represented in red color. The arrow indicates the cleaved bands of the PCR amplicons. **(D)** Western blot analysis showing USP28 knockout efficiency in H1299 cells. The T7E1 negative clone was used as a mock control. GAPDH was used as the internal loading control. The T7E1 negative clone was used as a mock control. GAPDH was used as the internal loading control.

**
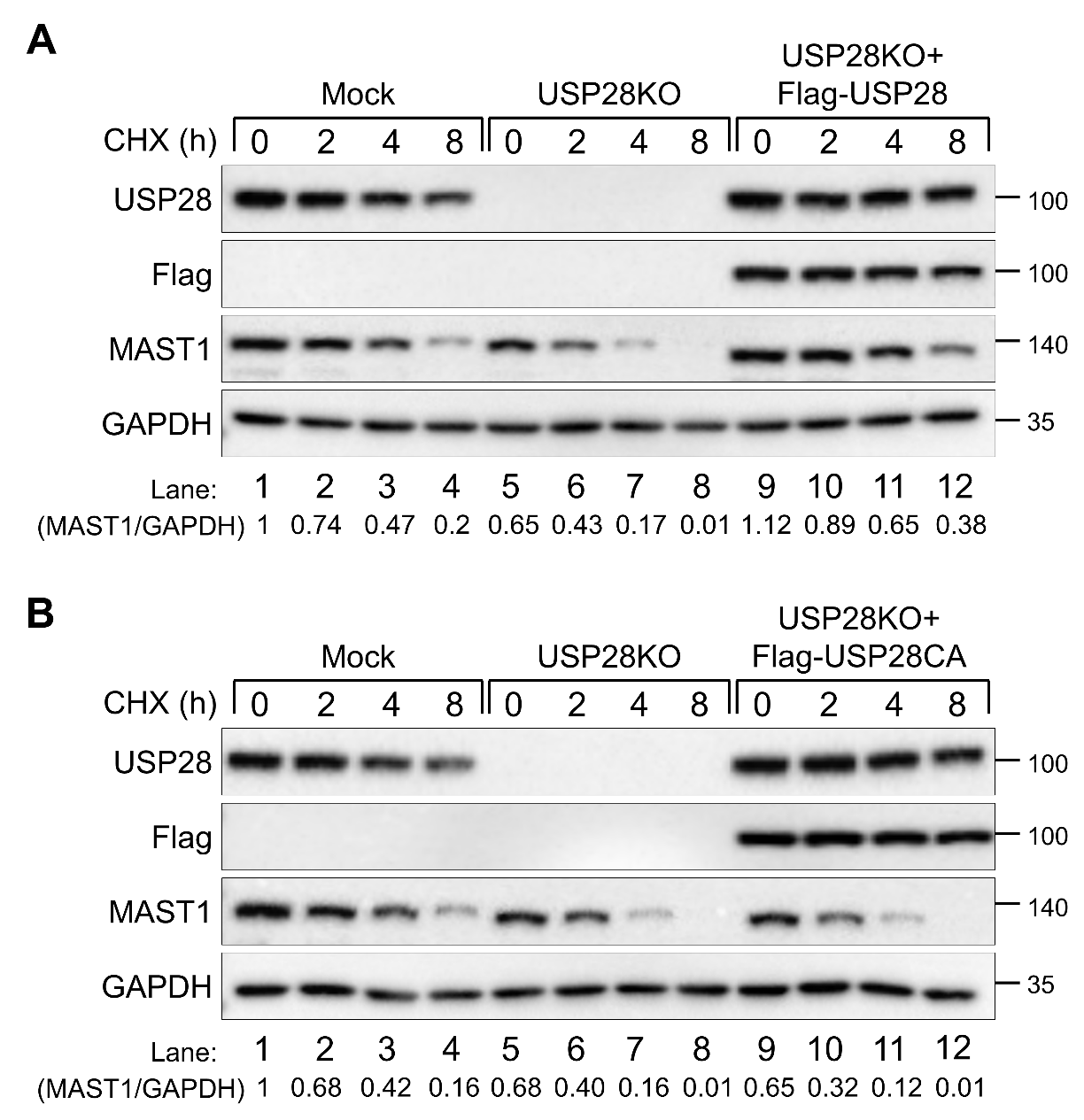
**

**Supplementary Figure 4.** USP28 extends the half-life of MAST1 in H1299 cells. **(A-B)** The effect of USP28-KO on the half-life of MAST1 in H1299 cells. The mock control, USP28-KO and USP28-KO cells reconstituted with (A) Flag-USP28 and (B) Flag-USP28CA were treated with CHX (150 μg/mL) and harvested at the indicated time interval. The cells were then subjected for western blotting with the indicated antibodies. The protein band intensities were estimated using ImageJ software with reference to the GAPDH control.


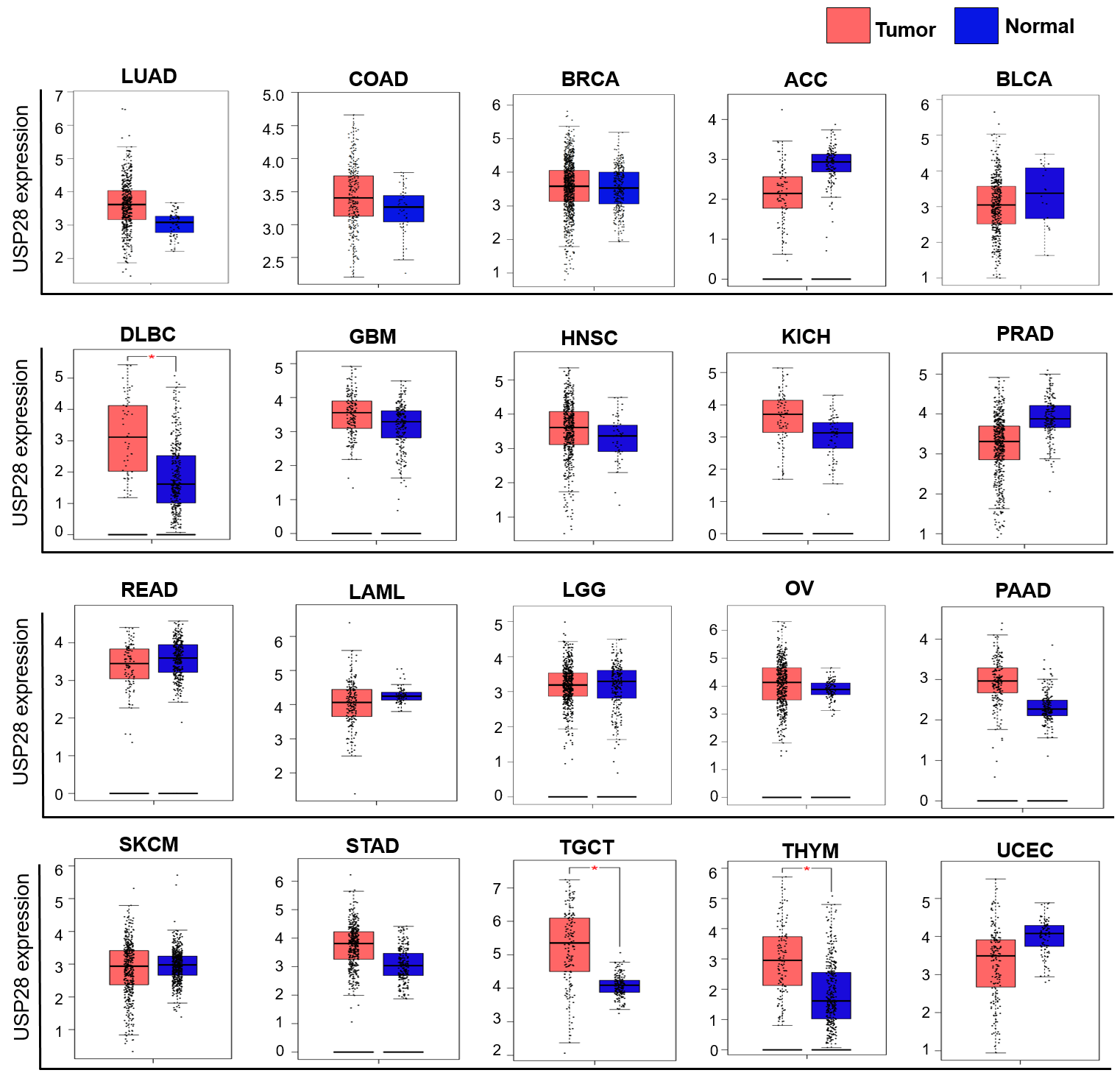


**Supplementary Figure 5.** The mRNA expression profile of USP28 in various cancers and normal tissues. Box plot showing difference between USP28 expression in tumor and normal tissues in different cancer types.


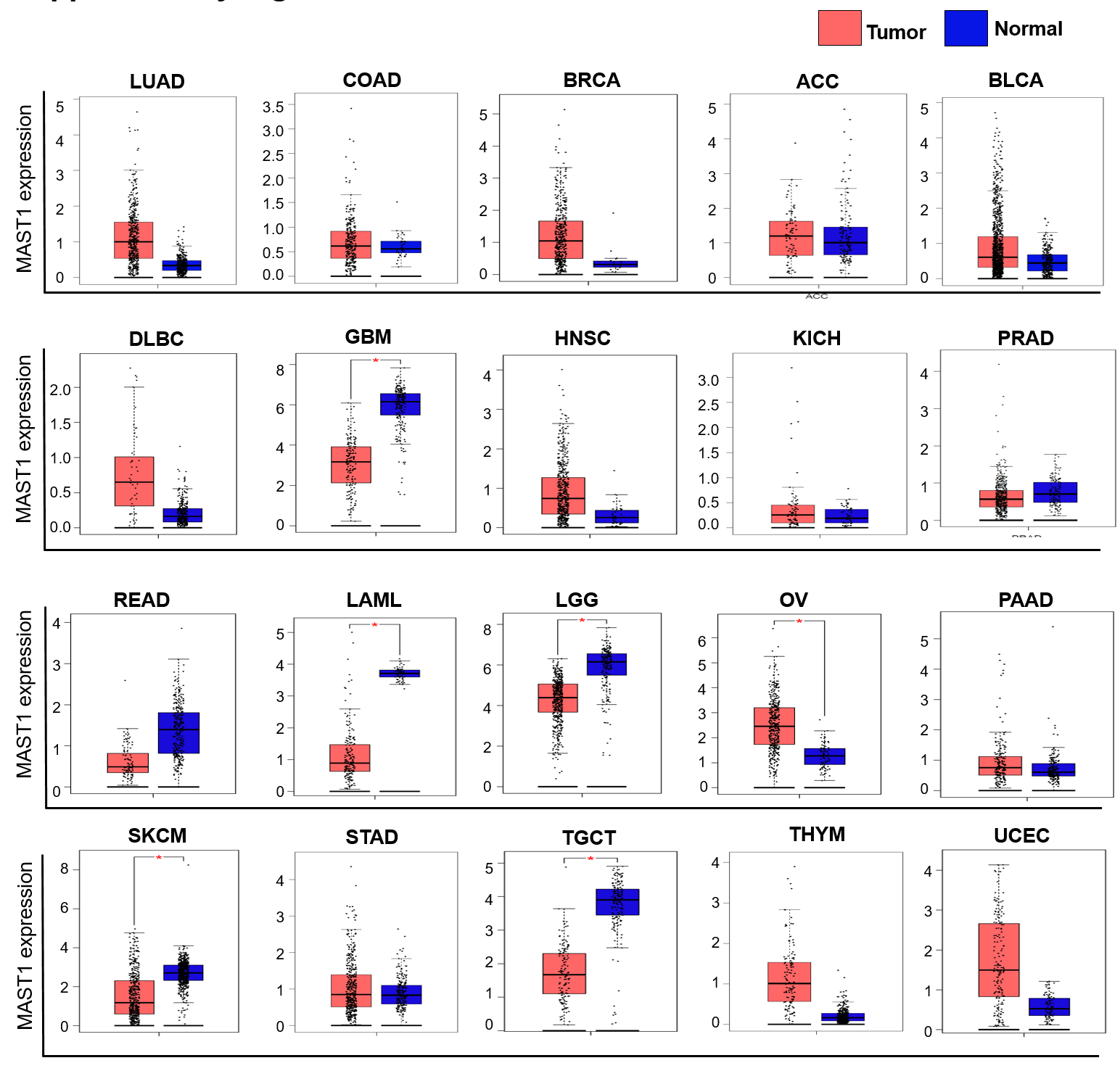


**Supplementary Figure 6.** The mRNA expression profile of MAST1 in various cancers and normal tissues. Box plot showing difference between MAST1 expression in tumor and normal tissues in different cancer types.

**
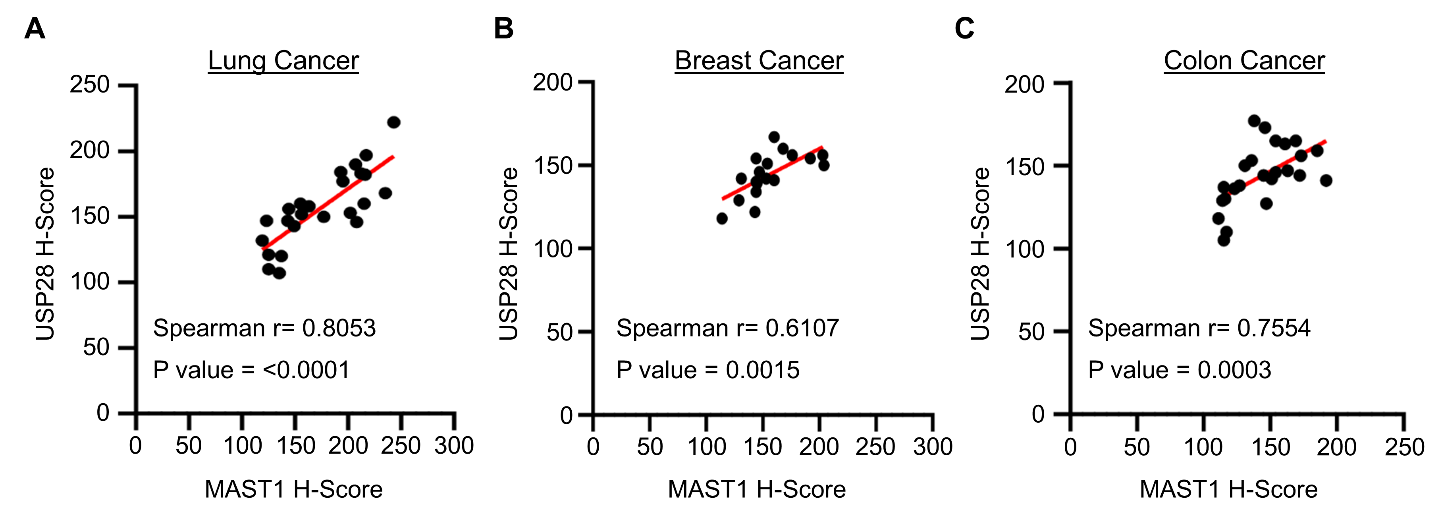
**

**Supplementary Figure 7.** Non-parametric spearmen correlation test for IHC staining of cancer tissues. **(A)** lung cancer (n= 24 ), **(B)** breast cancer (n= 18) and **(C)** colon cancer (n= 24) patients.

**
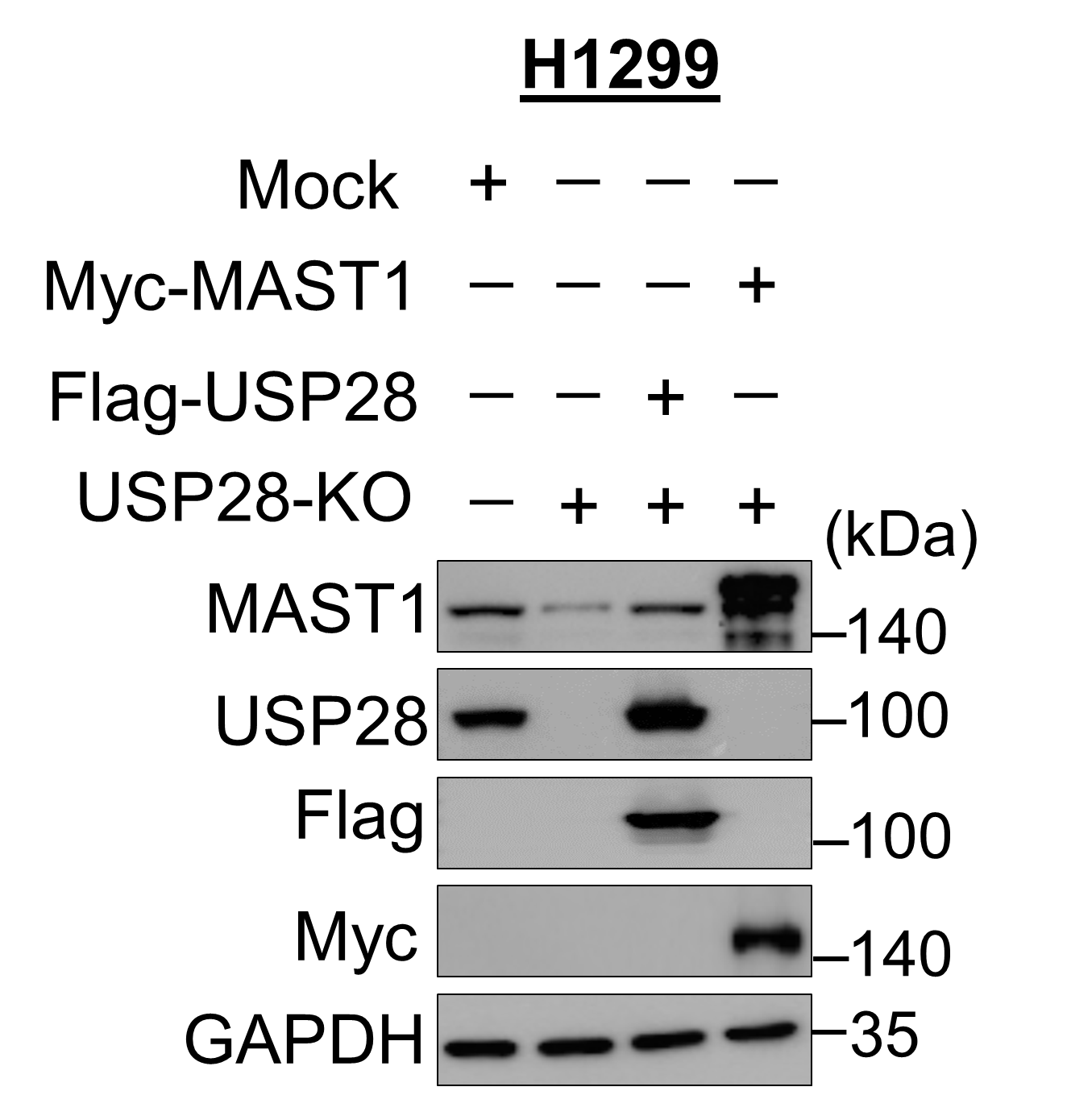
**

**Supplementary Figure S8.** Western blot analysis to validate the expression of endogenous USP28 and MAST1 in USP28-KO H1299 cells. USP28-KO H1299 cells reconstituted with either Flag-USP28 or Myc-MAST1 were subjected to immunoblotting with the indicated antibodies. GAPDH was used as internal loading control.

**
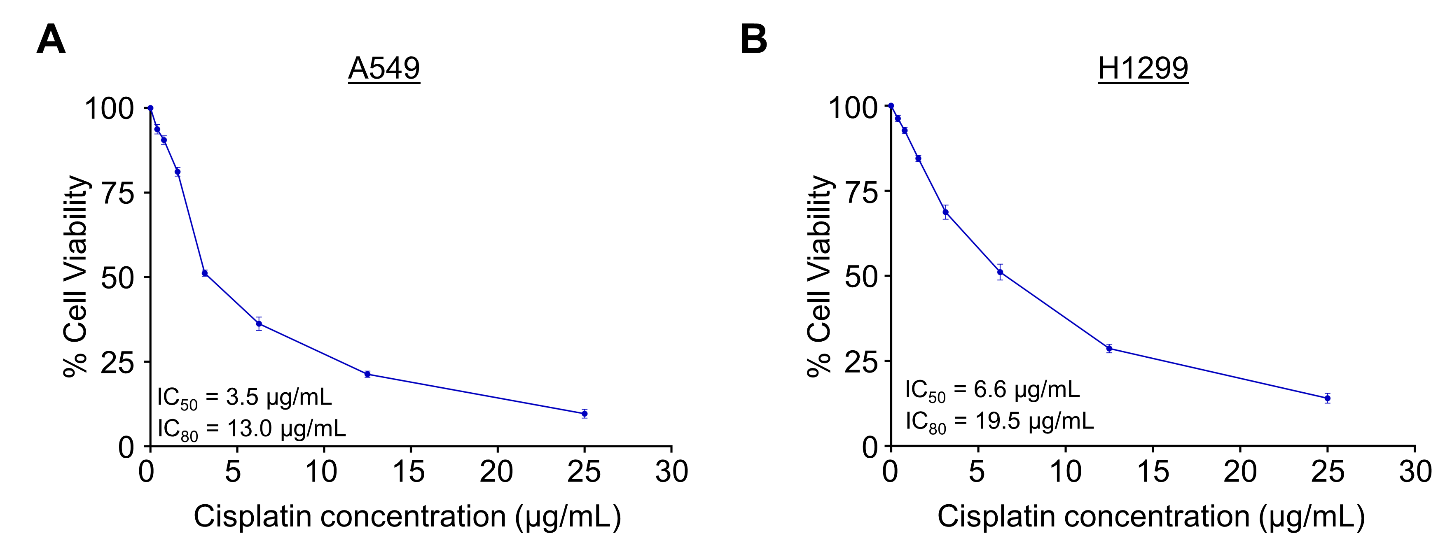
**

**Supplementary Figure 9.** In vitro dose response curve of cisplatin in A549 and H1299 cell lines. **(A)** A549 **(B)** H1299 cells were treated with increasing concentration of cisplatin for 48h and cell viability was assessed by CCK-8 kit. Data are presented as the means and standard deviations of 3 independent experiments.

**
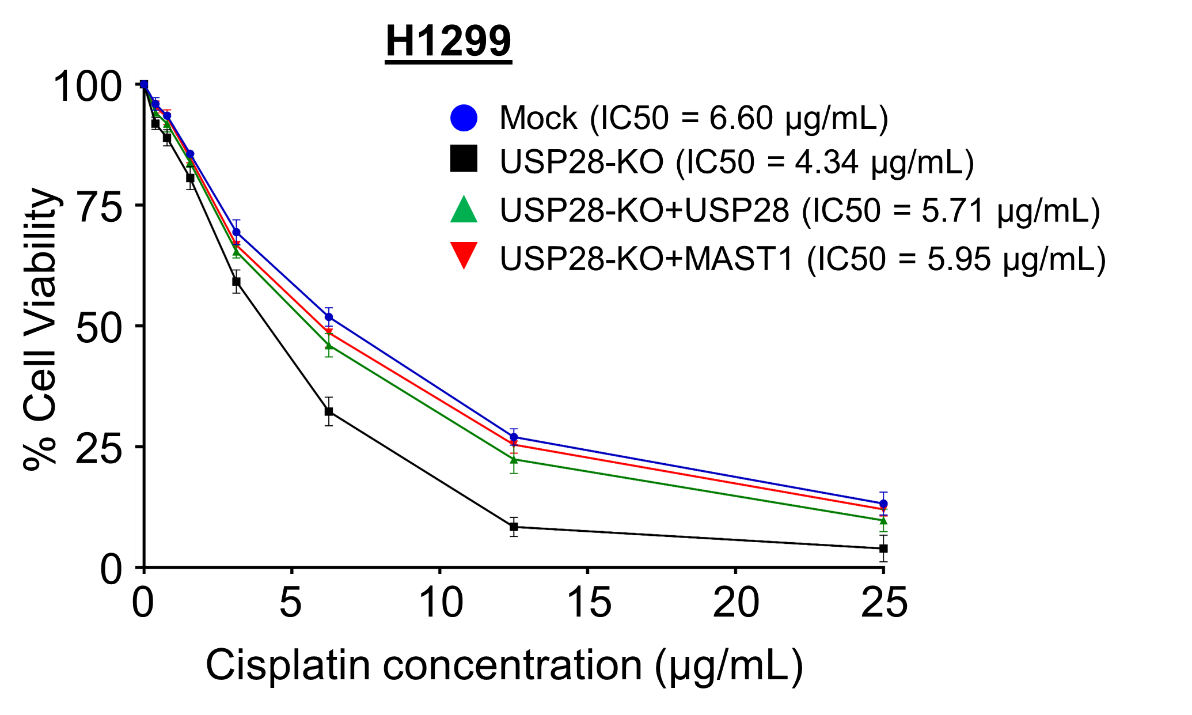
**

**Supplementary Figure 10.** Cell viability assay in H1299 cells treated with increasing concentration of cisplatin (5 µg/mL, 10 µg/mL, 15 µg/mL, 20 µg/mL and 25 µg/mL) by using CCK-8 kit. Data are presented as the mean and standard deviation of three independent experiments (n = 3). The IC_50_ values of cisplatin in H1299 mock, USP28-KO and USP28-KO reconstituted with USP28 and USP28-KO reconstituted with MAST1 were 6.60 µg/mL, 4.34 µg/mL, 5.71 µg/mL, and 5.95 µg/mL, respectively.

**
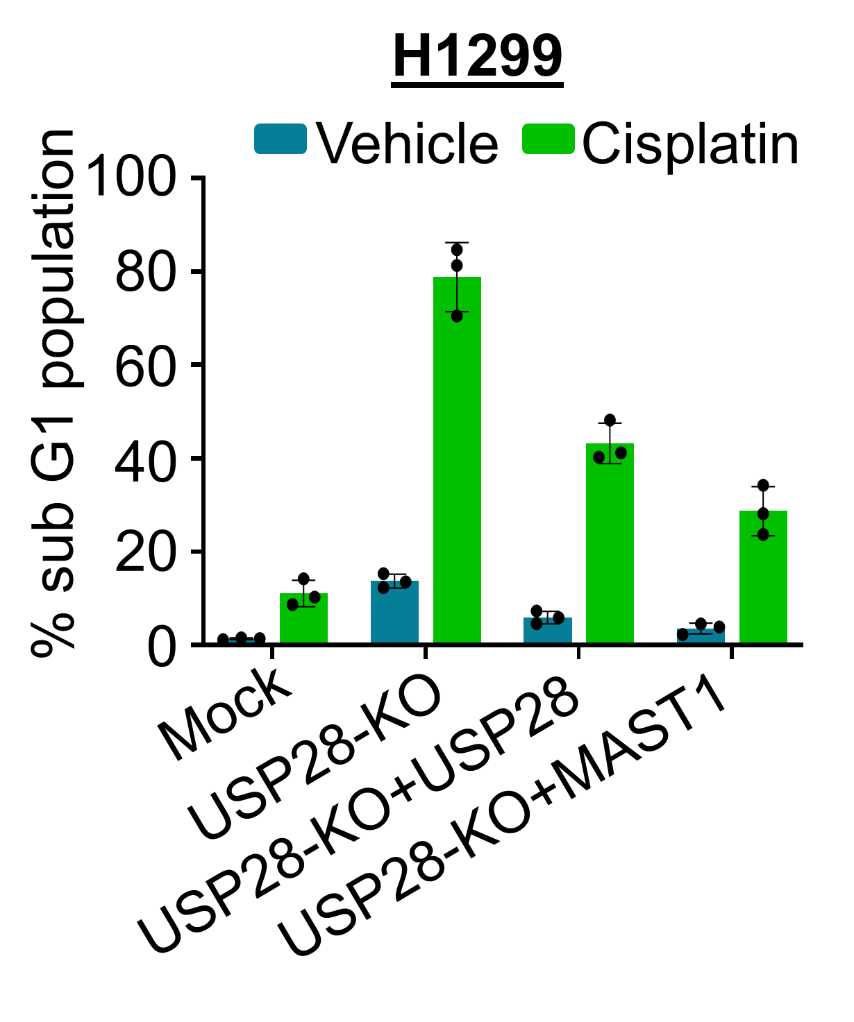
**

**Supplementary Figure 11.** Estimating sub-G1 population in H1299 cells by flow cytometry**.** H1299 cells were treated with cisplatin (5 µg/mL) for 48 h and subjected to flow cytometry to measure the DNA content using PI staining and Data are presented as the mean and standard deviation of three independent experiments (n = 3).

**
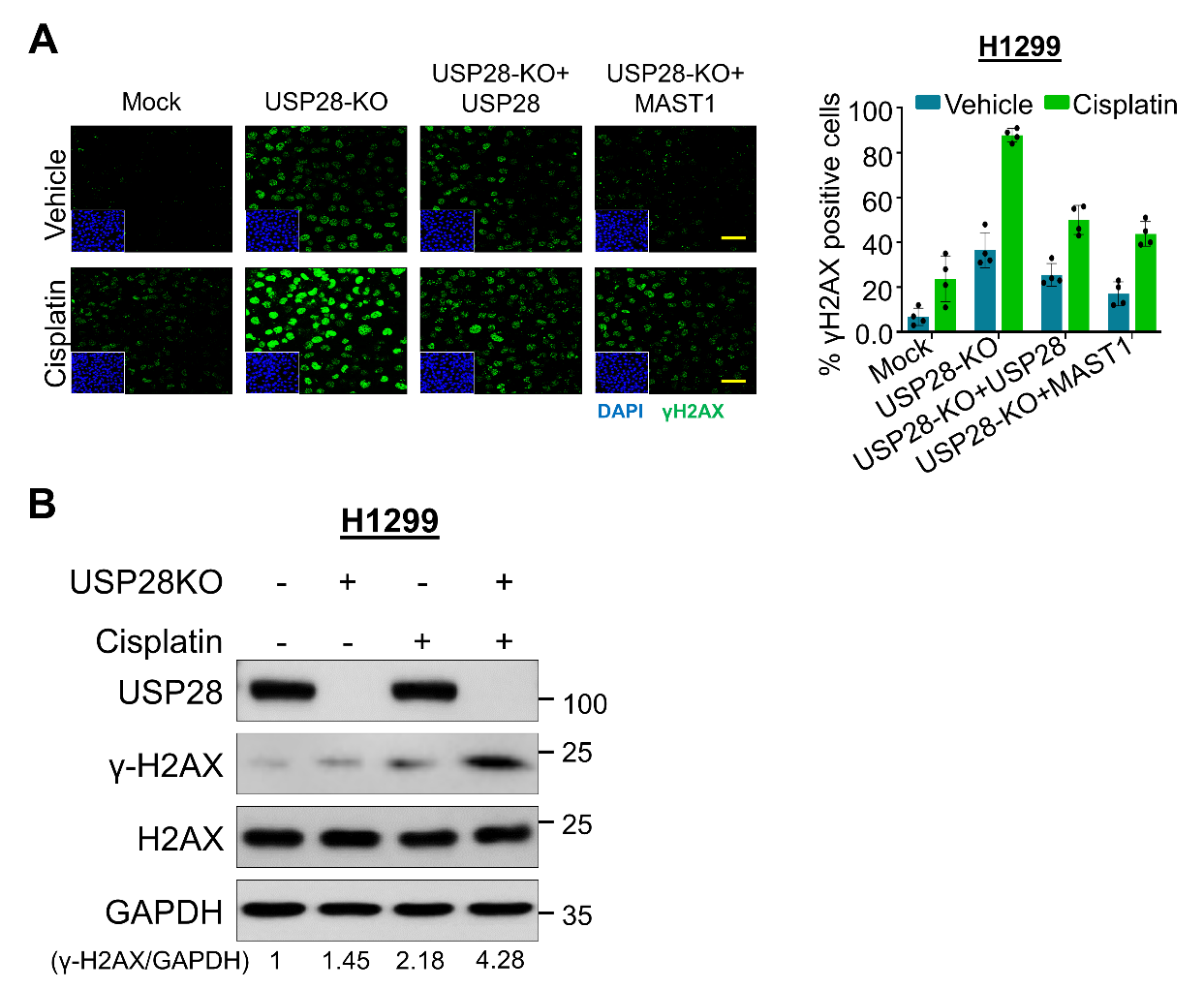
**

**Supplementary Figure 12.** Loss of USP28 promotes DNA damage in H1299 cells. **(A)** Mock control, USP28-KO, and USP28-KO H1299 cells reconstituted with either USP28 or MAST1 were treated with either vehicle or cisplatin (5 µg/mL) for 48 h and subjected to immunofluorescence analysis to estimate γH2AX foci formation. Green, γH2AX; blue, nucleus stained by DAPI. Scale bar = 100 µm. The right panel depicts the percentage of γH2AX-positive cells. Data are presented as the mean and standard deviation of three independent experiments (n = 3). **(B)** H1299 cells treated with cisplatin (5 µg/mL) for 48 h were subjected to immunoblotting analysis with the indicated antibodies. The protein band intensities were estimated using ImageJ software with reference to the GAPDH control (γ-H2AX/GAPDH) and presented below the blot.

**
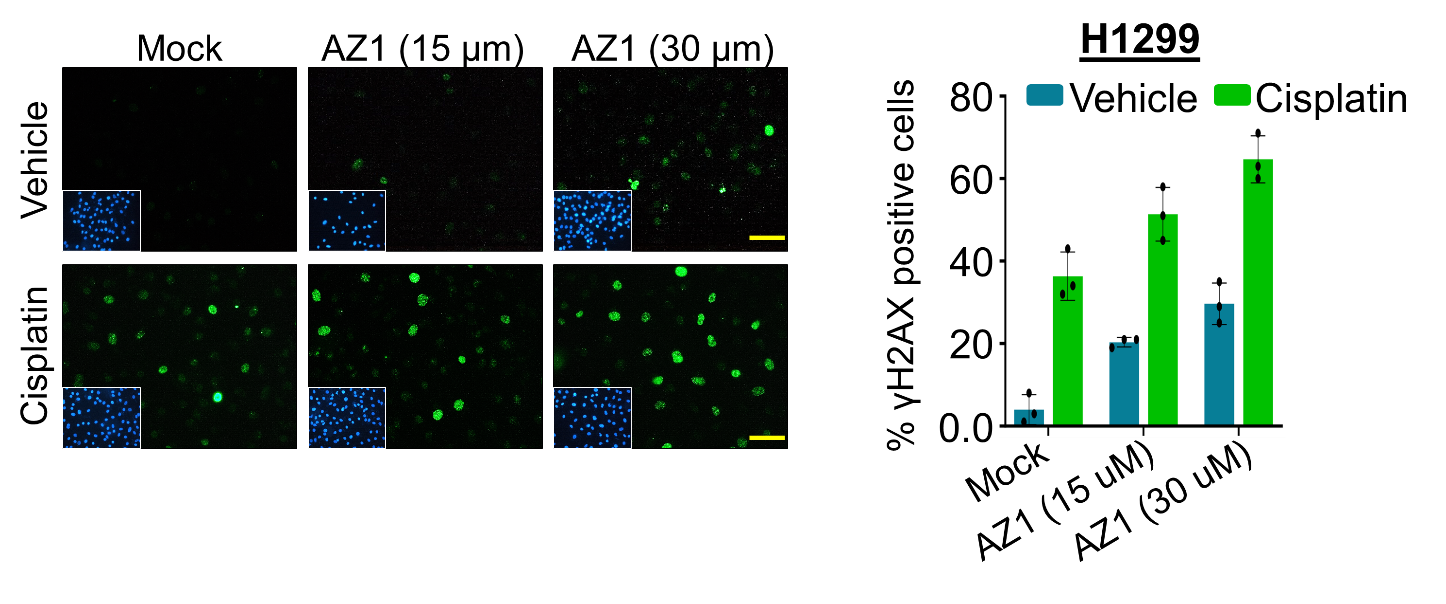
**

**Supplementary Figure 13.** γH2AX foci formation in H1299 cells treated with USP28 inhibitor (AZ1) by immunofluorescence analysis. H1299 cells were treated with the indicated concentrations of USP28 inhibitor (AZ1) with either vehicle or cisplatin (5 µg/mL) for 48 hours and subjected to immunofluorescence analysis to estimate γH2AX foci formation. Green, γH2AX; blue, nucleus stained by DAPI. Scale bar = 100 µm. The right panel depicts the percentage of γH2AX-positive cells. Data are presented as the mean and standard deviation of three independent experiments (n = 3).

**
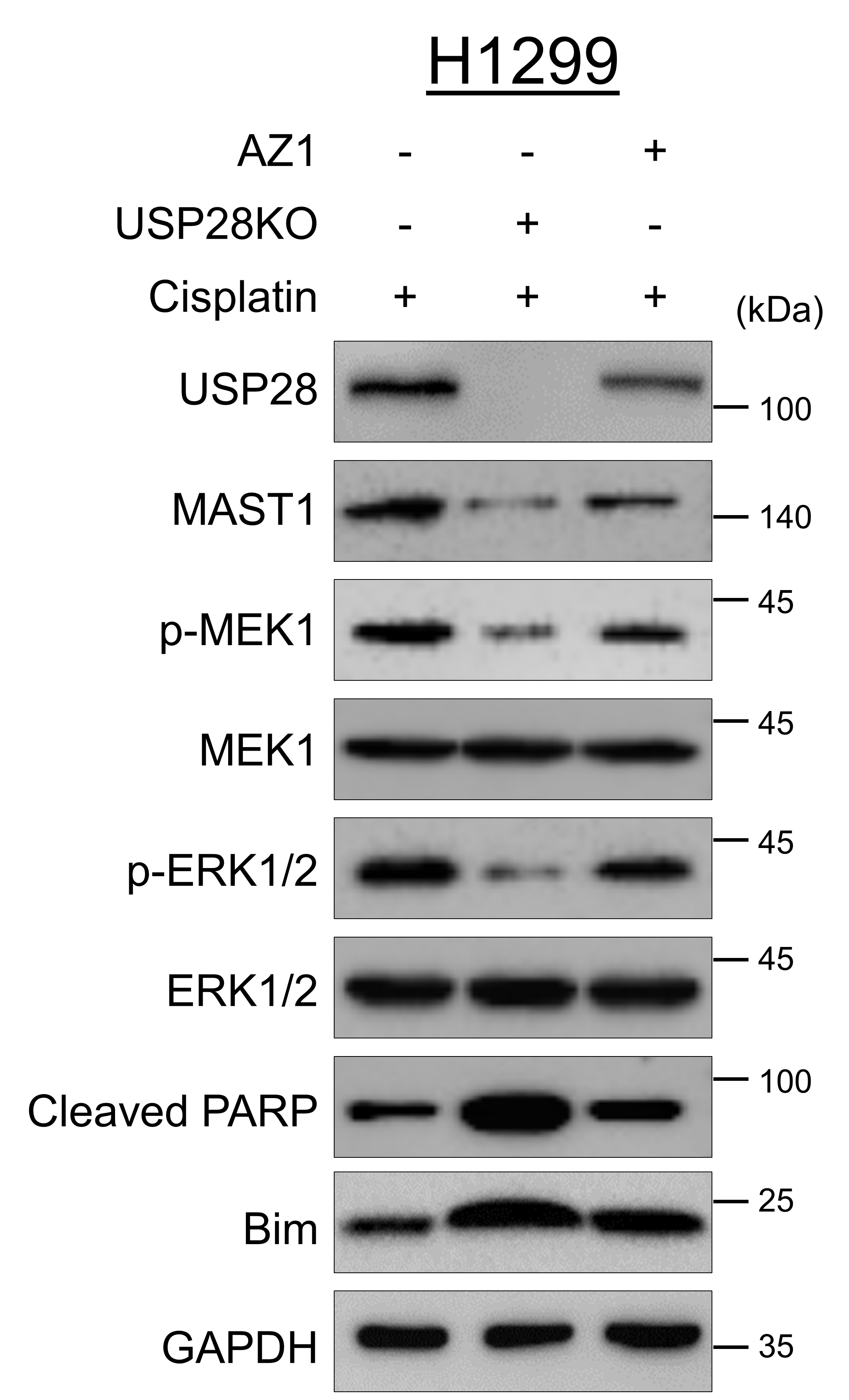
**

**Supplementary Figure 14.** The regulation of USP28 on MAST1-mediated MEK1 activation in H1299 cells. H1299 cells were treated with cisplatin (5 µg/mL) in the presence or absence of USP28 inhibitor (AZ1) for 48 h, and MEK1 activation and apoptosis-related factors were determined using western blotting. GAPDH was used as the internal loading control.


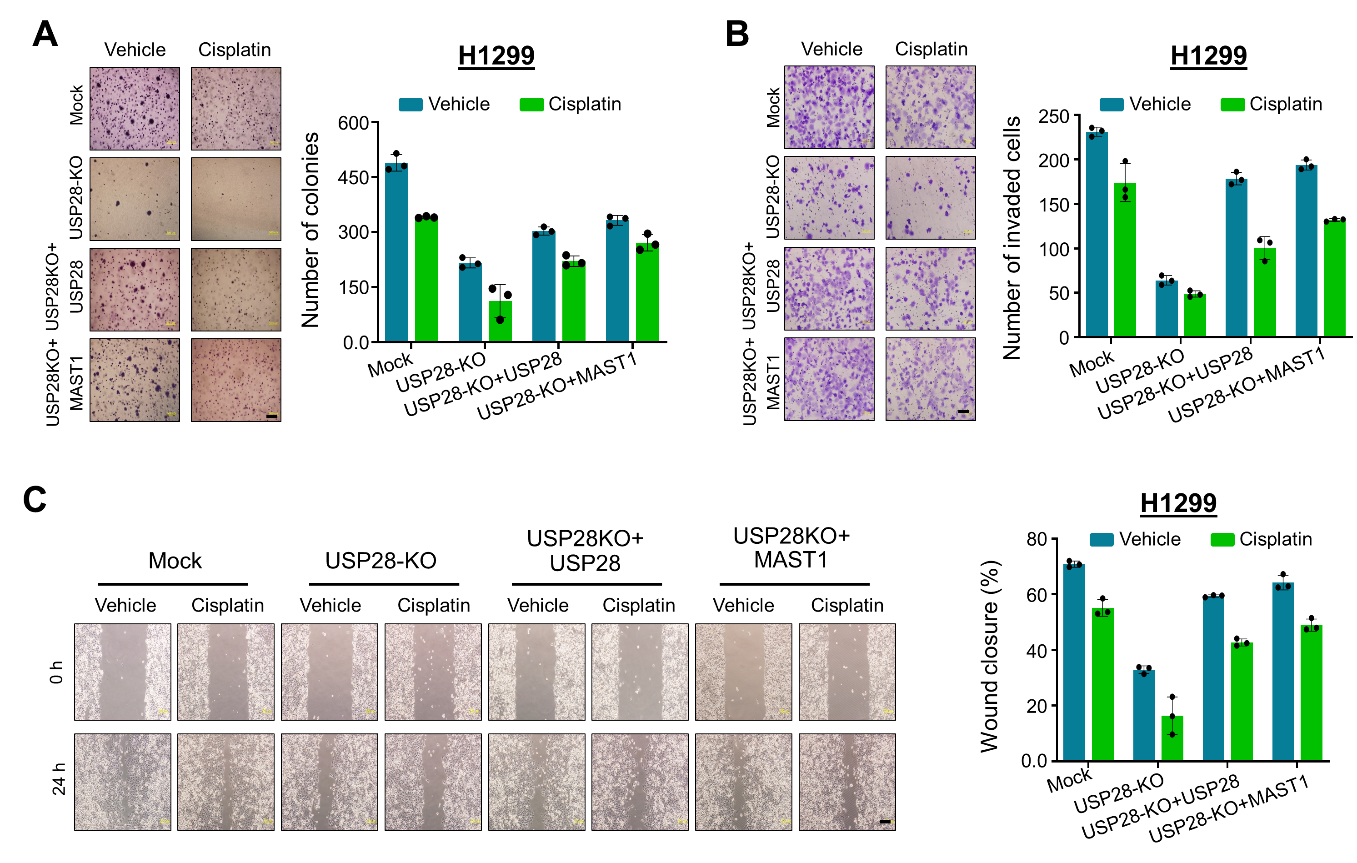


**Supplementary Figure 15.** Loss of USP28 inhibits tumorigenesis in H1299 cells. The H1299 Mock control, USP28-KO, and USP28-KO cells reconstituted with either USP28 or MAST1 were used to perform the following experiments. The cells were subjected to **(A)** Colony formation was measured after 14 days. The colony numbers were quantified and are presented graphically. Scale bar, 500 µm **(B)** The transwell cell invasion assay was performed with the groups mentioned. Scale bar, 100 µm. The number of invaded cells were quantified using ImageJ software and represented graphically. Data are presented as the means and standard deviations of 3 independent experiments. **(C)** The migration potential of above mentioned groups was assessed by an *in vitro* scratch assay. The migration potential was quantified by ImageJ software and is presented graphically. Scale bar, 200 µm. Data are presented as the means and standard deviations of 3 independent experiments. One-way ANOVA followed by Tukey’s post hoc test was used with the indicated *P* values.

**Supplementary Table S1.** Target sequences used for sgRNA plasmid construction.

| **Gene** | **sgRNA** | **Direction** | **Sequence (5’ to 3’)** | **Orientation** |
| --- | --- | --- | --- | --- |
| ***USP28*** | sgRNA1 | FP | GAGAGAAATCACAGGCATTC | Sense |
|  |  | RP | GAATGCCTGTGATTTCTCTC |  |
|  | sgRNA2 | FP | CTTTCTCCATGAAGCTCTGA | Sense |
|  |  | RP | TCAGAGCTTCATGGAGAAAG |  |

**Supplementary Table S2.** Oligonucleotide sequences used to get PCR amplicon for T7E1 assay.

| **Gene** | **sgRNA** |  | **Direction** | **Sequence (5’ to 3’)** |
| --- | --- | --- | --- | --- |
| ***USP28*** | sgRNA1 | I PCR | FP | TCTTGGAGCTTGTTGGTTCTT |
|  |  |  | RP | CAGGGTTACCCGAACCACAA |
|  |  | II PCR | FP | GCCTCAGGCCTGTTTATCCAAG |
|  |  |  | RP | CAGGGTTACCCGAACCACAA |

**Supplementary Table S3.** PCR amplicon and cleavage sizes after T7E1 assay.

| **Gene** | **sgRNA** | **PCR size** | **Cleavage size** |
| --- | --- | --- | --- |
| ***USP28*** | sgRNA1 | 378 | 146+232 |
|  | sgRNA2 | 378 | 176+202 |

**Supplementary Table S4. Oligonucleotides sequence used for qRT-PCR.**

| **Gene** | **Direction** | **Sequence (5’ to 3’)** |
| --- | --- | --- |
| *USP28* | FP | TTAGACGAGTGTTTGGAAGGG |
|  | RP | ACGCTCTTGTCCATACTTCAC |
| *MAST1* | FP | TCTCTGGACCGCGCTTTCTA |
|  | RP | TGAGGCTTTTCCGATTACTGGT |
| *GAPDH* | FP | CATGTTCGTCATGGGTGTGAACCA |
|  | RP | AGTGATGGCATGGACTGTGGTCAT |

**Supplementary Table S5. The mRNA scores for *USP28* and *MAST1* expression derived from the Cancer Cell Line Encyclopedia database in various cancer cell lines.**

| **MAST1 expression log2(TPM+1)** | **USP28 Expression log2(TPM+1)** | **Cell Line Name** |
| --- | --- | --- |
| 4.414812061 | 7.661635602 | CORL311 |
| 2.454175893 | 7.329392731 | NCIH520 |
| 3.878725341 | 7.265005493 | NCIH526 |
| 1.974529312 | 6.274261661 | HSQ89 |
| 1.815575429 | 6.18030702 | SNU738 |
| 1.150559677 | 6.038918989 | HCC15 |
| 3.965322548 | 6.033643244 | JEKO1 |
| 1.292781749 | 5.928844037 | UMUC4 |
| 3.22342255 | 5.928844037 | HT |
| 2.95419631 | 5.906890596 | LK2 |
| 3.932628157 | 5.794935663 | NCIH660 |
| 1.66448284 | 5.758089934 | NCIH1568 |
| 3.816599707 | 5.675815931 | CORL88 |
| 6.338246117 | 5.674121633 | DMS79 |
| 3.102658131 | 5.628481994 | INA6 |
| 5.884109451 | 5.604664415 | NCIH1105 |
| 2.275007047 | 5.595742339 | VCAP |
| 1.516015147 | 5.588864518 | SW837 |
| 1.344828497 | 5.567119488 | EFO21 |
| 4.109360559 | 5.540089282 | MHHES1 |
| 2.23572706 | 5.522306893 | PA1 |
| 2.503348735 | 5.506208389 | HCC366 |
| 4.132576843 | 5.501120634 | LOUCY |
| 2.339137385 | 5.486070744 | LN215 |
| 4.012568674 | 5.475409193 | NCIH1048 |
| 3.512226887 | 5.462052319 | SLR20 |
| 3.367371066 | 5.454175893 | TGBC11TKB |
| 1.014355293 | 5.439955517 | LPS141 |
| 1.220329955 | 5.438625538 | NCCIT |
| 2.232660757 | 5.392660881 | FUOV1 |
| 2.695993813 | 5.390942773 | NCIH1819 |
| 3.517275693 | 5.375039431 | HAP1 |
| 2.324810603 | 5.371558863 | L363 |
| 3.798050515 | 5.35473424 | GSS |
| 2.272023189 | 5.353676158 | FLO1 |
| 1.922197848 | 5.351910961 | JR |
| 2.545968369 | 5.348374075 | CAL51 |
| 2.372952098 | 5.346956889 | OUMS23 |
| 1.604071324 | 5.345183447 | MKN74 |
| 1.321928095 | 5.338067798 | SW1573 |
| 1.799087306 | 5.332707934 | KYSE150 |
| 3.334854269 | 5.321928095 | DL40 |
| 1.996388746 | 5.320845668 | NCIH1355 |
| 1.280956314 | 5.313971371 | TOLEDO |
| 1.839959587 | 5.28503225 | TTC442 |
| 2.956056652 | 5.274634403 | SW13 |
| 2.280956314 | 5.274261661 | HS746T |
| 1.929790998 | 5.272396509 | SUIT2 |
| 4.91981677 | 5.270155139 | HCC33 |
| 2.372952098 | 5.266786541 | HCC95 |
| 1.35614381 | 5.266036894 | RHJT |
| 2.769771739 | 5.26190686 | KNS42 |
| 0.613531653 | 5.257010618 | KE39 |
| 2.211012193 | 5.256633295 | MOLP2 |
| 2.280956314 | 5.251340383 | SW1353 |
| 4.280956314 | 5.23878686 | DMS153 |
| 3.790772038 | 5.236492618 | NCIH841 |
| 1.257010618 | 5.229587923 | OC316 |
| 1.10433666 | 5.22342255 | CAL33 |
| 1.66448284 | 5.217230716 | NCIH2228 |
| 4.943921327 | 5.212958363 | NCIH1184 |
| 1.3950628 | 5.211012193 | PATU8988T |
| 1.485426827 | 5.203201156 | NCIH929 |
| 2.160274831 | 5.202809492 | SUM1315MO2 |
| 2.220329955 | 5.196134881 | COLO680N |
| 4.687060688 | 5.191009849 | NCIH2196 |
| 1.469885976 | 5.189824559 | DANG |
| 4.840966704 | 5.17951105 | L540 |
| 1.13093087 | 5.165510018 | JVE253 |
| 5.110613806 | 5.165510018 | CHLA10 |
| 1.250961574 | 5.160274831 | LN340 |
| 1.35614381 | 5.155830172 | KP2 |
| 3.12763328 | 5.154615611 | UMUC3 |
| 1.910732662 | 5.137503524 | HEC59 |
| 4.584962501 | 5.130107179 | NCIH510 |
| 5.157852169 | 5.122258568 | SKNFI |
| 5.421896512 | 5.119356177 | NCIH69 |
| 2.35614381 | 5.11727946 | JJN3 |
| 1.021479727 | 5.114367025 | HCC2814 |
| 1.835924074 | 5.111448698 | TM31 |
| 2.555816155 | 5.105175192 | RD |
| 2.748461233 | 5.098874287 | DMS454 |
| 4.267535798 | 5.09085343 | AN3CA |
| 0.704871964 | 5.088311236 | PATU8902 |
| 5.111448698 | 5.078524445 | Y79 |
| 2.307428525 | 5.075532631 | S462 |
| 2.615887074 | 5.069530325 | EJM |
| 1.049630768 | 5.068670811 | NCIH1651 |
| 2.472487771 | 5.057016965 | HCC1438 |
| 3.089159132 | 5.044394119 | DMS53 |
| 3.572889668 | 5.043519494 | NTERA2CLD1 |
| 2.916476644 | 5.033423002 | TE6 |
| 0.321928095 | 5.027684877 | SISO |
| 0.871843649 | 5.024585638 | UMUC7 |
| 2.687060688 | 5.024142346 | NCIH2126 |
| 2.831877241 | 5.015693807 | REH |
| 3.46727948 | 5.01346226 | TF1 |
| 5.776103988 | 5.008988783 | NH6 |
| 2.976363636 | 5.00270252 | RERFLCAI |
| 2.572889668 | 5.002252452 | MDAMB468 |
| 2.160274831 | 5.001351893 | GP2D |
| 3.221877081 | 5.000901403 | HCC1195 |
| 1.996388746 | 4.995032192 | JHOS4 |
| 2.942983598 | 4.994127114 | U2OS |
| 3.578938713 | 4.993674362 | SKUT1 |
| 3.977279923 | 4.987320866 | CHLA32 |
| 2.553360503 | 4.982765463 | OCIMY7 |
| 1.070389328 | 4.974070367 | OCIM1 |
| 4.593353771 | 4.972233124 | NCIH1341 |
| 1.700439718 | 4.971313624 | JHH6 |
| 2.849999259 | 4.969933275 | LXF289 |
| 1.056583528 | 4.965784285 | SNU899 |
| 1.448900951 | 4.964860664 | NCIH1755 |
| 4.096767855 | 4.963474124 | TE441T |
| 1.15704371 | 4.96208625 | CW9019 |
| 0.992768431 | 4.961160258 | KARPAS620 |
| 2.414135533 | 4.961160258 | ONDA8 |
| 3.032100843 | 4.958378712 | NCIH661 |
| 1.070389328 | 4.945794957 | MDAPCA2B |
| 1.411426246 | 4.943921327 | RERFGC1B |
| 5.507160349 | 4.939226578 | NCIH82 |
| 1.531069493 | 4.936873462 | TT2609C02 |
| 3.037382222 | 4.933100475 | SNU503 |
| 2.881664619 | 4.932155684 | HCC2108 |
| 3.675815931 | 4.9202933 | PFEIFFER |
| 1.516015147 | 4.917909074 | TEN |
| 2.280956314 | 4.916954279 | MHHCALL2 |
| 1.580145484 | 4.908812908 | HCC1954 |
| 3.928844037 | 4.907852072 | CBAGPN |
| 2.992768431 | 4.904484098 | DB |
| 3.475084883 | 4.899659026 | OCILY19 |
| 3.849999259 | 4.896271849 | P3HR1 |
| 3.584962501 | 4.895787316 | G401 |
| 3.017921908 | 4.886062338 | SYO1 |
| 1.23878686 | 4.885086225 | HA1E |
| 2.634593268 | 4.885086225 | HDLM2 |
| 2.744161096 | 4.882153917 | C33A |
| 0.659924558 | 4.879705766 | LS123 |
| 0.855989697 | 4.875288598 | HEC1A |
| 5.388878339 | 4.875288598 | NCIH446 |
| 2.847996907 | 4.869377924 | LC1F |
| 1.195347598 | 4.864433796 | SNU1076 |
| 1.23878686 | 4.863442934 | DERL2 |
| 1.875780063 | 4.862451391 | HCC1937 |
| 2.950468414 | 4.861955364 | NCIH2052 |
| 1.077242999 | 4.860962798 | NCIH1623 |
| 1.3950628 | 4.859472667 | PEA1 |
| 3.890446693 | 4.853995647 | MFE296 |
| 3.554588852 | 4.851499202 | OAW28 |
| 0.925999419 | 4.850999395 | RERFLCAD1 |
| 1.97819563 | 4.843481425 | C8166 |
| 1.790772038 | 4.840463234 | LN18 |
| 1.914564523 | 4.837943242 | RPMI8402 |
| 4.90014226 | 4.836934011 | NCIH1930 |
| 2.422233001 | 4.83541884 | NCIH441 |
| 2.813524689 | 4.831877241 | SW48 |
| 2.513490746 | 4.830356747 | CALU1 |
| 2.957914599 | 4.826802684 | NCIH727 |
| 1.339137385 | 4.82527683 | MB1 |
| 1.632268215 | 4.824258697 | ME1 |
| 2.163498732 | 4.820178962 | GRANTA519 |
| 1.650764559 | 4.812498225 | KURAMOCHI |
| 5.106013238 | 4.806839582 | NCIH1436 |
| 1.207892852 | 4.805808349 | ST486 |
| 1.914564523 | 4.804776378 | PLCPRF5 |
| 3.163498732 | 4.803743669 | DND41 |
| 1.695993813 | 4.801158656 | MKN7 |
| 2.111031312 | 4.798569004 | CAL120 |
| 1.280956314 | 4.79753184 | LN428 |
| 1.82374936 | 4.793375711 | HCC827GR5 |
| 2.381283373 | 4.792334806 | SNU46 |
| 2.266036894 | 4.789207575 | IALM |
| 1.14404637 | 4.77925972 | NP5 |
| 1.782408565 | 4.778734244 | SNU1040 |
| 1.117695043 | 4.777682717 | SW620 |
| 3.209453366 | 4.776103988 | MOLM16 |
| 4.807354922 | 4.771357409 | SUPM2 |
| 4.371558863 | 4.770829046 | CA46 |
| 1.169925001 | 4.767125077 | SNU175 |
| 1.195347598 | 4.766595162 | LUDLU1 |
| 3.176322773 | 4.764473551 | NCIH522 |
| 2.14404637 | 4.762880293 | UW228 |
| 2.298658316 | 4.758622982 | U251MGDM |
| 1.752748591 | 4.754887502 | SAOS2 |
| 3.697106574 | 4.753818443 | TE617T |
| 3.12763328 | 4.746312766 | HCC1187 |
| 2.039138394 | 4.744699314 | HCC2450 |
| 2.111031312 | 4.744699314 | SKOV3 |
| 1.124328135 | 4.743622676 | SCMCRM2 |
| 1.454175893 | 4.743622676 | PANC1 |
| 0.925999419 | 4.739848103 | HUH6 |
| 1.5360529 | 4.739308071 | HT1376 |
| 1.389566812 | 4.734438666 | PACADD119 |
| 1.555816155 | 4.733354341 | HCT15 |
| 4.080657663 | 4.732811872 | NCIH2029 |
| 0.713695815 | 4.731183242 | HCC2429 |
| 0.555816155 | 4.730096466 | NB4 |
| 1.914564523 | 4.730096466 | NOS1 |
| 3.266036894 | 4.730096466 | CME1 |
| 3.631104282 | 4.730096466 | TC138 |
| 1.807354922 | 4.725195817 | T24 |
| 1.565597176 | 4.72410452 | NCIH2882 |
| 2.477677328 | 4.722466024 | CMK |
| 2.121015401 | 4.720825666 | RL |
| 2.344828497 | 4.720825666 | T3M10 |
| 2.752748591 | 4.720825666 | BIN67 |
| 1.310340121 | 4.720278465 | OC314 |
| 1.316145742 | 4.71918344 | OELE |
| 2.845991771 | 4.71918344 | SUDHL10 |
| 2.014355293 | 4.718635616 | JHUEM3 |
| 2.295723025 | 4.716990894 | NCCMPNST2C1 |
| 3.065227623 | 4.716442237 | MJ |
| 2.327687364 | 4.715893371 | SNU119 |
| 2.861955364 | 4.715344295 | P30OHK |
| 4.786073552 | 4.715344295 | NCIH209 |
| 1.678071905 | 4.714795011 | UWB1289 |
| 3.09592442 | 4.714245518 | REC1 |
| 1.448900951 | 4.712045449 | UOK101 |
| 1.070389328 | 4.711494907 | COLO783 |
| 4.03562391 | 4.709842019 | HEC108 |
| 1.195347598 | 4.709290636 | SR786 |
| 3.553360503 | 4.70763522 | TOV21G |
| 3.280956314 | 4.704871964 | G292CLONEA141B1 |
| 2.316145742 | 4.704318678 | OCILY7 |
| 0.622930351 | 4.703765179 | KMS28BM |
| 2.650764559 | 4.703765179 | NCIH2030 |
| 6.321928095 | 4.703765179 | KPNRTBM1 |
| 4.121844298 | 4.703211467 | A2780 |
| 1.948600847 | 4.699329526 | CAL12T |
| 1.327687364 | 4.697106574 | SUSA |
| 1.454175893 | 4.693765712 | 8505C |
| 1.304511042 | 4.689858236 | SF126 |
| 4.473137488 | 4.688180359 | WSUDLCL2 |
| 2.713695815 | 4.687620632 | NCIH157DM |
| 1.169925001 | 4.687060688 | RH28 |
| 2.746312766 | 4.682573297 | LPS853 |
| 1.765534746 | 4.680886921 | H4 |
| 2.214124805 | 4.680886921 | LN382 |
| 2.372952098 | 4.676944359 | COLO824 |
| 3.22342255 | 4.674121633 | OVSAHO |
| 2.087462841 | 4.671293372 | RH4 |
| 1.438292852 | 4.6622055 | NCIH647 |
| 1.650764559 | 4.661635602 | NCIH1693 |
| 1.790772038 | 4.66106548 | JM1 |
| 1.765534746 | 4.660495132 | U118MG |
| 1.752748591 | 4.658211483 | BT20 |
| 1.843983844 | 4.658211483 | LOUNH91 |
| 3.481557281 | 4.658211483 | NALM6 |
| 5.638363506 | 4.654779217 | NCIH1092 |
| 4.701549057 | 4.652486495 | NCIH1876 |
| 3.731183242 | 4.651912745 | EWS502 |
| 2.003602237 | 4.651338766 | KPMRTRY |
| 1.5360529 | 4.648465443 | UMUC11 |
| 1.963474124 | 4.64731451 | SKMM2 |
| 3.080657663 | 4.646738698 | D425 |
| 1.82374936 | 4.643278996 | RL952 |
| 2.153805336 | 4.642701572 | TM87 |
| 2.90303827 | 4.64096791 | AMO1 |
| 2.5360529 | 4.64038956 | BT549 |
| 2.204766751 | 4.639810978 | A1207 |
| 1.575312331 | 4.638073837 | NP8 |
| 2.646162657 | 4.632268215 | PSS008 |
| 2.9800253 | 4.631686366 | KMS11 |
| 2.879705766 | 4.624100895 | SUDHL5 |
| 0.344828497 | 4.623515741 | PATU8988S |
| 2.22342255 | 4.62058641 | KYSE520 |
| 3.114367025 | 4.618238656 | KMS12BM |
| 0.963474124 | 4.617651119 | MCAS |
| 2.324810603 | 4.616475329 | NCIH2085 |
| 2.910732662 | 4.616475329 | NCIH1581 |
| 3.386810946 | 4.616475329 | SKNSH |
| 3.64385619 | 4.616475329 | OVCAR4 |
| 2.689299161 | 4.614120869 | MCC13 |
| 1.042644337 | 4.608217853 | UPCISCC152 |
| 3.918386234 | 4.608217853 | COGAR359 |
| 4.749534268 | 4.607626221 | HEC6 |
| 4.202417722 | 4.606442228 | EN |
| 2.794935663 | 4.602290585 | RH30 |
| 1.14404637 | 4.599912842 | SF268 |
| 2.266036894 | 4.5987225 | WSUNHL |
| 3.340562269 | 4.596338864 | MDAMB435S |
| 3.217230716 | 4.595742339 | BCPAP |
| 1.709290636 | 4.59454855 | NCIH2342 |
| 2.166715445 | 4.59275601 | T47D |
| 2.528571319 | 4.588564737 | KP4 |
| 3.86393845 | 4.586164246 | TASK1 |
| 3.19061486 | 4.584361253 | NUDHL1 |
| 3.63691458 | 4.581953751 | SKNEP1 |
| 3.426264755 | 4.580145484 | HDMYZ |
| 1.411426246 | 4.578938713 | UACC257 |
| 3.545968369 | 4.578938713 | TTC549 |
| 1.214124805 | 4.574707046 | CCC5 |
| 3.267535798 | 4.568640195 | COLO741 |
| 3.286881148 | 4.567423758 | RDES |
| 1.963474124 | 4.56193706 | MESOV |
| 1.941106311 | 4.561326136 | NCIH1648 |
| 1.761285273 | 4.560714954 | KU812 |
| 5.426600226 | 4.560103513 | CORL24 |
| 3.451540833 | 4.557655155 | SNUC2A |
| 3.111031312 | 4.555816155 | IHH4 |
| 1.89917563 | 4.554588852 | SKLMS1 |
| 1.384049807 | 4.548436625 | TGBC1TKB |
| 1.40599236 | 4.548436625 | C4I |
| 3.010779839 | 4.547819957 | DAUDI |
| 6.020146573 | 4.547819957 | NCIH1836 |
| 3.15704371 | 4.546585829 | JHUEM2 |
| 4.109360559 | 4.543495883 | OCILY3 |
| 1.90303827 | 4.541019153 | NCIH1915 |
| 3.468583317 | 4.539158811 | NALM19 |
| 2.370164281 | 4.538538164 | NCIH1650 |
| 3.825785627 | 4.538538164 | 639V |
| 3.784503983 | 4.537296067 | HCSC1 |
| 1.480265122 | 4.532940288 | DLD1 |
| 1.070389328 | 4.526694846 | CCLFUPGI0054T |
| 2.801158656 | 4.522306893 | SNU739 |
| 2.244887059 | 4.521678952 | KNS60 |
| 2.229587923 | 4.520422249 | NCIH596 |
| 2.440952198 | 4.520422249 | HS578T |
| 1.03562391 | 4.519793486 | SNU475 |
| 4.523561956 | 4.518535139 | SUDHL1 |
| 2.750606505 | 4.517905554 | SNUC5 |
| 1.063502942 | 4.517275693 | HARA |
| 1.599317794 | 4.516645558 | SNU16 |
| 2.195347598 | 4.508428653 | KMBC2 |
| 3.047887329 | 4.506525779 | NCIH1975 |
| 1.5360529 | 4.504620392 | PK45H |
| 2.752748591 | 4.504620392 | MFE280 |
| 2.933572638 | 4.500164679 | NCIH2087 |
| 1.744161096 | 4.498250868 | AM38 |
| 3.930737338 | 4.497612366 | CHP212 |
| 5.417852515 | 4.497612366 | KELLY |
| 1.891419187 | 4.496973581 | GAMG |
| 0.871843649 | 4.495055528 | SW1088 |
| 3.673556424 | 4.495055528 | LS |
| 1.974529312 | 4.492494152 | SNU216 |
| 0.097610797 | 4.491853096 | KMRC20 |
| 2.260025656 | 4.488000771 | MORCPR |
| 2.403267722 | 4.484138131 | HUT102 |
| 3.285402219 | 4.482848283 | A673 |
| 1.316145742 | 4.479618608 | HCC2279 |
| 1.992768431 | 4.479618608 | 647V |
| 2.769771739 | 4.471837762 | SNU1 |
| 2.12763328 | 4.47118746 | U2904 |
| 1.333423734 | 4.470536865 | SNB75 |
| 2.35614381 | 4.468583317 | SNU1544 |
| 1.361768359 | 4.46662712 | SNU685 |
| 1.659924558 | 4.46662712 | A2058 |
| 1.195347598 | 4.465974465 | T3M4 |
| 3.030336078 | 4.464668267 | CAL78 |
| 1.974529312 | 4.464014725 | NCIH1437 |
| 4.196134881 | 4.463360886 | LU165 |
| 2.364572432 | 4.46139759 | STM9101 |
| 1.695993813 | 4.459431619 | GOS3 |
| 1.063502942 | 4.458119481 | LS411N |
| 1.604071324 | 4.455491621 | ICC15 |
| 5.040454121 | 4.452200049 | NCIH2227 |
| 5.881419908 | 4.452200049 | NCIH1963 |
| 3.820689561 | 4.451540833 | CORL23 |
| 2.837943242 | 4.450881315 | GB1 |
| 0.263034406 | 4.442280035 | HT29 |
| 6.37086174 | 4.442280035 | ECC10 |
| 5.568640195 | 4.441616269 | NCIH1618 |
| 1.10433666 | 4.436961338 | T98G |
| 1.163498732 | 4.43629512 | SMSCTR |
| 1.86393845 | 4.43629512 | KNS81 |
| 3.680324357 | 4.43496176 | CHLA99 |
| 2.488000771 | 4.430954271 | COLO320 |
| 1.286881148 | 4.430285273 | DOTC24510 |
| 1.269033146 | 4.429615964 | NCIH292 |
| 2.518535139 | 4.427606173 | BFTC905 |
| 5.700717133 | 4.42693562 | CORL279 |
| 0.956056652 | 4.424922088 | NCIH2405 |
| 3.754887502 | 4.424250286 | D458 |
| 5.479295243 | 4.422233001 | NCIH2171 |
| 4.120186028 | 4.420886575 | SBC5 |
| 3.070389328 | 4.418189948 | G402 |
| 2.097610797 | 4.417515003 | HCC38 |
| 2.939226578 | 4.411426246 | MON |
| 1.803227036 | 4.410748128 | HNT34 |
| 2.657640005 | 4.408711861 | MKN1 |
| 3.019701914 | 4.408711861 | RAJI |
| 3.541019153 | 4.408711861 | SUPT11 |
| 1.618238656 | 4.405311683 | MSTO211H |
| 2.353323291 | 4.405311683 | NP2 |
| 1.735522177 | 4.402585758 | NUGC3 |
| 3.845991771 | 4.402585758 | OVK18 |
| 1.691534165 | 4.399854674 | SCS214 |
| 1.622930351 | 4.39848719 | IGROV1 |
| 4.669593751 | 4.397118409 | WERIRB1 |
| 1.97819563 | 4.396433531 | HSC4 |
| 2.021479727 | 4.396433531 | OCILY132 |
| 1.50589093 | 4.395748328 | PK8 |
| 4.063502942 | 4.393004257 | DL |
| 1.937344392 | 4.392317423 | COV318 |
| 3.005399988 | 4.392317423 | HCC1806 |
| 2.185866545 | 4.390254956 | U178 |
| 3.056583528 | 4.389566812 | NCIH650 |
| 2.739848103 | 4.387500406 | SKMES1 |
| 2.028569152 | 4.386121157 | HOS |
| 2.15704371 | 4.385431037 | DAOY |
| 3.379898164 | 4.385431037 | IGR37 |
| 2.821710215 | 4.381283373 | TTC709 |
| 1.22650853 | 4.378511623 | MHHCALL3 |
| 3.070389328 | 4.378511623 | U937 |
| 1.286881148 | 4.375734539 | NCIH747 |
| 0.948600847 | 4.375039431 | DBTRG05MG |
| 1.500802053 | 4.368069877 | HCC461 |
| 2.867896464 | 4.367371066 | CAOV3 |
| 3.906890596 | 4.365972428 | TOV112D |
| 0.333423734 | 4.363871925 | LNZ308 |
| 2.378511623 | 4.363171077 | NCIH2172 |
| 0.454175893 | 4.362469889 | SW982 |
| 2.86393845 | 4.361768359 | KHM1B |
| 1.232660757 | 4.355439197 | 8305C |
| 3.106013238 | 4.354028938 | DEL |
| 3.152183419 | 4.353323291 | KYSE270 |
| 0.189033824 | 4.348374075 | C4II |
| 4.410069692 | 4.34553831 | CORL95 |
| 0.659924558 | 4.344828497 | PANFR0233 |
| 1.618238656 | 4.339137385 | MDAMB231 |
| 0.432959407 | 4.337711092 | LP1 |
| 1.220329955 | 4.336997417 | CAPAN2 |
| 1.269033146 | 4.336283388 | KYSE30 |
| 1.485426827 | 4.336283388 | SW900 |
| 2.801158656 | 4.334854269 | PF382 |
| 2.5360529 | 4.331991778 | TE1 |
| 2.49057013 | 4.331275267 | EM2 |
| 1.49057013 | 4.3305584 | KP3 |
| 2.042644337 | 4.3305584 | PC9 |
| 2.03562391 | 4.327687364 | LOVO |
| 4.363171077 | 4.326968712 | PEER |
| 4.274261661 | 4.324810603 | SCLC22H |
| 3.196921734 | 4.321928095 | K562 |
| 2.498250868 | 4.315421316 | SNU81 |
| 1.459431619 | 4.313245852 | HCC1428 |
| 0.622930351 | 4.312519967 | UMUC13 |
| 1.316145742 | 4.304511042 | 143B |
| 4.184280294 | 4.303780748 | MC116 |
| 3.263034406 | 4.299391206 | HCT116 |
| 3.401903472 | 4.299391206 | TYKNU |
| 5.198494154 | 4.298658316 | NCIH2081 |
| 1.097610797 | 4.297925053 | GCT |
| 6.72900887 | 4.297191417 | SCLC21H |
| 3.279471296 | 4.294253136 | SNU398 |
| 1.063502942 | 4.292045492 | ACHN |
| 1.739848103 | 4.292045492 | KMS28PE |
| 2.956056652 | 4.292045492 | NALM16 |
| 1.831877241 | 4.290571851 | CHLA266 |
| 3.819668183 | 4.290571851 | HUTU80 |
| 1.565597176 | 4.289834465 | SNU719 |
| 1.887525271 | 4.283921772 | UBLC1 |
| 3.838951767 | 4.28169825 | LAN2 |
| 4.751142325 | 4.28169825 | NCIH524 |
| 3.039138394 | 4.280956314 | COGN278 |
| 3.173127433 | 4.280956314 | TALL1 |
| 1.220329955 | 4.278728213 | SNU407 |
| 1.372952098 | 4.276496666 | SNUC4 |
| 1.50589093 | 4.275752049 | HCS2 |
| 1.739848103 | 4.275752049 | SNU626 |
| 1.765534746 | 4.275752049 | HCC4006 |
| 2.269033146 | 4.275752049 | 42MGBA |
| 1.66448284 | 4.267535798 | DV90 |
| 1.189033824 | 4.266036894 | HS611T |
| 1.169925001 | 4.263785614 | RT112 |
| 1.480265122 | 4.263785614 | NALM1 |
| 3.147306699 | 4.256255873 | COV434 |
| 1.803227036 | 4.255500733 | A375SKINCJ3 |
| 1.049630768 | 4.253989266 | LU65 |
| 1.941106311 | 4.253989266 | EFO27 |
| 2.160274831 | 4.253989266 | LCAM1 |
| 1.007195501 | 4.253232939 | NCIH322 |
| 1.459431619 | 4.252476214 | SU8686 |
| 2.070389328 | 4.252476214 | YKG1 |
| 2.23878686 | 4.251719093 | HMC18 |
| 1.163498732 | 4.246408087 | MCC142 |
| 0.50589093 | 4.244125943 | JHOC5 |
| 2.003602237 | 4.243364426 | CHAGOK1 |
| 1.871843649 | 4.241840184 | FU97 |
| 2.726831217 | 4.241840184 | SUDHL6 |
| 0.310340121 | 4.241077458 | T84 |
| 0.5360529 | 4.240314329 | HKA1 |
| 1.40053793 | 4.239550797 | OSRC2 |
| 0.704871964 | 4.237257771 | KARPAS422 |
| 0.650764559 | 4.23572706 | SSP25 |
| 3.553360503 | 4.233427944 | PFSK1 |
| 1.700439718 | 4.232660757 | SKGT4 |
| 1.007195501 | 4.231893162 | A4FUK |
| 1.996388746 | 4.231125158 | LCLC103H |
| 2.687060688 | 4.230356745 | MDAMB157 |
| 0.432959407 | 4.22881869 | KMS27 |
| 0.992768431 | 4.22881869 | 59M |
| 0.604071324 | 4.228049048 | OVCAR5 |
| 2.715893371 | 4.227278994 | CMK115 |
| 3.393690764 | 4.22650853 | ISHIKAWAHERAKLIO02ER |
| 5.46727948 | 4.224966365 | NCIH889 |
| 2.03562391 | 4.224194664 | HUT78 |
| 0.659924558 | 4.22342255 | TUHR4TKB |
| 1.687060688 | 4.218781168 | RPMI7951 |
| 2.914564523 | 4.218781168 | NCIH838 |
| 1.244887059 | 4.21800615 | UO31 |
| 1.704871964 | 4.21800615 | HCC1569 |
| 1.895302621 | 4.21800615 | ICC10 |
| 1.967168608 | 4.216454865 | CL14 |
| 0.695993813 | 4.207111961 | MERO48A |
| 1.195347598 | 4.207111961 | HEKTE |
| 2.416839742 | 4.205548911 | RERFLCMS |
| 2.914564523 | 4.205548911 | NCIH1793 |
| 0.807354922 | 4.204766751 | SMZ1 |
| 3.835924074 | 4.204766751 | EW8 |
| 1.469885976 | 4.201633861 | RPMI2650 |
| 1.049630768 | 4.200849575 | HPAC |
| 1.15704371 | 4.200849575 | K029AX |
| 1.970853654 | 4.200849575 | SNU449 |
| 3.666756592 | 4.198494154 | SCCOHT1 |
| 4.155425432 | 4.192194165 | SKES1 |
| 0.495695163 | 4.19061486 | HCC70 |
| 2.66448284 | 4.19061486 | GA10 |
| 2.726831217 | 4.19061486 | SKNO1 |
| 0.50589093 | 4.189033824 | GDM1 |
| 1.678071905 | 4.189033824 | F36P |
| 1.735522177 | 4.189033824 | MKN45 |
| 1.790772038 | 4.189033824 | A204 |
| 2.214124805 | 4.189033824 | NCIH1838 |
| 0.622930351 | 4.186659017 | JHOS2 |
| 1.10433666 | 4.186659017 | NO10 |
| 0.82374936 | 4.185073638 | OCILY12 |
| 3.03562391 | 4.182692298 | NCIH358 |
| 0.485426827 | 4.181897643 | PANC0813 |
| 1.09085343 | 4.181897643 | MPNST724 |
| 0.739848103 | 4.181102551 | L33 |
| 1.007195501 | 4.181102551 | SW579 |
| 2.82374936 | 4.18030702 | 127399 |
| 5.288358562 | 4.18030702 | CHP134 |
| 2.754887502 | 4.17951105 | A375SKINCJ1 |
| 1.475084883 | 4.175524601 | NCIH460 |
| 1.232660757 | 4.174725988 | ICC5 |
| 1.321928095 | 4.173926932 | HSOS1 |
| 0.22650853 | 4.169925001 | SNU5 |
| 2.759155834 | 4.169925001 | SKMEL1 |
| 0.895302621 | 4.169123281 | PRECLH |
| 1.117695043 | 4.168321116 | JHESOAD1 |
| 1.333423734 | 4.168321116 | HTMMT |
| 4.775050541 | 4.168321116 | IMR32 |
| 0.807354922 | 4.166715445 | 2313287 |
| 1.13093087 | 4.166715445 | A253 |
| 0.321928095 | 4.165107985 | SNU387 |
| 0.925999419 | 4.162693432 | SW780 |
| 1.220329955 | 4.161887682 | CH157MN |
| 1.021479727 | 4.159467729 | SF539 |
| 1.63691458 | 4.159467729 | KMS21BM |
| 1.89917563 | 4.159467729 | BT16 |
| 6.038699551 | 4.156234798 | COLO684 |
| 0.575312331 | 4.155425432 | WM2664 |
| 0.214124805 | 4.154615611 | RT4 |
| 1.851998837 | 4.154615611 | PC14 |
| 0.516015147 | 4.153805336 | TT1TKB |
| 0.713695815 | 4.153805336 | NCIH2170 |
| 1.40599236 | 4.151371776 | TE15 |
| 2.124328135 | 4.151371776 | PSN1 |
| 2.201633861 | 4.150559677 | KS1 |
| 0.070389328 | 4.14974712 | LMSU |
| 2.032100843 | 4.14974712 | SKMEL3 |
| 1.007195501 | 4.146492307 | LN235 |
| 1.748461233 | 4.145677455 | FTC238 |
| 3.407352751 | 4.144862143 | YAMATO |
| 3.49057013 | 4.144862143 | LN319 |
| 2.422233001 | 4.14404637 | CCSW1 |
| 0.82374936 | 4.143230135 | NCIH1563 |
| 0.98550043 | 4.143230135 | SNU840 |
| 1.59454855 | 4.140778656 | PSS131R |
| 1.176322773 | 4.138323004 | BT12 |
| 4.211012193 | 4.137503524 | TGW |
| 2.513490746 | 4.136683578 | BHY |
| 1.163498732 | 4.135042286 | PK1 |
| 4.66618849 | 4.133399125 | QGP1 |
| 1.709290636 | 4.132576843 | LOXIMVI |
| 5.845239126 | 4.131754091 | NCIH2106 |
| 0.545968369 | 4.129283017 | OACM51 |
| 0.604071324 | 4.129283017 | SW626 |
| 1.40053793 | 4.126807703 | F5 |
| 0.321928095 | 4.125155131 | HS888T |
| 0.632268215 | 4.124328135 | JVE015 |
| 0.669026766 | 4.122672719 | GP5D |
| 1.992768431 | 4.121015401 | SNU182 |
| 0.687060688 | 4.120186028 | HSKTC |
| 4.228049048 | 4.120186028 | L1236 |
| 0.389566812 | 4.119356177 | MERO84 |
| 2.937344392 | 4.119356177 | KMM1 |
| 2.765534746 | 4.116031993 | ALLSIL |
| 2.13422094 | 4.115199749 | NCIH2009 |
| 1.22650853 | 4.114367025 | CCLFUPGI0012T |
| 1.214124805 | 4.112700133 | CAL148 |
| 0.516015147 | 4.111865964 | KALS1 |
| 2.62058641 | 4.110196178 | RPMI8226 |
| 3.250961574 | 4.110196178 | TANOUE |
| 0.150559677 | 4.108524457 | MP46 |
| 0.475084883 | 4.106850796 | SW1783 |
| 2.080657663 | 4.106850796 | NCIH1734 |
| 3.92314918 | 4.102658131 | SET2 |
| 4.138323004 | 4.097610797 | SNGM |
| 2.367371066 | 4.096767855 | MINO |
| 4.706530553 | 4.093391153 | NCIH1155 |
| 3.682573297 | 4.09085343 | COGE352 |
| 1.807354922 | 4.08236197 | HB1119 |
| 5.535741941 | 4.081510068 | JURKAT |
| 1.970853654 | 4.080657663 | NCCSTCK140 |
| 5.276496666 | 4.080657663 | NCIH146 |
| 0.739848103 | 4.078097423 | SKRC20 |
| 0.992768431 | 4.078097423 | MM485 |
| 1.327687364 | 4.075532631 | HCC78 |
| 1.744161096 | 4.075532631 | SF767 |
| 0.925999419 | 4.074676686 | HS729 |
| 0.713695815 | 4.073820233 | FADU |
| 3.270528942 | 4.070389328 | 22RV1 |
| 1.250961574 | 4.068670811 | OCIMY5 |
| 1.925999419 | 4.068670811 | NCIH2004RT |
| 3.7322692 | 4.067810784 | ECC12 |
| 1.422233001 | 4.066950244 | JHOM2B |
| 1.914564523 | 4.06608919 | UACC893 |
| 2.280956314 | 4.065227623 | 253JBV |
| 2.813524689 | 4.065227623 | NCIH1299 |
| 1.333423734 | 4.06436554 | MUTZ5 |
| 1.316145742 | 4.062639828 | KYSE180 |
| 2.358958826 | 4.06091205 | NCIH3255 |
| 2.792855352 | 4.060047384 | OVCAR8 |
| 2.821710215 | 4.060047384 | UACC62SKINCJ1 |
| 1.124328135 | 4.059182199 | HCA1 |
| 2.307428525 | 4.059182199 | GMS10 |
| 0.722466024 | 4.057450272 | SNU201 |
| 0.485426827 | 4.056583528 | SNU61 |
| 1.014355293 | 4.055716264 | TGBC18TKB |
| 1.063502942 | 4.054848477 | MCC26 |
| 1.847996907 | 4.054848477 | OVISE |
| 2.23572706 | 4.053980168 | NCIH1573 |
| 3.436961338 | 4.052241981 | NAMALWA |
| 1.097610797 | 4.050501697 | BL70 |
| 0.5360529 | 4.049630768 | JOPACA1 |
| 0.613531653 | 4.048759312 | BT474 |
| 1.786596362 | 4.047887329 | JHH1 |
| 2.195347598 | 4.04701482 | SNU308 |
| 3.023255352 | 4.04701482 | HEC251 |
| 0.604071324 | 4.046141782 | NUGC2 |
| 1.232660757 | 4.044394119 | LAMA84 |
| 4.394376945 | 4.044394119 | COGN305 |
| 1.22650853 | 4.036503334 | KYSE410 |
| 2.861955364 | 4.03562391 | NCIH1703 |
| 1.432959407 | 4.034743949 | TCCSUP |
| 1.316145742 | 4.033863452 | LN464 |
| 4.874305166 | 4.031218731 | KCL22 |
| 0.516015147 | 4.030336078 | SNU620 |
| 3.060047384 | 4.029452886 | TTC1240 |
| 5.223808659 | 4.029452886 | CORL47 |
| 1.731183242 | 4.027684877 | BL41 |
| 1.50589093 | 4.023255352 | RKN |
| 0.622930351 | 4.022367813 | NMCG1 |
| 4.871350841 | 4.022367813 | NMB |
| 0.831877241 | 4.021479727 | RKO |
| 1.111031312 | 4.021479727 | SNU478 |
| 0.59454855 | 4.020591095 | YD38 |
| 3.046141782 | 4.017921908 | OV56 |
| 3.418189948 | 4.017031081 | COLO668 |
| 1.944858446 | 4.016139703 | YD8 |
| 1.269033146 | 4.012568674 | WM793 |
| 2.869871406 | 4.010779839 | LNCAPCLONEFGC |
| 5.108942569 | 4.009884589 | SKNBE2 |
| 1.22650853 | 4.004501392 | M059K |
| 1.443606651 | 4 | HCC827 |
| 0.807354922 | 3.998195503 | SNU489 |
| 2.364572432 | 3.997292408 | DOHH2 |
| 2.025028794 | 3.993674362 | COLO679 |
| 2.853995647 | 3.993674362 | OVKATE |
| 1.867896464 | 3.991861931 | ASH3 |
| 1.49057013 | 3.99004722 | SAT |
| 2.327687364 | 3.989139007 | RI1 |
| 0.941106311 | 3.987320866 | PANFR0368 |
| 0.565597176 | 3.98458935 | WM115 |
| 0.82374936 | 3.9800253 | WM1799 |
| 1.50589093 | 3.9800253 | HSC2 |
| 3.459431619 | 3.979110755 | DMS114 |
| 2.114367025 | 3.976363636 | HCC364 |
| 0.545968369 | 3.975446766 | NCIH684 |
| 3.3950628 | 3.975446766 | MESSA |
| 1.049630768 | 3.974529312 | KMRC3 |
| 0.367371066 | 3.972692654 | CAS1 |
| 0.765534746 | 3.971773447 | SUM159PT |
| 0.176322773 | 3.970853654 | CI |
| 0.22650853 | 3.970853654 | HS939T |
| 2.217230716 | 3.969933275 | NCIH2023 |
| 1.469885976 | 3.969012308 | KASUMI2 |
| 0.344828497 | 3.965322548 | C75 |
| 1.516015147 | 3.964398632 | ICC108 |
| 0.807354922 | 3.963474124 | SAS |
| 2.229587923 | 3.962549023 | NUDUL1 |
| 1.815575429 | 3.961623328 | MG63 |
| 0.613531653 | 3.959770155 | HT1080 |
| 0.687060688 | 3.959770155 | SNU1105 |
| 0.704871964 | 3.959770155 | HEC265 |
| 2.809414444 | 3.959770155 | HMCB |
| 0.464668267 | 3.953265239 | SALE |
| 0.790772038 | 3.952333566 | RS411 |
| 2.007195501 | 3.951401292 | SNU869 |
| 3.363171077 | 3.951401292 | MDAMB453 |
| 0.782408565 | 3.949534933 | RH36 |
| 5.95373085 | 3.948600847 | MHHNB11 |
| 1.996388746 | 3.94673086 | MDST8 |
| 2.166715445 | 3.94673086 | HEC116 |
| 2.025028794 | 3.944858446 | JHOM1 |
| 4.350497247 | 3.943921327 | SKNDZ |
| 3.57167681 | 3.942983598 | MTA |
| 1.773996325 | 3.94204526 | JHUEM1 |
| 2.646162657 | 3.94204526 | CHLA57 |
| 0.948600847 | 3.941106311 | CAL851 |
| 0.963474124 | 3.941106311 | OV90 |
| 0.722466024 | 3.939226578 | VMCUB1 |
| 1.432959407 | 3.938285792 | HEL9217 |
| 2.198494154 | 3.937344392 | OCIC4P |
| 0.941106311 | 3.934516502 | COV504 |
| 0.956056652 | 3.934516502 | OAW42 |
| 2.010779839 | 3.934516502 | HGC27 |
| 0.695993813 | 3.931683057 | MUTZ3 |
| 2.739848103 | 3.930737338 | HCC1500 |
| 1.070389328 | 3.929790998 | HOP62 |
| 1.786596362 | 3.927896454 | IGR39 |
| 1.799087306 | 3.927896454 | CL11 |
| 0.895302621 | 3.925049965 | SNU8 |
| 1.021479727 | 3.924099886 | OE33 |
| 3.340562269 | 3.915520901 | P31FUJ |
| 2.12763328 | 3.912649865 | CAMA1 |
| 0.070389328 | 3.911691582 | H157 |
| 0.516015147 | 3.909773104 | RPE1SS6 |
| 1.257010618 | 3.909773104 | RH18 |
| 3.107687869 | 3.908812908 | IOMMLEE |
| 1.622930351 | 3.905928478 | JMSU1 |
| 2.160274831 | 3.905928478 | EKVX |
| 0.23878686 | 3.904965719 | TO175T |
| 0.773996325 | 3.904002316 | JVM2 |
| 1.695993813 | 3.90303827 | KOPN8 |
| 3.15704371 | 3.90014226 | ZR751 |
| 0.389566812 | 3.898208353 | CCLFUPGI0040T |
| 0.622930351 | 3.898208353 | KNS62 |
| 0.111031312 | 3.897240426 | HKBMM |
| 0.790772038 | 3.897240426 | ASPC1 |
| 1.344828497 | 3.897240426 | LC1SQSF |
| 0.40053793 | 3.895302621 | SW1990 |
| 0.98550043 | 3.893362211 | CHL1DM |
| 0.739848103 | 3.891419187 | NCIH3122 |
| 1.722466024 | 3.891419187 | SKHEP1 |
| 3.032100843 | 3.888499736 | MAC2A |
| 1.361768359 | 3.887525271 | 697 |
| 0.214124805 | 3.886550147 | SNU1272 |
| 1.50589093 | 3.886550147 | A549 |
| 2.62058641 | 3.886550147 | SNU1077 |
| 0.632268215 | 3.885574364 | SNU283 |
| 3.125981654 | 3.879705766 | NCIH2286 |
| 0.526068812 | 3.876762491 | BECKER |
| 4.866907978 | 3.876762491 | CADOES1 |
| 0.895302621 | 3.874796966 | SNU410 |
| 1.855989697 | 3.874796966 | SW1710 |
| 0.839959587 | 3.870857864 | LPS27 |
| 0.613531653 | 3.869871406 | MOLT16 |
| 1.555816155 | 3.869871406 | SW1417 |
| 2.392317423 | 3.869871406 | HCC1171 |
| 1.257010618 | 3.868884273 | MAVER1 |
| 0.641546029 | 3.867896464 | RPE1SS48 |
| 2.414135533 | 3.867896464 | GI1 |
| 0.90303827 | 3.866907978 | OV7 |
| 3.40053793 | 3.866907978 | HCC2157 |
| 4.35614381 | 3.866907978 | LU134A |
| 2.608809243 | 3.865918815 | OSC20 |
| 0.124328135 | 3.860962798 | HSC3 |
| 2.625270489 | 3.858975614 | ONCODG1 |
| 1.137503524 | 3.857980995 | MOLP8 |
| 4.028569152 | 3.85698569 | D341Med |
| 1.704871964 | 3.855989697 | MMAC |
| 0.150559677 | 3.850999395 | SEMK2 |
| 0.189033824 | 3.850999395 | C10 |
| 3.03562391 | 3.849999259 | JHH4 |
| 1.604071324 | 3.84899843 | YD10B |
| 3.999098034 | 3.845991771 | KYO1 |
| 4.164303583 | 3.843983844 | SKNMC |
| 1.659924558 | 3.841973119 | SKMEL5 |
| 1.599317794 | 3.839959587 | SH10TC |
| 3.628773595 | 3.839959587 | DMS273 |
| 0.847996907 | 3.838951767 | ICC2 |
| 1.333423734 | 3.837943242 | AU565 |
| 0.298658316 | 3.836934011 | OCUM1 |
| 0.111031312 | 3.833902077 | HS606T |
| 0.526068812 | 3.832890014 | KM12 |
| 0.176322773 | 3.831877241 | HS934T |
| 3.609991295 | 3.831877241 | NH12 |
| 4.457462965 | 3.831877241 | JURLMK1 |
| 0.695993813 | 3.830863757 | MM127 |
| 1.23878686 | 3.830863757 | 786O |
| 0.584962501 | 3.82984956 | MEL285 |
| 0.704871964 | 3.828834649 | UMUC14 |
| 2.140778656 | 3.826802684 | NCCMPNST1C1 |
| 3.404630684 | 3.825785627 | TE8 |
| 1.416839742 | 3.82374936 | UACC62 |
| 2.15704371 | 3.820689561 | FARAGE |
| 0.124328135 | 3.815575429 | LS513 |
| 5.62556274 | 3.815575429 | KPNYN |
| 0.214124805 | 3.814550423 | HS343T |
| 1.709290636 | 3.814550423 | KKU213 |
| 2.003602237 | 3.813524689 | SNU324 |
| 2.192194165 | 3.813524689 | CAOV4 |
| 2.176322773 | 3.812498225 | SW1271 |
| 0.189033824 | 3.806324057 | NHAHTDD |
| 0.948600847 | 3.806324057 | EBC1 |
| 2.531069493 | 3.802193217 | OCIAML5 |
| 2.989139007 | 3.802193217 | KYSE140 |
| 2.485426827 | 3.801158656 | KD |
| 0.321928095 | 3.797012978 | H103 |
| 0.879705766 | 3.797012978 | MDAMB361 |
| 1.189033824 | 3.797012978 | CCLFPEDS0001T |
| 2.169925001 | 3.797012978 | OS252 |
| 0.879705766 | 3.795974694 | MERO41 |
| 1.137503524 | 3.795974694 | HUO9 |
| 1.438292852 | 3.795974694 | CAKI2 |
| 2.495695163 | 3.794935663 | HEC151 |
| 0.782408565 | 3.791814071 | YD15 |
| 2.469885976 | 3.789729251 | NCIH2887 |
| 1.007195501 | 3.788685711 | EHEB |
| 0.310340121 | 3.786596362 | HS739T |
| 2.933572638 | 3.782408565 | VAESBJ |
| 3.077242999 | 3.781359714 | SNU638 |
| 1.163498732 | 3.780310099 | PLB985 |
| 3.980939266 | 3.780310099 | SHP77 |
| 4.107687869 | 3.780310099 | SEKI |
| 0.713695815 | 3.77925972 | A101D |
| 2.427606173 | 3.778208576 | G361 |
| 0.687060688 | 3.775050541 | CL34 |
| 2.61117238 | 3.772941338 | IPC298 |
| 0.5360529 | 3.770829046 | UMUC5 |
| 1.378511623 | 3.770829046 | RCHACV |
| 0.815575429 | 3.768713657 | NH93T |
| 2.582556003 | 3.768713657 | HEC1B |
| 2.475084883 | 3.766595162 | HCC1359 |
| 0.250961574 | 3.765534746 | SLVL |
| 1.550900665 | 3.764473551 | ICC6 |
| 4.050501697 | 3.761285273 | KYM1 |
| 1.748461233 | 3.759155834 | 5637 |
| 1.214124805 | 3.752748591 | SNU668 |
| 1.500802053 | 3.752748591 | T3M3 |
| 0.150559677 | 3.751677946 | HS281T |
| 2.639232163 | 3.750606505 | PANC0213 |
| 4.702657543 | 3.750606505 | NCO2 |
| 2.678071905 | 3.7473874 | M07E |
| 1.803227036 | 3.746312766 | JHH5 |
| 3.554588852 | 3.745237332 | KMS34 |
| 1.454175893 | 3.743084056 | HEMCSS |
| 4.768184325 | 3.74092756 | TT |
| 3.228049048 | 3.738767837 | SUMB002 |
| 1.344828497 | 3.737686761 | RAMOS |
| 3.301587647 | 3.736604875 | HCC1395 |
| 0.50589093 | 3.733354341 | PANC0327 |
| 2.378511623 | 3.733354341 | MDAMB436 |
| 1.232660757 | 3.7322692 | HKGZCC |
| 1.344828497 | 3.731183242 | IGR1 |
| 1.292781749 | 3.726831217 | PACADD137 |
| 1.316145742 | 3.726831217 | HCC56 |
| 1.608809243 | 3.724650272 | HMY1 |
| 0.59454855 | 3.722466024 | NCIH1944 |
| 0.367371066 | 3.721372659 | KCIMOH1 |
| 0.378511623 | 3.71918344 | ISTMES1 |
| 2.914564523 | 3.71918344 | JMURTK2 |
| 0.59454855 | 3.716990894 | HSPSS |
| 1.304511042 | 3.716990894 | HEPG2 |
| 2.485426827 | 3.716990894 | SH4 |
| 1.599317794 | 3.715893371 | PANC0403 |
| 1.650764559 | 3.714795011 | MOLM6 |
| 2.587364991 | 3.709290636 | NIHOVCAR3 |
| 2.060047384 | 3.708187236 | OE19 |
| 0.084064265 | 3.707082992 | HS229T |
| 0.516015147 | 3.707082992 | ES2 |
| 3.051372102 | 3.704871964 | A3KAW |
| 1.627606838 | 3.701549057 | ML1 |
| 1.304511042 | 3.699329526 | YSCCC |
| 0.378511623 | 3.698218478 | HUPT4 |
| 3.316145742 | 3.697106574 | MFE319 |
| 0.485426827 | 3.695993813 | PECAPJ15 |
| 0.678071905 | 3.694880193 | NO36 |
| 4.266786541 | 3.694880193 | GOTO |
| 1.263034406 | 3.69265037 | A388 |
| 1.316145742 | 3.69265037 | SNU886 |
| 2.56315813 | 3.69265037 | A375SKINCJ2 |
| 0.163498732 | 3.687060688 | HS821T |
| 1.111031312 | 3.683696454 | MELJUSO |
| 2.185866545 | 3.683696454 | FEPD |
| 3.279471296 | 3.680324357 | NCIH2444 |
| 2.577730931 | 3.679198571 | EFM19 |
| 2.121015401 | 3.678071905 | NCIH1792 |
| 1.007195501 | 3.675815931 | SW480 |
| 5.860962798 | 3.67468662 | NCIH1694 |
| 0.189033824 | 3.673556424 | NCIH226 |
| 1.163498732 | 3.672425342 | CCLFUPGI0011T |
| 0.40053793 | 3.669026766 | HS274T |
| 0.584962501 | 3.669026766 | HEYA8 |
| 2.599317794 | 3.666756592 | SKM1 |
| 2.922197848 | 3.659924558 | AML193 |
| 1.555816155 | 3.658782734 | FTC133 |
| 0.839959587 | 3.656496371 | KO52 |
| 0.298658316 | 3.655351829 | HS840T |
| 1.378511623 | 3.655351829 | MEL290 |
| 0.495695163 | 3.653060017 | LS1034 |
| 3.86393845 | 3.651912745 | CHLA15 |
| 0.495695163 | 3.650764559 | BICR78 |
| 3.147306699 | 3.650764559 | KIJK |
| 0.084064265 | 3.646162657 | RPE1SS77 |
| 1.726831217 | 3.645009884 | CAPAN1 |
| 0.565597176 | 3.64038956 | PECAPJ49 |
| 3.659924558 | 3.639232163 | SHSY5Y |
| 0.333423734 | 3.634593268 | CAL29 |
| 0.613531653 | 3.63343121 | PANC1005 |
| 1.726831217 | 3.629939409 | SNU878 |
| 0.555816155 | 3.627606838 | HT55 |
| 0.35614381 | 3.626439137 | DKMG |
| 0.731183242 | 3.624100895 | TE11 |
| 0.565597176 | 3.622930351 | MERO82 |
| 1.516015147 | 3.622930351 | NP3 |
| 2.375734539 | 3.621758857 | SNU213 |
| 0.650764559 | 3.617063344 | HCC515 |
| 2.066950244 | 3.615887074 | NCIH2291 |
| 2.40053793 | 3.615887074 | MFM223 |
| 1.367371066 | 3.61117238 | COV413A |
| 0.35614381 | 3.609991295 | ISTMES2 |
| 3.488000771 | 3.609991295 | SKMEL19 |
| 0.464668267 | 3.608809243 | T173 |
| 1.570462931 | 3.608809243 | HS695T |
| 2.454175893 | 3.607626221 | MEL270 |
| 3.346247774 | 3.607626221 | HHUA |
| 3.339137385 | 3.605257263 | TC71 |
| 0.584962501 | 3.604071324 | C32 |
| 0.275007047 | 3.602884409 | SW1116 |
| 1.339137385 | 3.601696516 | PANC0203 |
| 0.475084883 | 3.600507645 | TE125T |
| 3.370164281 | 3.599317794 | BLUE1 |
| 0.443606651 | 3.59812696 | SLR24 |
| 0.214124805 | 3.596935142 | 94T778 |
| 0.495695163 | 3.596935142 | KYSE450 |
| 1.316145742 | 3.596935142 | MEWO |
| 2.007195501 | 3.596935142 | HUH28 |
| 1.339137385 | 3.595742339 | NCIH2122 |
| 1.82374936 | 3.592158002 | RERFLCKJ |
| 0.933572638 | 3.587364991 | ECC2 |
| 2.163498732 | 3.587364991 | PC3 |
| 0.189033824 | 3.580145484 | HS675T |
| 0.310340121 | 3.580145484 | SNU520 |
| 2.007195501 | 3.580145484 | MM426 |
| 2.438292852 | 3.578938713 | NCIH23 |
| 0.879705766 | 3.577730931 | SNU1327 |
| 2.364572432 | 3.576522138 | CHLA218 |
| 1.748461233 | 3.575312331 | HS294T |
| 2.659924558 | 3.572889668 | NH84T |
| 2.010779839 | 3.570462931 | BT483 |
| 0.992768431 | 3.568032105 | ICC106 |
| 1.176322773 | 3.566815154 | TDOTT |
| 0.097610797 | 3.565597176 | SLR26 |
| 0.790772038 | 3.564378169 | LO68 |
| 0.389566812 | 3.56315813 | CCLFUPGI0009T |
| 0.432959407 | 3.560714954 | SW403 |
| 0.782408565 | 3.560714954 | ANGMCSS |
| 2.077242999 | 3.554588852 | SW156 |
| 0.250961574 | 3.553360503 | HS172T |
| 3.545968369 | 3.553360503 | NCIH810 |
| 0.632268215 | 3.550900665 | WM88 |
| 3.260025656 | 3.550900665 | A427 |
| 0.286881148 | 3.548436625 | ICC4 |
| 3.390942773 | 3.547203025 | HH |
| 2.744161096 | 3.545968369 | MIAPACA2 |
| 0.933572638 | 3.543495883 | SCC4 |
| 0.263034406 | 3.539779192 | COLO800 |
| 0.948600847 | 3.538538164 | TCCPAN2 |
| 2.35614381 | 3.538538164 | JHH2 |
| 2.384049807 | 3.537296067 | UPMD1 |
| 3.462052319 | 3.537296067 | BEN |
| 2.084064265 | 3.534808661 | EPLC272H |
| 0.298658316 | 3.533563348 | H357 |
| 1.459431619 | 3.533563348 | ACCMESO1 |
| 2.857980995 | 3.529820947 | KPNSI9S |
| 4.300855871 | 3.529820947 | SKPNDW |
| 3.66448284 | 3.528571319 | HS936T |
| 1.66448284 | 3.527320608 | RERFLCAD2 |
| 1.655351829 | 3.524815928 | UMUC9 |
| 3.030336078 | 3.522306893 | M040416 |
| 0.35614381 | 3.519793486 | IPMBO056 |
| 2.244887059 | 3.516015147 | NZM3 |
| 3.860962798 | 3.513490746 | U266B1 |
| 0.855989697 | 3.512226887 | PACADD161 |
| 2.811471031 | 3.512226887 | SKGIIIA |
| 0.137503524 | 3.509695842 | HS737T |
| 1.195347598 | 3.509695842 | SCC25 |
| 1.859969548 | 3.50589093 | LU99 |
| 2.189033824 | 3.50589093 | EMTOKA |
| 3.488000771 | 3.502075956 | KARPAS299 |
| 0.124328135 | 3.500802053 | HS688AT |
| 0.748461233 | 3.49441561 | BICR16 |
| 0.910732662 | 3.49441561 | M140325 |
| 3.089159132 | 3.493134922 | CALU6 |
| 0.378511623 | 3.491853096 | SNU1041 |
| 0.176322773 | 3.488000771 | TE159T |
| 0.475084883 | 3.482848283 | TE9 |
| 1.718087584 | 3.481557281 | 253J |
| 0.454175893 | 3.480265122 | CAL27 |
| 2.98550043 | 3.476381688 | 8MGBA |
| 0.310340121 | 3.475084883 | HLFA |
| 0.389566812 | 3.473786912 | A704 |
| 1.13093087 | 3.469885976 | HOP92 |
| 0.757023247 | 3.464668267 | RCK8 |
| 0.321928095 | 3.463360886 | HS618T |
| 3.764473551 | 3.460742564 | OV17R |
| 0.298658316 | 3.458119481 | HS852T |
| 5.648177796 | 3.455491621 | LU135 |
| 1.807354922 | 3.454175893 | NCIH211 |
| 0.286881148 | 3.452858965 | SW1463 |
| 3.802193217 | 3.444932049 | NB1 |
| 0.641546029 | 3.443606651 | RPE1SS119 |
| 0.815575429 | 3.443606651 | EFE184 |
| 0.298658316 | 3.442280035 | COLO794 |
| 1.448900951 | 3.442280035 | UMUC1 |
| 0.40053793 | 3.436961338 | PL21 |
| 0.584962501 | 3.436961338 | NCIH196 |
| 3.061776198 | 3.43162296 | MOLT3 |
| 1.189033824 | 3.430285273 | PK59 |
| 1.963474124 | 3.430285273 | CCLP1 |
| 2.10433666 | 3.428946345 | HCC202 |
| 1.220329955 | 3.426264755 | HUH1 |
| 1.704871964 | 3.424922088 | BFTC909 |
| 0.124328135 | 3.423578171 | MM383 |
| 1.786596362 | 3.416839742 | KLE |
| 0.50589093 | 3.410069692 | UPCISCC040 |
| 3.363171077 | 3.408711861 | SUDHL4 |
| 1.459431619 | 3.40599236 | HELA |
| 1.545968369 | 3.403267722 | CCLFUPGI0027T |
| 1.163498732 | 3.401903472 | SG231 |
| 4.938285792 | 3.40053793 | SIMA |
| 0.150559677 | 3.399171094 | SNU245 |
| 1.15704371 | 3.3950628 | SKMEL2 |
| 0.879705766 | 3.393690764 | NCCLMS1C1 |
| 1.695993813 | 3.392317423 | COLO678 |
| 2.298658316 | 3.392317423 | TE4 |
| 1.432959407 | 3.386810946 | ECGI10 |
| 3.588564737 | 3.385431037 | NCIH854 |
| 0.378511623 | 3.379898164 | HS600T |
| 0.389566812 | 3.375734539 | SUM229PE |
| 2.253989266 | 3.374343989 | HCC1599 |
| 0.713695815 | 3.370164281 | JEG3 |
| 0.773996325 | 3.368768349 | SHMAC5 |
| 2.95419631 | 3.365972428 | KMH2 |
| 0.150559677 | 3.363171077 | HS863T |
| 0.526068812 | 3.363171077 | LPS510 |
| 1.339137385 | 3.363171077 | HT115 |
| 0.23878686 | 3.361768359 | HS616T |
| 0.613531653 | 3.357552005 | COLO201 |
| 4.250961574 | 3.35614381 | NB1643 |
| 3.204766751 | 3.351910961 | TC205 |
| 1.669026766 | 3.349082146 | KG1C |
| 3.724650272 | 3.347665656 | D283MED |
| 0.765534746 | 3.346247774 | VP229 |
| 1.220329955 | 3.346247774 | H413 |
| 0.111031312 | 3.343407822 | RPE1SS111 |
| 1.220329955 | 3.340562269 | MAPACHS77 |
| 0.50589093 | 3.339137385 | NCIH1666 |
| 0.286881148 | 3.337711092 | KMRC2 |
| 5.167919866 | 3.336283388 | MDAMB134VI |
| 0.704871964 | 3.334854269 | LPS067 |
| 1.589763487 | 3.327687364 | COLO829 |
| 0.263034406 | 3.326249701 | MERO95 |
| 2.014355293 | 3.326249701 | KP363T |
| 0.847996907 | 3.324810603 | RCM1 |
| 2.711494907 | 3.324810603 | NCIH716 |
| 0.124328135 | 3.323370069 | HS860T |
| 1.070389328 | 3.321928095 | OCIAML3 |
| 3.40053793 | 3.321928095 | SKNMM |
| 0.321928095 | 3.320484678 | PANC0504 |
| 2.344828497 | 3.313245852 | MELHO |
| 0.799087306 | 3.305970521 | S117 |
| 1.454175893 | 3.305970521 | L428 |
| 2.147306699 | 3.303050085 | SNU349 |
| 0.910732662 | 3.294253136 | UMUC16 |
| 0.070389328 | 3.292781749 | HS940T |
| 1.316145742 | 3.292781749 | EOL1 |
| 1.831877241 | 3.292781749 | HCC1419 |
| 2.316145742 | 3.292781749 | SKGT2 |
| 2.217230716 | 3.29130886 | VMRCRCW |
| 2.163498732 | 3.289834465 | OMM25 |
| 2.523561956 | 3.286881148 | LN443 |
| 0.35614381 | 3.282439805 | UMUC6 |
| 0.641546029 | 3.282439805 | HT144SKINFV1 |
| 3.060047384 | 3.282439805 | HDQP1 |
| 2.653060017 | 3.270528942 | OMM1 |
| 0.713695815 | 3.269033146 | MERO83 |
| 2.691534165 | 3.267535798 | CHLA90 |
| 4.030336078 | 3.267535798 | CTV1DM |
| 3.152183419 | 3.263034406 | NOZ |
| 1.344828497 | 3.250961574 | HUNS1 |
| 2.931683057 | 3.250961574 | NCIH2110 |
| 1.40053793 | 3.241840184 | NCIH1435 |
| 0.389566812 | 3.240314329 | MDAMB415 |
| 1.613531653 | 3.240314329 | A172 |
| 2.629939409 | 3.23878686 | MM253 |
| 1.541019153 | 3.237257771 | KOSC2 |
| 1.906890596 | 3.237257771 | CAL54 |
| 0.23878686 | 3.23572706 | KMCH1 |
| 1.22650853 | 3.23572706 | RS5 |
| 1.871843649 | 3.232660757 | BHT101 |
| 0.443606651 | 3.22650853 | SKMEL24 |
| 2.689299161 | 3.22650853 | MALME3M |
| 1.220329955 | 3.224966365 | MEG01 |
| 2.211012193 | 3.224966365 | OCIC5X |
| 0.367371066 | 3.221877081 | WM983B |
| 0.669026766 | 3.221877081 | HT144 |
| 1.35614381 | 3.218781168 | ONDA9 |
| 1.5360529 | 3.217230716 | PANFR0420 |
| 2.097610797 | 3.217230716 | SNU1033 |
| 0.422233001 | 3.198494154 | HS698T |
| 0.97819563 | 3.196921734 | SNU1079 |
| 0.40053793 | 3.193771743 | WPE1NA22 |
| 1.15704371 | 3.192194165 | NCIH1869 |
| 1.469885976 | 3.19061486 | SUM149PT |
| 0.454175893 | 3.189033824 | HS834T |
| 1.427606173 | 3.189033824 | SNU1197 |
| 0.40053793 | 3.184280294 | SKCO1 |
| 1.925999419 | 3.182692298 | MM160113 |
| 1.384049807 | 3.181102551 | WM4235 |
| 2.516015147 | 3.181102551 | KPL1 |
| 5.228049048 | 3.181102551 | CHP126 |
| 1.321928095 | 3.17951105 | NCIH2347 |
| 1.257010618 | 3.176322773 | SUM190PT |
| 0.111031312 | 3.173127433 | C396 |
| 0.765534746 | 3.173127433 | HT144SKINFV3 |
| 1.541019153 | 3.173127433 | MCF7 |
| 1.757023247 | 3.173127433 | TGBC52TKB |
| 1.163498732 | 3.171527106 | BICR31 |
| 0.695993813 | 3.169925001 | T3M5 |
| 1.10433666 | 3.169925001 | HUH7 |
| 2.232660757 | 3.169925001 | LCLC97TM1 |
| 3.548436625 | 3.166715445 | SUPT1 |
| 0.90303827 | 3.165107985 | OSC19 |
| 2.358958826 | 3.163498732 | KML1 |
| 0.163498732 | 3.15704371 | SNUC1 |
| 1.843983844 | 3.148934105 | CCLFUPGI0015T |
| 0.201633861 | 3.14404637 | CCLFCORE0002T |
| 2.508428653 | 3.142413438 | SKNAS |
| 0.163498732 | 3.139142019 | A431 |
| 1.427606173 | 3.122672719 | UHO1 |
| 2.901108243 | 3.121015401 | RVH421 |
| 0.687060688 | 3.117695043 | U343 |
| 0.807354922 | 3.117695043 | KE37 |
| 0.565597176 | 3.111031312 | CFPAC1 |
| 0.176322773 | 3.10433666 | PECAPJ34CLONEC12 |
| 0.432959407 | 3.102658131 | UPCISCC131 |
| 0.659924558 | 3.09423607 | ONDA7 |
| 2.10433666 | 3.09085343 | MPP89 |
| 0.23878686 | 3.087462841 | UPCISCC074 |
| 1.14404637 | 3.084064265 | 9505BIK |
| 1.389566812 | 3.08236197 | P2URK562 |
| 0.790772038 | 3.080657663 | SHI1 |
| 2.313245852 | 3.080657663 | OCILY18 |
| 2.916476644 | 3.075532631 | NCIH1373 |
| 0.910732662 | 3.068670811 | SKGI |
| 1.049630768 | 3.066950244 | HCC2218 |
| 0.333423734 | 3.063502942 | HEC1 |
| 1.669026766 | 3.063502942 | KASUMI6 |
| 2.659924558 | 3.063502942 | HSSCH2 |
| 0.084064265 | 3.060047384 | SCABER |
| 0.443606651 | 3.060047384 | KON |
| 0.35614381 | 3.058316496 | BCP1 |
| 0.59454855 | 3.056583528 | EFM192A |
| 0.925999419 | 3.056583528 | SKMEL30 |
| 1.925999419 | 3.056583528 | CHLA06ATRT |
| 2.166715445 | 3.056583528 | CCLFPEDS0003T |
| 0.782408565 | 3.054848477 | RCC10RGB |
| 0.704871964 | 3.051372102 | ZR7530 |
| 0.201633861 | 3.044394119 | NCIH28 |
| 0.604071324 | 3.042644337 | HT3 |
| 0.464668267 | 3.040892431 | MS751 |
| 0.124328135 | 3.037382222 | OVTOKO |
| 0.23878686 | 3.037382222 | GB2 |
| 1.659924558 | 3.037382222 | IPMBO055 |
| 0.22650853 | 3.033863452 | SJSA1 |
| 0.084064265 | 3.032100843 | DETROIT562 |
| 0.333423734 | 3.032100843 | TL1 |
| 0.765534746 | 3.032100843 | CCLFUPGI0085T |
| 0.286881148 | 3.030336078 | TE10 |
| 1.201633861 | 3.030336078 | THP1 |
| 0.201633861 | 3.026800059 | JVE127 |
| 1.111031312 | 3.026800059 | HT144SKINFV2 |
| 0.933572638 | 3.025028794 | NOMO1 |
| 0.35614381 | 3.021479727 | MUTZ8 |
| 0.189033824 | 3.019701914 | PACADD188 |
| 2.292781749 | 3.010779839 | RO82W1 |
| 0.555816155 | 2.992768431 | SNU466 |
| 3.621758857 | 2.992768431 | MM415 |
| 2.107687869 | 2.99095486 | CA922 |
| 4.388878339 | 2.983677695 | DU4475 |
| 0.970853654 | 2.981852653 | G415 |
| 2.464668267 | 2.976363636 | KU1919 |
| 0.90303827 | 2.972692654 | HS255T |
| 1.86393845 | 2.952333566 | HEL |
| 1.90303827 | 2.944858446 | SKMEL28 |
| 1.201633861 | 2.931683057 | P4E6 |
| 1.416839742 | 2.9202933 | CAKI1 |
| 0.641546029 | 2.914564523 | NZM7 |
| 0.084064265 | 2.906890596 | HS895T |
| 1.584962501 | 2.893362211 | HCC1143 |
| 3.12763328 | 2.889473543 | HCC1833 |
| 0.286881148 | 2.881664619 | HPAFII |
| 3.736604875 | 2.869871406 | TO14 |
| 1.321928095 | 2.867896464 | SLR21 |
| 0.516015147 | 2.861955364 | PEO1 |
| 0.070389328 | 2.849999259 | CCLFUPGI0101T |
| 0.411426246 | 2.849999259 | J82 |
| 0.432959407 | 2.845991771 | UACC812 |
| 4.681449265 | 2.835924074 | HMVII |
| 0.286881148 | 2.833902077 | ICC9 |
| 2.589763487 | 2.833902077 | MM370 |
| 1.757023247 | 2.82984956 | SNU761 |
| 0.839959587 | 2.825785627 | CALU3 |
| 1.207892852 | 2.82374936 | GIMEN |
| 0.59454855 | 2.815575429 | OCIP5X |
| 3.08236197 | 2.813524689 | MONOMAC6 |
| 0.432959407 | 2.801158656 | TUHR14TKB |
| 0.807354922 | 2.801158656 | LN229 |
| 2.269033146 | 2.801158656 | MOLM13 |
| 0.495695163 | 2.797012978 | PECAPJ41CLONED2 |
| 2.841973119 | 2.794935663 | HEP3B217 |
| 0.545968369 | 2.792855352 | SCC15 |
| 1.195347598 | 2.792855352 | SKBR3 |
| 1.292781749 | 2.788685711 | A498 |
| 1.500802053 | 2.784503983 | SUM52PE |
| 1.790772038 | 2.782408565 | CASKI |
| 1.794935663 | 2.773996325 | 921 |
| 2.50589093 | 2.754887502 | OCIM2 |
| 2.518535139 | 2.752748591 | HUPT3 |
| 0.250961574 | 2.750606505 | OE21 |
| 0.124328135 | 2.742006211 | YUHOIN0650 |
| 0.748461233 | 2.739848103 | CII |
| 3.528571319 | 2.739848103 | HTCC3 |
| 1.541019153 | 2.733354341 | JK1 |
| 1.220329955 | 2.731183242 | OVMANA |
| 0.310340121 | 2.720278465 | CL40 |
| 1.316145742 | 2.711494907 | HOTHC |
| 2.195347598 | 2.707082992 | COV362 |
| 0.97819563 | 2.702657543 | JHUEM7 |
| 0.070389328 | 2.695993813 | KMRC1 |
| 3.367371066 | 2.693765712 | ICC3 |
| 0.432959407 | 2.687060688 | C125PM |
| 0.59454855 | 2.673556424 | OCIAML2 |
| 1.220329955 | 2.673556424 | C84 |
| 1 | 2.666756592 | SNU1196 |
| 0.575312331 | 2.650764559 | NCIH508 |
| 0.150559677 | 2.64385619 | UPCISCC200 |
| 0.475084883 | 2.634593268 | WM3772F |
| 1.575312331 | 2.618238656 | MYLA |
| 0.454175893 | 2.606442228 | RMGI |
| 0.799087306 | 2.599317794 | MV411 |
| 3.472487771 | 2.59454855 | TN2 |
| 1.815575429 | 2.580145484 | P12ICHIKAWA |
| 1.292781749 | 2.575312331 | MDAMB175VII |
| 2.087462841 | 2.575312331 | MEL202 |
| 2.339137385 | 2.565597176 | CORL105 |
| 2.46727948 | 2.560714954 | BOKU |
| 2.192194165 | 2.555816155 | HS944T |
| 0.941106311 | 2.5360529 | HOKUG |
| 0.839959587 | 2.523561956 | PANFR0402 |
| 0.22650853 | 2.518535139 | HS742T |
| 1.056583528 | 2.518535139 | SF172 |
| 0.86393845 | 2.516015147 | UPCISCC154 |
| 0.214124805 | 2.513490746 | BICR56 |
| 0.097610797 | 2.503348735 | PACADD135 |
| 0.321928095 | 2.500802053 | 769P |
| 1.344828497 | 2.472487771 | EGI1 |
| 0.782408565 | 2.44625623 | KARPAS1718 |
| 0.464668267 | 2.443606651 | WAOSEL |
| 0.871843649 | 2.443606651 | SKGII |
| 1.948600847 | 2.435628594 | NCIH1395 |
| 1.485426827 | 2.419538892 | HCC44 |
| 1.794935663 | 2.414135533 | RVH421SKINFV1 |
| 0.722466024 | 2.411426246 | ICC12 |
| 2.207892852 | 2.3950628 | KASUMI1 |
| 0.124328135 | 2.375734539 | PEO4 |
| 1.646162657 | 2.370164281 | KKU100 |
| 1.163498732 | 2.35614381 | CW2 |
| 0.565597176 | 2.353323291 | SKMEL31 |
| 0.773996325 | 2.353323291 | ONS76 |
| 3.184280294 | 2.319039816 | NGP |
| 0.411426246 | 2.316145742 | MERO14 |
| 2.304511042 | 2.310340121 | NZM42 |
| 4.699329526 | 2.289834465 | NCIH1385 |
| 0.722466024 | 2.283921772 | JL1 |
| 1.891419187 | 2.269033146 | UMRC7 |
| 0.40053793 | 2.250961574 | CJM |
| 0.641546029 | 2.23878686 | NUGC4 |
| 0.263034406 | 2.232660757 | UPCISCC116 |
| 0.641546029 | 2.229587923 | BICR6 |
| 2.629939409 | 2.22650853 | TC106 |
| 2.063502942 | 2.204766751 | IPMBO053 |
| 0.23878686 | 2.192194165 | BICR22 |
| 3.303050085 | 2.182692298 | L82 |
| 0.23878686 | 2.176322773 | KHYG1 |
| 0.475084883 | 2.173127433 | ICC8 |
| 0.731183242 | 2.173127433 | RBE |
| 3.346247774 | 2.169925001 | ECC4 |
| 0.137503524 | 2.140778656 | C80 |
| 0.847996907 | 2.140778656 | COLO792 |
| 1.744161096 | 2.12763328 | SIGM5 |
| 1.589763487 | 2.117695043 | MONOMAC1 |
| 0.310340121 | 2.111031312 | HG3 |
| 0.411426246 | 2.100977648 | SUM44PE |
| 1.510961919 | 2.060047384 | NCIH2073 |
| 1.35614381 | 2.046141782 | ESO51 |
| 1.22650853 | 2.007195501 | SKN |
| 1.422233001 | 2.003602237 | HUCCT1 |
| 0.124328135 | 2 | HL60 |
| 0.137503524 | 1.992768431 | C99 |
| 0.485426827 | 1.974529312 | UPCISCC111 |
| 3.432959407 | 1.941106311 | MOLM1 |
| 3.310340121 | 1.933572638 | OCUG1 |
| 2.014355293 | 1.929790998 | HOUAI |
| 1.169925001 | 1.891419187 | KARPAS384 |
| 1.599317794 | 1.843983844 | MM386 |
| 0.731183242 | 1.827819025 | CCLFUPGI0036T |
| 2.229587923 | 1.82374936 | U87MG |
| 0.35614381 | 1.819668183 | H376 |
| 1.906890596 | 1.790772038 | SCC3 |
| 1.929790998 | 1.722466024 | VAL |
| 1.176322773 | 1.713695815 | UMRC3 |
| 0.111031312 | 1.622930351 | KMLS1 |
| 0.565597176 | 1.541019153 | OCIAML4 |
| 0.782408565 | 1.416839742 | LPS6 |
| 0.495695163 | 1.286881148 | SUM185PE |
| 1.03562391 | 1.280956314 | PACADD159 |
| 0.495695163 | 1.23878686 | PGA1 |
| 0.799087306 | 1.084064265 | MOLM14 |
| 0.739848103 | 1.03562391 | ICC137 |
| 0.22650853 | 0.831877241 | SW954 |
| 0.097610797 | 0.757023247 | SUM102PT |
| 0.042644337 | 0.722466024 | CCLFUPGI0052T |

**Supplementary Table S6.** Tumor weight and volume measured for animal study.

| **Sr. no** | **Group name** | **Sex of mice** | **Tumor volume (mm^3^)** | **Tumor weight (g)** |
| --- | --- | --- | --- | --- |
| 1 | Mock | Male | 295.488 | 1.6624 |
| 2 |  | Male | 306.128 | 1.5428 |
| 3 |  | Female | 315.171 | 1.3699 |
| 4 |  | Female | 278.216 | 1.2458 |
| 5 | USP28-KO | Male | 94.770 | 0.2259 |
| 6 |  | Male | 92.697 | 0.2502 |
| 7 |  | Female | 78.750 | 0.1450 |
| 8 |  | Female | 84.533 | 0.3845 |
| 9 | USP28-KO+ USP28 | Male | 240.944 | 1.5828 |
| 10 |  | Male | 279.276 | 1.5025 |
| 11 |  | Female | 289.098 | 1.2689 |
| 12 |  | Female | 268.324 | 1.1125 |
| 13 | USP28-KO+ MAST1 | Male | 305.809 | 1.6125 |
| 14 |  | Male | 267.411 | 1.5215 |
| 15 |  | Female | 286.875 | 1.3254 |
| 16 |  | Female | 287.557 | 1.1958 |
